# Restriction of HIV-1 Escape by a Highly Broad and Potent Neutralizing Antibody

**DOI:** 10.1016/j.cell.2020.01.010

**Published:** 2020-02-06

**Authors:** Philipp Schommers, Henning Gruell, Morgan E. Abernathy, My-Kim Tran, Adam S. Dingens, Harry B. Gristick, Christopher O. Barnes, Till Schoofs, Maike Schlotz, Kanika Vanshylla, Christoph Kreer, Daniela Weiland, Udo Holtick, Christof Scheid, Markus M. Valter, Marit J. van Gils, Rogier W. Sanders, Jörg J. Vehreschild, Oliver A. Cornely, Clara Lehmann, Gerd Fätkenheuer, Michael S. Seaman, Jesse D. Bloom, Pamela J. Bjorkman, Florian Klein

**Affiliations:** 1 Laboratory of Experimental Immunology, Institute of Virology, Faculty of Medicine and University Hospital Cologne, University of Cologne, 50931 Cologne, Germany; 2 Department I of Internal Medicine, Faculty of Medicine and University Hospital Cologne, University of Cologne, 50937 Cologne, Germany; 3 German Center for Infection Research (DZIF), partner site Bonn-Cologne, 50931 Cologne, Germany; 4 Division of Biology and Biological Engineering, California Institute of Technology, Pasadena, CA 91125, USA; 5 Basic Sciences Division and Computational Biology Program, Fred Hutchinson Cancer Research Center, Seattle, WA 98109, USA; 6 Department of Gynecology and Obstetrics, Faculty of Medicine and University Hospital Cologne, University of Cologne, 50931 Cologne, Germany; 7 Department of Medical Microbiology, Amsterdam UMC, University of Amsterdam, 1105 AZ Amsterdam, the Netherlands; 8 Department of Microbiology and Immunology, Weill Medical College of Cornell University, New York, NY 10021, USA; 9 Medical Department 2, University Hospital of Frankfurt, 60590 Frankfurt, Germany; 10 Clinical Trials Centre Cologne (ZKS Köln), University of Cologne, 50935 Cologne, Germany; 11 Cologne Excellence Cluster on Cellular Stress Responses in Aging-Associated Diseases (CECAD), University of Cologne, 50931 Cologne, Germany; 12 Center for Molecular Medicine Cologne (CMMC), University of Cologne, 50931 Cologne, Germany; 13 Center for Virology and Vaccine Research, Beth Israel Deaconess Medical Center, Harvard Medical School, Boston, MA 02215, USA; 14 Howard Hughes Medical Institute, Seattle, WA 98109, USA

**Keywords:** HIV-1, broadly neutralizing antibodies, CD4 binding site, escape mutations, immunotherapy, cryogenic electron microscopy, deep mutational scanning, mutational antigenic profiling, HIV-1 escape restriction, humanized mice

## Abstract

Broadly neutralizing antibodies (bNAbs) represent a promising approach to prevent and treat HIV-1 infection. However, viral escape through mutation of the HIV-1 envelope glycoprotein (Env) limits clinical applications. Here we describe 1-18, a new V_H_1-46-encoded CD4 binding site (CD4bs) bNAb with outstanding breadth (97%) and potency (GeoMean IC_50_ = 0.048 μg/mL). Notably, 1-18 is not susceptible to typical CD4bs escape mutations and effectively overcomes HIV-1 resistance to other CD4bs bNAbs. Moreover, mutational antigenic profiling uncovered restricted pathways of HIV-1 escape. Of most promise for therapeutic use, even 1-18 alone fully suppressed viremia in HIV-1-infected humanized mice without selecting for resistant viral variants. A 2.5-Å cryo-EM structure of a 1-18-BG505_SOSIP.664_ Env complex revealed that these characteristics are likely facilitated by a heavy-chain insertion and increased inter-protomer contacts. The ability of 1-18 to effectively restrict HIV-1 escape pathways provides a new option to successfully prevent and treat HIV-1 infection.

## Highlights

• Identification of 1-18, a highly broad and potent V_H_1-46-derived CD4bs antibody
• 2.5-Å cryo-EM structure of 1-18-Env complex reveals inter-protomer contacts
• 1-18 overcomes VRC01-class resistance and restricts development of HIV-1 escape
• Monotherapy with 1-18 maintains viral suppression in HIV-1_YU2_-infected humanized mice

## Graphical Abstract

**Figure:**
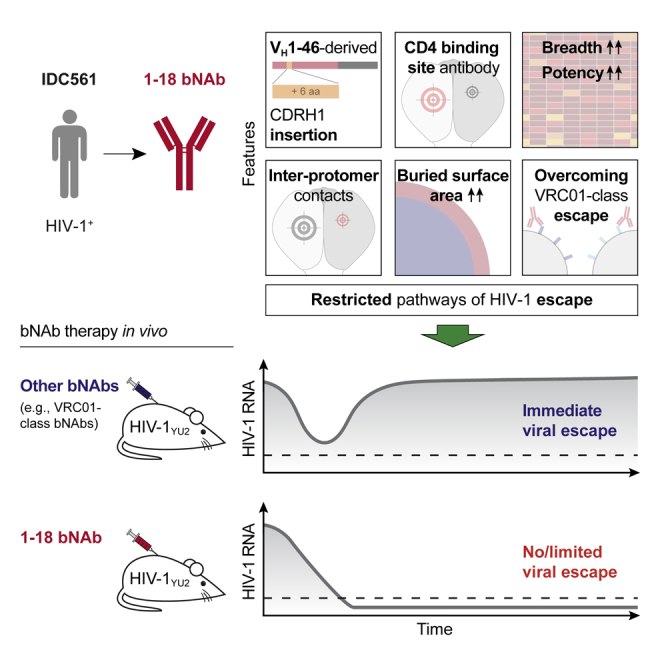

## Teaser

Broadly neutralizing antibodies targeting the HIV-1 envelope protein are a promising option for prevention and treatment of HIV-1 infection. However, development of viral resistance can limit clinical efficacy. Schommers et al. identify a highly broad and potent antibody that targets the CD4 binding site of HIV-1. Compared with other potent CD4 binding site antibodies, it restricts the development of viral escape and effectively suppresses HIV-1 *in vivo*.

## Introduction

Broadly neutralizing antibodies (bNAbs) targeting the HIV-1 envelope protein (Env) can prevent infection in animal models and are under investigation for passive immunization in clinical trials (Balazs et al., 2011, Gautam et al., 2016, Julg and Barouch, 2019, Moldt et al., 2012, Shingai et al., 2014). Moreover, bNAbs have been demonstrated to suppress viremia and delay viral rebound after interruption of antiretroviral therapy (ART) in HIV-1-infected individuals (Bar et al., 2016, Bar-On et al., 2018, Caskey et al., 2015, Caskey et al., 2017, Lynch et al., 2015a, Mendoza et al., 2018, Scheid et al., 2016). Although these results highlight the significant clinical potential of bNAbs, pre-existing or *de novo* HIV-1 resistance cause treatment failure and can strongly limit bNAb applications in humans (Bar et al., 2016, Bar-On et al., 2018, Caskey et al., 2015, Caskey et al., 2017, Lynch et al., 2015a, Mendoza et al., 2018, Scheid et al., 2016). Strategies to prevent and overcome viral escape are therefore critical to effectively implement bNAb-mediated approaches for HIV-1 prevention and therapy (Caskey et al., 2019, Gruell and Klein, 2018).

In recent years, potent bNAbs have been isolated from HIV-1-infected donors that target distinct vulnerable epitopes on the Env trimer. These epitopes include the CD4 binding site (CD4bs), the V1/V2 loop, the V3 loop glycan patch, the membrane-proximal external region (MPER), and the interface between the gp120 and gp41 subunits (Gama and Koup, 2018, Sok and Burton, 2018, Walker and Burton, 2018). Among these sites, the CD4bs is of particular interest because CD4 serves as the primary receptor for viral entry (Kwong et al., 1998, Maddon et al., 1986, Zhou et al., 2007).

Most potent CD4bs bNAbs are characterized by use of the immunoglobulin heavy-chain gene segment IGVH1-2^∗^02, high levels of somatic hypermutation, a five-residue complementarity-determining region 3 of the light chain (CDRL3), and mimicry of the CD4-Env interaction (West et al., 2012, Zhou et al., 2010, Zhou et al., 2013, Zhou et al., 2015). Named after the prototypical antibody, these antibodies are referred to as VRC01-class bNAbs (Wu et al., 2010). Additional members of this class include 3BNC117, NIH45-46, N49-P7, N6, and VRC07-523 (Huang et al., 2016a, Rudicell et al., 2014, Sajadi et al., 2018, Scheid et al., 2011). Other bNAbs that mimic CD4 binding are derived from the V_H_1-46 gene segment. However, compared with V_H_1-2-derived bNAbs, the V_H_1-46 bNAbs reported to date have lower potencies and breadth, which limits their potential for clinical use (Bonsignori et al., 2016, Gao et al., 2014, Scheid et al., 2011). For example, CH235.12, one of the best V_H_1-46-derived CD4bs antibodies, is less broad and more than 10-fold less potent than the VRC01-class bNAb N6 when tested against a large panel of HIV-1 strains (Bonsignori et al., 2016).

Accordingly, all CD4bs bNAbs that have advanced into clinical testing are members of the VRC01 class (3BNC117, N6, VRC01, and VRC07-523) (Bar et al., 2016, Bar-On et al., 2018, Caskey et al., 2015, Caskey et al., 2019, Cohen et al., 2018a, Crowell et al., 2019, Gaudinski et al., 2018, Gaudinski et al., 2019, Gruell and Klein, 2018, Ledgerwood et al., 2015, Lynch et al., 2015a, Mayer et al., 2017, Mendoza et al., 2018, Riddler et al., 2018, Scheid et al., 2016). However, although escape from VRC01 has been associated with a reduction in viral fitness (Lynch et al., 2015b), the effects of VRC01-class monotherapy are only transient and associated with emergence of viral escape variants (Bar et al., 2016, Caskey et al., 2015, Horwitz et al., 2013, Klein et al., 2012, Lynch et al., 2015a, Scheid et al., 2016).

Here we describe bNAb 1-18, a V_H_1-46-derived CD4bs antibody that exceeds the potency and breadth of most classical V_H_1-46- and V_H_1-2-derived bNAbs. The structural basis of its high activity was revealed by a single-particle cryoelectron microscopy (cryo-EM) structure of a 1-18 Fab-BG505_SOSIP.664_ Env trimer complex solved at 2.5-Å resolution. Of particular interest, compared with 3BNC117 and VRC01, the two most clinically advanced CD4bs bNAbs, 1-18 effectively restricts viral escape and maintains both neutralizing activity against VRC01-class escape variants and full viral suppression when tested in HIV-1_YU2_-infected humanized mice. Therefore, 1-18 is a highly promising candidate for antibody-mediated strategies to effectively treat and prevent HIV-1 infection.

## Results

### Identification of Potent V_H_1-46-Derived bNAbs

**Figure 1:**
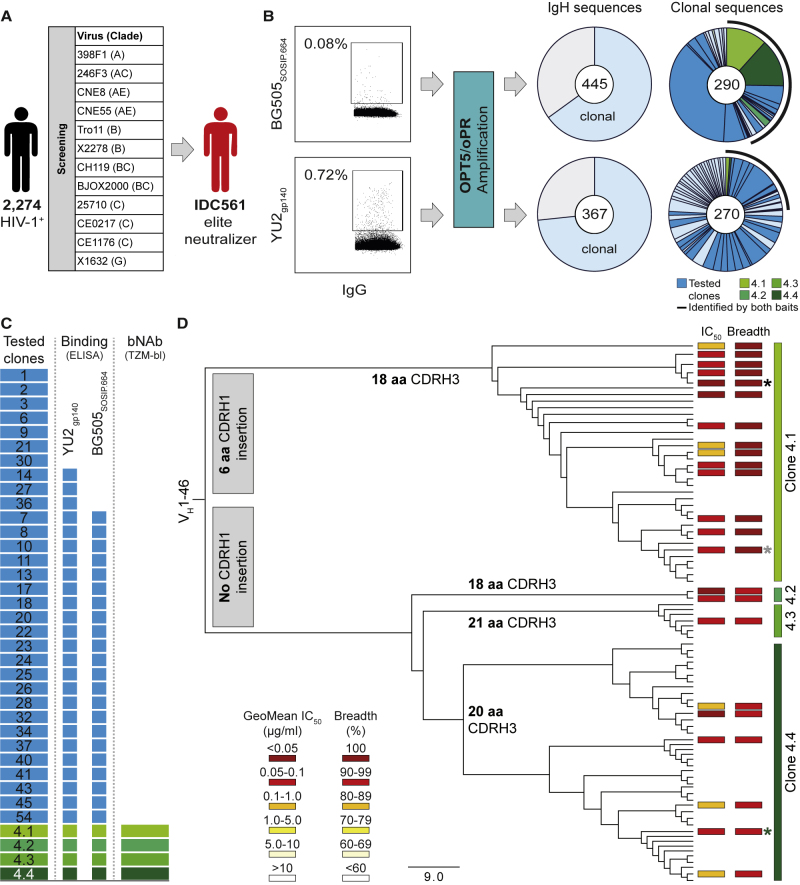
Identification of Broad and Potent Antibodies in Donor IDC561 (A) Identification of the elite neutralizer IDC561. (B) Single BG505_SOSIP_._664_- (top) or YU2_gp140_-reactive (bottom) B cells were sorted, and antibody sequences were amplified using OPT5/oPR primers. Left pie charts showing the numbers of heavy-chain sequences identified, with clonal sequences indicated in light blue; right pie charts showing the numbers of clonal heavy-chain sequences, with individual clones represented by slices. Antibodies of members of clones in dark blue and green were tested. A black line indicates clones identified by both HIV-1 Env-sorting strategies. (C) Monoclonal antibodies were produced from members of 33 clones (clone 4 comprised subclones 4.1–4.4) (left). Boxes in the middle and on the right correspond to the left panel and show antibodies binding to YU2_gp140_ or BG505_SOSIP.664_ or neutralizing more than 90% of the global panel HIV-1 strains, respectively. (D) Phylogenetic tree of clone 4 members. Boxes indicate GeoMean IC_50_ and breadth against the global panel. aa, amino acids. Black, gray, and green asterisks indicate antibodies 1-18, 1-55, and 2-12, respectively. See also Figures S1 and S2 and Tables S1, S2, S3, and S4.

**Figure S1:**
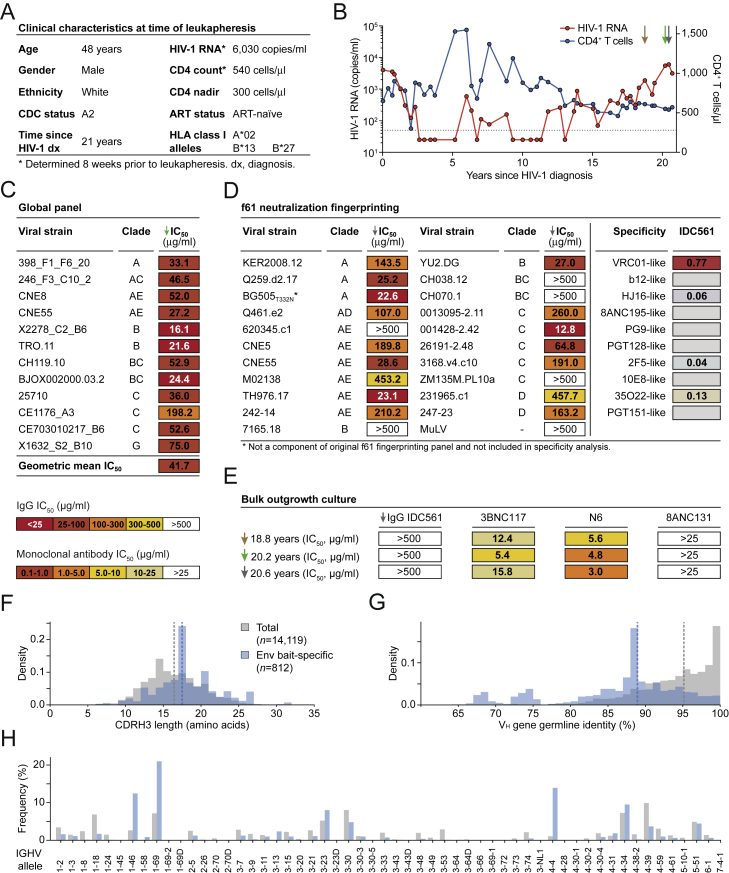
Clinical Characteristics, Neutralizing IgG Activity, and B Cell Repertoire of Individual IDC561, Related to Figure 1 (A) Clinical characteristics of IDC561 at the time of leukapheresis from which monoclonal antibodies were isolated. (B) Plasma HIV-1 RNA copies (left y axis) and CD4^+^ T cell counts (right y axis). Arrows indicate sample collections. Monoclonal antibodies were isolated from the last indicated sampling time point. Dashed line indicates HIV-1 RNA quantification limit. (C) Neutralizing activity of IDC561 serum IgG against global panel. (D) Neutralizing activity of IDC561 serum IgG against f61 fingerprinting panel and BG505_T332N_ (left, colors as in C). Right panels show delineation scores of f61 panel-based computational epitope mapping. (E) Neutralizing activity of IDC561 serum IgG and monoclonal bNAbs against outgrowth culture-derived viruses from bulk CD4^+^ T cells obtained at indicated time points (colors as in C). (F–H) Comparison of total B cell repertoire of IDC561 and Env-reactive B cells, indicating (F) CDRH3 lengths, (G) V_H_ gene germline identity, and (H) V_H_ allele distribution. Dashed lines indicate medians.

To identify individuals with elite HIV-1-neutralizing activity, we screened HIV-1-infected subjects. From each individual, purified serum or plasma immunoglobulin G (IgG) was tested for neutralizing activity in a TZM-bl cell assay against a multiclade screening panel of 12 HIV-1 pseudoviruses (deCamp et al., 2014, Sarzotti-Kelsoe et al., 2014; Figure 1A). We identified IDC561, a clade B-infected long-term non-progressor (Walker and Yu, 2013), as ranking among the top 1% of a cohort of 2,274 individuals (HIV-1-neutralizing activity at a geometric mean IC_50_ [50% inhibitory concentration] of 41.7 μg IgG/mL; Figures 1A and S1A–S1C). To characterize the epitope specificity of the IgG response, we performed neutralization fingerprinting and detected VRC01-like activity (Doria-Rose et al., 2017; Figure S1D). However, virus obtained from IDC561 was sensitive to 3BNC117 and N6, suggesting that the HIV-1-neutralizing antibodies in IDC561 differ from VRC01-class antibodies (Figure S1E).

To identify antibodies that accounted for the potent neutralizing activity of IDC561, we performed single-cell sorting of Env-reactive B cells that bound to native-like BG505_SOSIP.664_ (Sanders et al., 2013, Sliepen et al., 2015) (0.08% of IgG^+^ B cells) or to YU2_gp140_ (Scheid et al., 2009, Yang et al., 2000) (0.72% of IgG^+^ B cells) (Figure 1B). Using a new amplification strategy with primer sets optimized for precise detection of highly mutated IgG gene segments (OPT5/oPR; Kreer et al., 2019), we obtained and analyzed 812 IgG heavy-chain sequences (BG505_SOSIP.664_, n = 445; YU2_gp140_, n = 367) (Figure 1B). Compared with the total IgG^+^ B cell reservoir of IDC561, Env-reactive B cells carried slightly longer CDRH3s (median length of 17 versus 16 amino acids, p < 0.001), had higher levels of somatic mutation (median V_H_ gene nucleotide germline identity of 88.4% versus 95.3%, p < 0.001), and were enriched for the V_H_ gene segments 1-46, 1-69, and 4-4 (Figures S1F–S1H). Among Env-reactive B cells, we identified 80 B cell clones with two or more members (Figure 1B).

Following production of monoclonal antibodies (Table S1), binding of both BG505_SOSIP.664_ and YU2_gp140_ was detected by ELISA for 70% of the tested antibody clones (Figure 1C; Table S2). The antibodies of most clones showed no or minimal neutralizing activity when analyzed against the 12-strain global panel, suggesting that they play a limited role in the serum activity of IDC561 (Figure 1C; Table S3A). In contrast, all tested members (23 antibodies) of B cell clone 4 (comprising subclones 4.1–4.4) neutralized 92%–100% of viruses in the screening panel with remarkable potency (GeoMean IC_50_ of 0.032–0.198 μg/mL; Figure 1D; Table S3A). B cell clone 4, derived from the V_H_1-46 and V_Κ_3-20 gene segments, included members with different CDRH3 lengths of 18 (subclones 4.1 and 4.2), 20 (subclone 4.4), or 21 (subclone 4.3) amino acids (Table S1). Subclone 4.1 showed the highest breadth and potency and was characterized by a six-amino-acid CDRH1 insertion that lengthened the CDRH1 from 8 to 14 amino acids (Figure 1D; Tables S1 and S4). We conclude that antibodies of the V_H_1-46-derived B cell clone 4 are highly potent, broadly active, and likely mediate the neutralizing serum activity of the elite neutralizer IDC561.

### 1-18: A CD4bs bNAb with Near-Universal Breadth and Outstanding Potency

**Figure 2:**
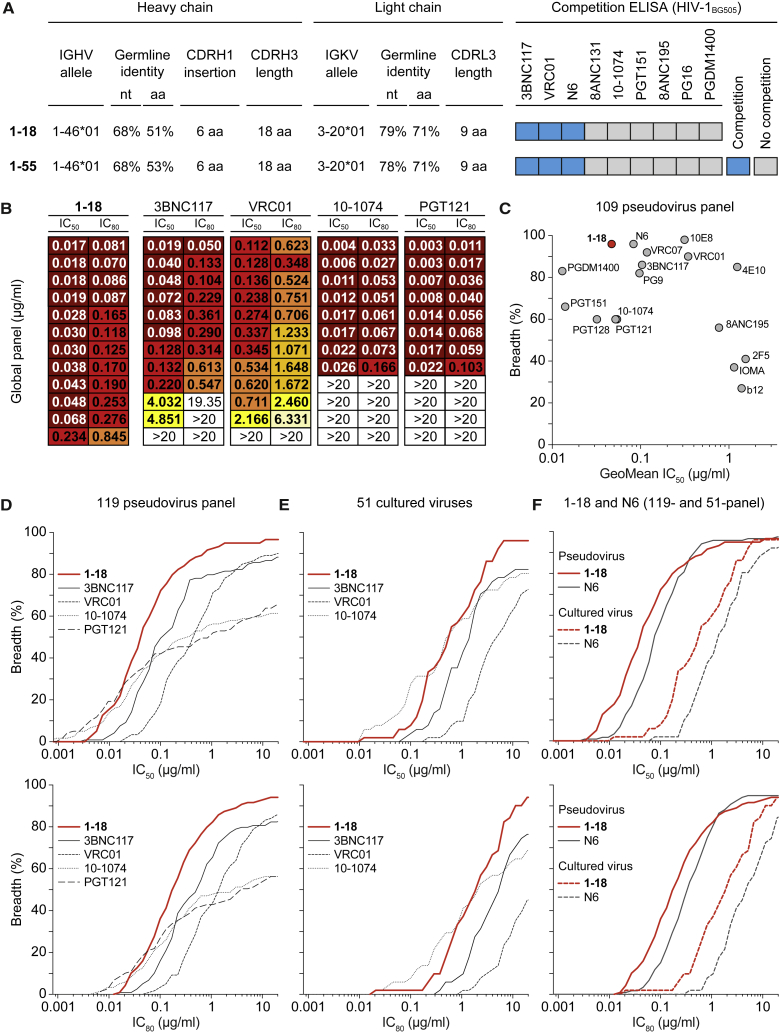
bNAb 1-18 Demonstrates Highly Potent and Near-Pan HIV-1-Neutralizing Activity (A) Characteristics of antibodies 1-18 and 1-55. (B) Activity of 1-18 against the global panel compared with bNAbs in advanced stages of clinical testing, individually sorted by increasing IC_50_ values for each bNAb. Data for 3BNC117, VRC01, 10-1074, and PGT121 were derived from CATNAP (Yoon et al., 2015). (C) Activity of 1-18 compared with a selection of bNAbs against an identical set of 109 pseudovirus strains (Yoon et al., 2015). For N6, neutralization data were determined in the same laboratory as for 1-18. (D) Activity against the 119-pseudovirus multiclade panel. Data for 3BNC117, VRC01, 10-1074, and PGT121 were derived from CATNAP (Yoon et al., 2015). (E) Activity against patient-derived bulk culture outgrowth virus. (F) Activity of 1-18 compared with N6 against the 119-pseudovirus multiclade panel and patient-derived bulk culture outgrowth viruses. In (D)–(F), IC_50_ values are shown at the top and IC_80_ values at the bottom. See also Figure S2 and Tables S3 and S5.

**Figure S2:**
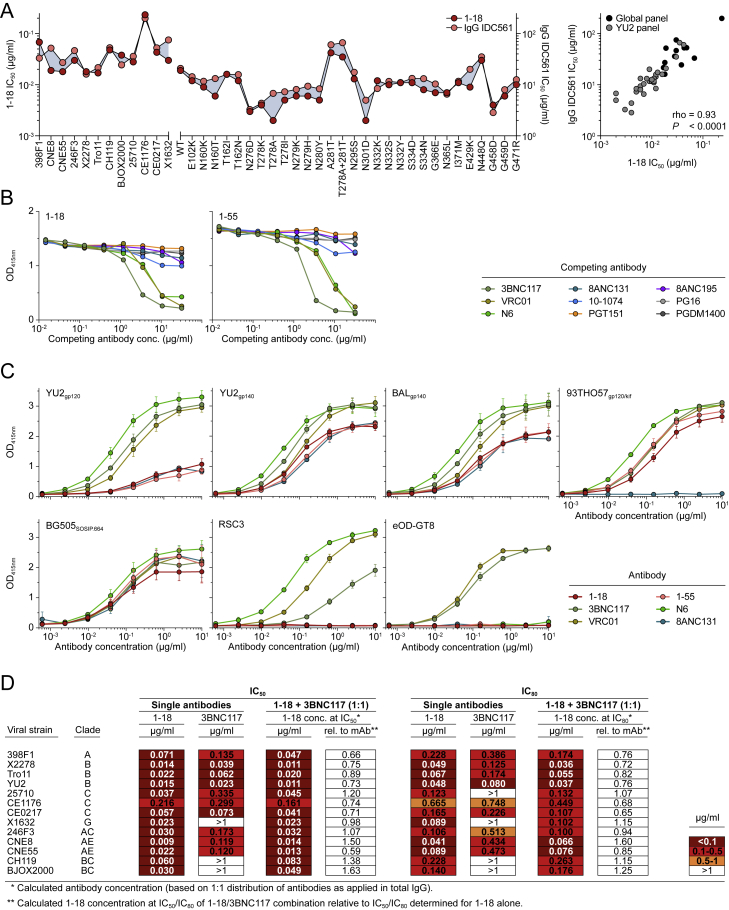
Neutralization and ELISA Binding Profiles, Related to Figures 1 and 2 (A) Left panel indicates neutralizing activity of 1-18 (left y axis) compared to serum IgG of donor IDC561 (right y axis) against the global pseudovirus panel (left x axis) and a 30-strain YU2 pseudovirus mutant panel (right x axis), with pseudoviruses indicated on the x axis. Right panel indicates correlation and calculated Spearman’s rho. (B) Competition ELISAs indicating binding of 1-18 (left) and 1-55 (right) to BG505_SOSIP.664_ following an incubation with increasing concentrations of the indicated competing antibodies. (C) ELISAs of 1-18, 1-55, and additional CD4bs antibodies against the indicated HIV-1 Env antigens. Circles show means and error bars indicate standard deviation. (D) Neutralizing activity of 1-18, 3BNC117, and the combination of both (mixed at a 1:1 ratio) against the global pseudovirus panel and YU2. Single antibodies were tested up to a concentration of 1 μg/ml, the combination up to a concentration of 2 μg/ml (total IgG amount).

We selected antibodies 561_01_18 and 561_01_55 (hereafter referred to as 1-18 and 1-55), two representative members of clone 4.1, for further analyses (Figure 2A). Both antibodies are highly mutated, with heavy and light chain V gene germline nucleotide sequence identities of 68% and 78%–79%, respectively (Figure 2A; Table S4). Notably, the neutralizing activities of 1-18 and serum IgG of IDC561 against 42 pseudoviruses strongly correlated, suggesting that members of clone 4.1 are main contributors to the serum activity of IDC561 (Figure S2A). To determine binding of 1-18 and 1-55 to the BG505_SOSIP.664_ trimer in the presence of other HIV-1 bNAbs, we performed competition ELISAs and detected interference with 3BNC117, N6, and VRC01 (Figures 2A; S2B). However, compared with these VRC01-class CD4bs antibodies, a different binding pattern was detected for 1-18 and 1-55 when tested by ELISA against several Env proteins. For example, whereas 3BNC117, N6, and VRC01 bound similarly to YU2_gp120_, YU2_gp140_, and BAL_gp140_ and were reactive to the V_1_-V_3_ loop-deficient gp120 variant RSC3 (Wu et al., 2010), bNAbs 1-18 and 1-55 showed lower (YU2_gp120,_ YU2_gp140_, and BAL_gp140_) or no (RSC3) binding to these proteins (Figure S2C). Therefore, 1-18 and 1-55 target the CD4bs but recognize this epitope differently than VRC01-class antibodies.

We next evaluated the neutralizing activity of 1-18 in detail. In comparison with four bNAbs in advanced stages of clinical investigation (3BNC117, VRC01, 10-1074, and PGT121), 1-18 demonstrated superior activity with high potency (GeoMean IC_50_ of 0.035 μg/mL, GeoMean IC_80_ [80% inhibitory concentration] of 0.155 μg/mL) against all viruses of the 12-strain global screening panel (deCamp et al., 2014; Figure 2B; Table S3A). Although 1-18 competed with other CD4bs bNAbs for binding to BG505_SOSIP.664_ by ELISA, no reduction in neutralizing activity was detected when 1-18 and 3BNC117 were combined (Figure S2D). To confirm the results of the screening panel, we evaluated the activity of 1-18 on extended pseudovirus panels. Overall, 1-18 ranked among the best bNAbs that are currently available (Figure 2C). When tested against a 119-strain multiclade panel, 1-18 showed highly potent activity (GeoMean IC_50_ of 0.048 μg/mL, GeoMean IC_80_ of 0.183 μg/mL) with a breadth of 97% (Figure 2D; Table S5A). In addition, 1-18 demonstrated high potency (GeoMean IC_50_ of 0.074 μg/mL, GeoMean IC_80_ of 0.279 μg/mL) and breadth (90%) when tested against a 100-strain clade C panel (Table S5B). Finally, we determined the activity of 1-18 against culture-derived primary HIV-1 strains that are generally more difficult to neutralize than pseudoviruses (Cohen et al., 2018b). Against viruses obtained from 51 HIV-1-infected individuals, 1-18 demonstrated higher breadth and/or potency (GeoMean IC_50_ of 0.56 μg/mL, GeoMean IC_80_ of 1.57 μg/mL, 96% breadth) than 3BNC117, VRC01, 10-1074, and PGDM1400 (Figure 2E; Table S5C) and was superior to the near-pan-neutralizing V_H_1-2-derived CD4bs bNAb N6 (Huang et al., 2016a; Figure 2F; Table S5C).

We conclude that 1-18 is a highly broad and potent V_H_1-46-derived antibody that rivals or exceeds the activity of CD4bs bNAbs described to date.

### 1-18 Targets the CD4bs and Regions of the Adjacent gp120 Protomer

**Figure 3:**
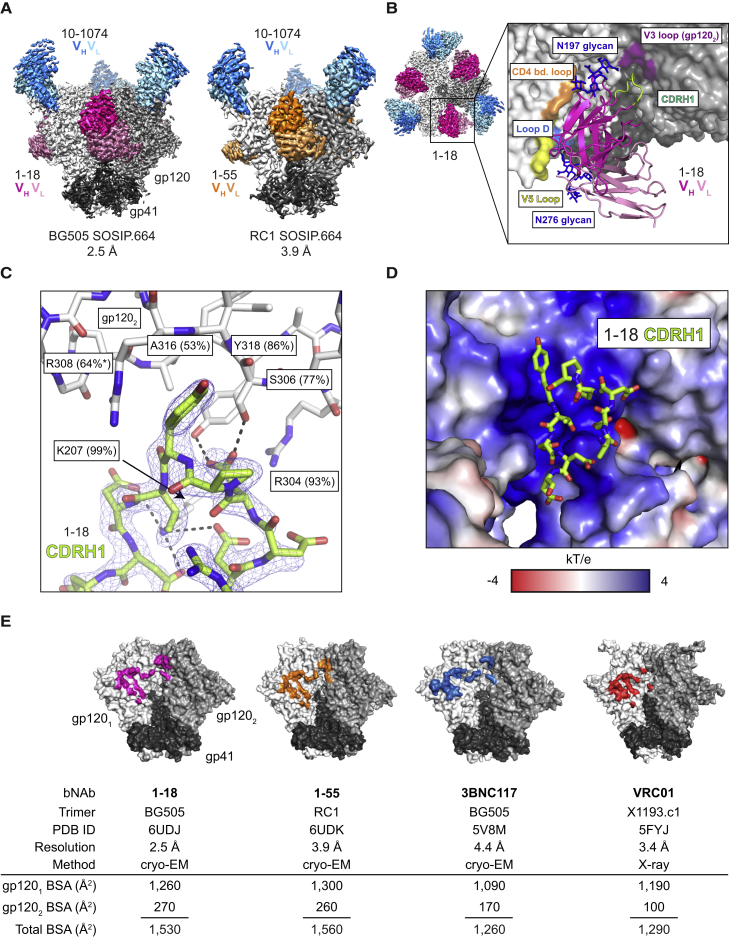
Cryo-EM Structures of 1-18 and 1-55 Fab Complexes with Env Trimers (A) EM densities for side views of Env trimers complexed with 1-18 or 1-55 Fabs and 10-1074 bNAb Fabs. 1-55 Fabs were based on sequence variants that contained primer-induced mutations at the start and end of the V genes (total of 2 aa [V_H_] and 4 aa [V_κ_] mutations). (B) Top view of 1-18-BG505-10-1074 complex density. The inset shows a close up of the interactions between the 1-18 V_H_-V_L_ domains (cartoon representation) and Env, with primary gp120 shown in light gray and secondary gp120 (gp120_2_) shown in dark gray. Protein regions that are contacted by 1-18 are shown as colored surfaces, and glycans are shown as sticks. (C) Close up of interactions of 1-18 CDRH1 residues with residues on secondary gp120. Hydrogen bonds and electrostatic contacts are shown as dotted lines. The percent conservation among Env sequences of gp120_2_ residues contacted by CDRH1 is indicated in parentheses (West et al., 2013). ^∗^ denotes the conservation percentage in the 500 viruses that have residue 308. (D) Electrostatic surface representation of the Env region contacted by the 1-18 CDRH1. (E) Buried surface areas from CD4bs bNAb contacts on the primary (gp120_1_) and secondary (gp120_2_) protomers. Env trimer structures are SOSIP.664 versions of the indicated Env strains. See also Figures S3 and S4 and Table S6.

**Figure S3:**
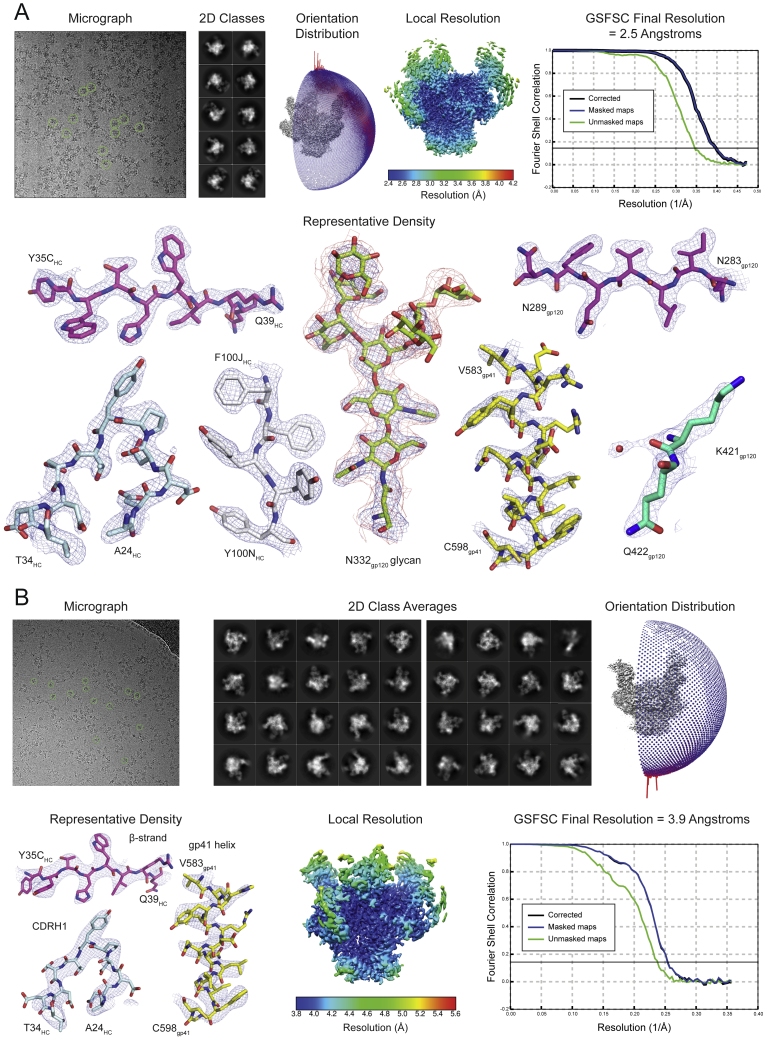
Cryo-EM Data Collection and Processing, Related to Figure 3 (A–B) A micrograph with examples of picked particles, selected two-dimensional class averages, an orientation distribution image, a local resolution graphic, a GSFSC resolution plot, and representative densities for protein and *N*-linked glycan regions are shown for the (A) 1-18–BG505–10-1074 and (B) 1-55–RC1–10-1074 complexes.

**Figure S4:**
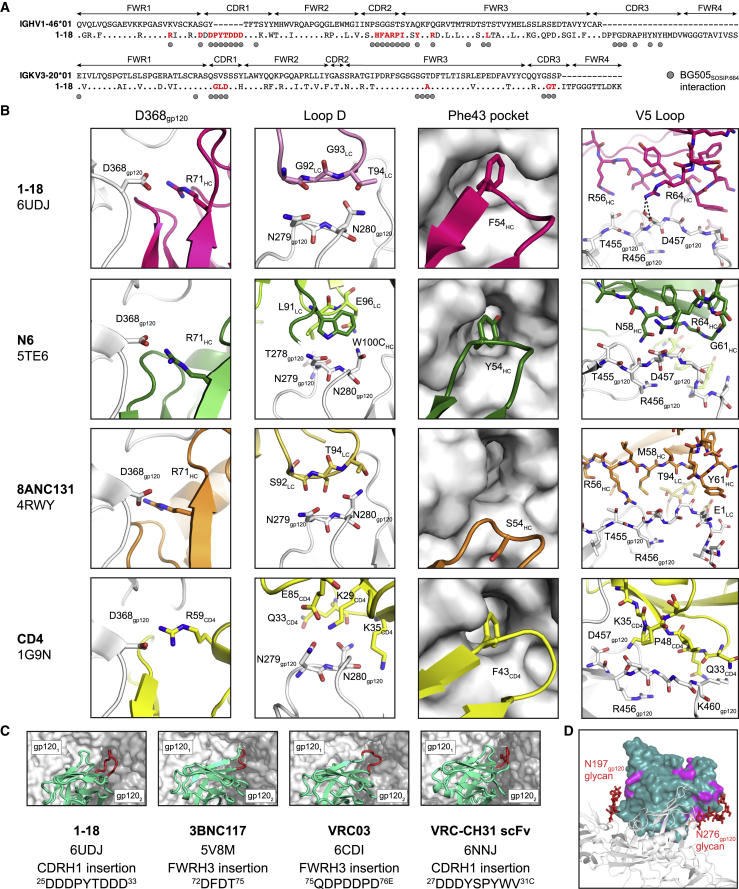
Structural Interaction Details of 1-18- and 1-55-Env Complexes, Related to Figure 3 (A) Alignment of 1-18 heavy (top) and light (bottom) chain sequences to germline. Residues interacting with BG505_SOSIP.664_ are indicated by circles. Interacting residues mutated from the germline sequences are indicated in red. (B) Comparison of Env-interactions of 1-18, the CD4bs bNAbs N6 and 8ANC131, and CD4 at four sites: D368_gp120_, Loop D, the ‘Phe43 pocket’, and the V5 loop. Heavy chains are shown in darker colors than light chains. PDB codes are indicated on the left. (C) Comparison of Env-interactions of bNAbs with Asp-rich insertions in CDRH1 or FWRH3 contacting the adjacent gp120 protomer. Each SOSIP.664 trimer is shown as semi-transparent surface with the primary gp120 protomer in white and the adjacent gp120 in gray. V_H_V_L_ regions are shown in teal with insertions in red. PDB code, insertion location, and insertion sequence are listed. (D) Differences between Fabs of 1-18 and 1-55. One 1-18 V_H_V_L_ (dark green surface) is shown bound to one gp120 (gray cartoon). Locations of residues varying between 1-18 and 1-55 are highlighted in magenta. Glycans at positions N197_gp120_ and N276_gp120_ are shown as red sticks. 1-55 Fabs were based on earlier sequence variants and contained primer-induced mutations at the start and end of the V genes (for a total of 2 aa [V_H__]_ and 4 aa [V_Κ__]_ mutations).

To characterize Env recognition by the 1-18 family of bNAbs, we solved cryo-EM structures of 1-18 and 1-55 Fabs in complex with soluble native-like Env trimers and the V3-targeting bNAb 10-1074 at resolutions of 2.5 Å (1-18 complexed with BG505_SOSIP.664_) and 3.9 Å (1-55 complexed with RC1, a designed immunogen that is a derivative of BG505_SOSIP.664_; Escolano et al., 2019) (Figures 3A and S3; Table S6). Notably, at 2.5-Å resolution, the 1-18 complex is the highest resolution view yet obtained of an HIV-1 Env trimer (Figure S3A; Table S6). Both complexes contained three 1-18 family Fabs and three 10-1074 Fabs interacting with three-fold symmetry with a SOSIP-Env trimer. 1-18 and 1-55 recognized the CD4bs similarly to other V_H_1-46-derived bNAbs, including 8ANC131 and CH235.12 (Bonsignori et al., 2016, Zhou et al., 2015), with interactions encompassing contacts with the N276_gp120_ and N197_gp120_ glycans, the CD4bs loop via the CDRH2, the V5 loop via the CDRH2, and loop D via the CDRL3 (Figures 3A, 3B, S4A, and S4B). However, in addition, 1-18 contacts Env by residue F54_HC_, which is buried in the gp120 ‘Phe43 pocket’, and by residue R64_HC_, which makes a salt bridge with V5 residue D457_gp120_ (Figure S4B). These interactions mimic analogous gp120 contacts made by CD4 residues F43_CD4_ and K35_CD4_, respectively, and the V_H_1-2-derived bNAb N6 also buries an aromatic residue (Y54_HC_) in the ‘Phe43 pocket’ (Huang et al., 2016a, Kwong et al., 1998; Figure S4B).

In addition to the canonical V_H_1-46 contacts, 1-18 contains a six-residue insertion in its CDRH1, resulting in a negatively charged ^25^DDDPYTDDD^33^ motif that interacts with the adjacent gp120 protomer (Figures 3B–3D). At the adjacent protomer, four Asp residues in the 1-18 CDRH1 (D25_HC_, D27_HC_, D31_HC_, and D32_HC_) plus T30_HC_ form coordinated interactions around the highly conserved Env residue K207_gp120_ (Figure 3C). In addition, the increased length of the 1-18 CDRH1 places Y29_HC_ in a position to interact with residues in the V3 loop on the adjacent protomer (S306_gp120_, R308_gp120_, A316_gp120_, and Y318_gp120_) (Figure 3C). Although not all of the Asp residues in the 1-18 CDRH1 contact positively charged residues on gp120, the Asp-rich insertion may have been selected to carry an overall negative charge that is electrostatically attracted to the positively charged patch within the V3 loop on the adjacent protomer (Figure 3D), which could drive formation of an initial Env-antibody complex (Schreiber et al., 1996). Although other CD4bs bNAbs include Asp-containing insertions in either the CDRH1 or heavy-chain framework region 3 (FWRH3) that interact with the positively charged gp120 patch (Lee et al., 2017, Liu et al., 2019, Xu et al., 2018; Figure S4C), the number of Asp in the CDRH1 of 1-18 and the extent of their interaction to Env have not been described before. Notably, gp120 residues contacted by the 1-18 CDRH1 contribute to the CD4 and/or co-receptor binding sites (Liu et al., 2017, Rizzuto et al., 1998, Shaik et al., 2019), and most residues are highly conserved (Figure 3C). Demonstrating their importance for viral function, mutations in some of these residues have been shown to substantially reduce infectivity (de Taeye et al., 2015, Liu et al., 2017).

To evaluate the relevance of the CDRH1 insertion for the neutralizing activity of 1-18, we engineered 1-18Δins, a 1-18 variant lacking the insertion. When tested against the 12-strain global panel, 1-18Δins showed significantly reduced potency compared with 1-18 (GeoMean IC_50_ values of 0.114 μg/mL [1-18Δins] and 0.035 μg/mL [1-18], respectively; p = 0.012; Table S3B). In addition, we investigated antibody 561_02_12 (referred to as 2-12), a member of clone 4 that developed in individual IDC561 but does not have a CDRH1 insertion. Compared with 1-18, antibody 2-12 showed lower breadth on extended pseudovirus panels (119-strain multiclade panel: 1-18, 97% breadth; 2-12, 87% breadth; 100-strain clade C panel: 1-18, 90% breadth; 2-12, 74% breadth; Tables S5A and S5B). Analysis of the neutralization panel data (West et al., 2013) indicated reduced potency of 2-12 against viruses carrying H364_gp120_ in the CD4 binding loop. In contrast, 1-18 is less affected by this variation, suggesting a higher tolerance for structural variations in this site.

Compared with the epitopes of the CD4bs bNAbs 3BNC117 and VRC01 (Lee et al., 2017, Stewart-Jones et al., 2016), 1-18 and 1-55 bury more surface area on both the primary gp120 epitope and the adjacent protomer, another potential mechanism for their increased breadth and potency (Chuang et al., 2019; Figure 3E). Finally, the slightly higher neutralizing activity of 1-18 compared with 1-55 might be explained by variations in glycan accommodation (Figure S4D). We conclude that 1-18 mediates exceptional HIV-1-neutralizing activity by an increase in buried surface on gp120, primarily through increased inter-protomer contacts mediated by its unique CDRH1.

### 1-18 Is Not Affected by Typical VRC01-Class Escape Mutations

**Figure 4:**
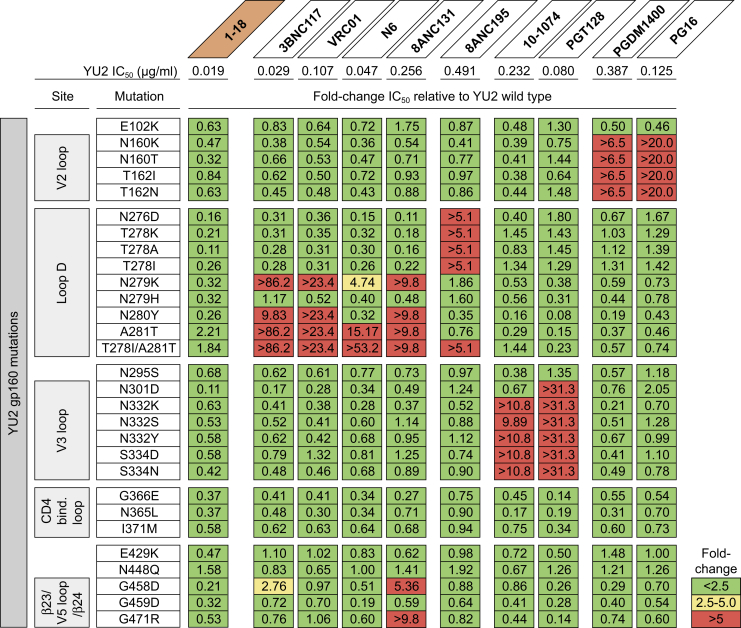
1-18 Overcomes Typical bNAb Escape Mutations *In Vitro* The top row shows bNAb IC_50_ values against the YU2 wild-type pseudovirus. The panels show the change in bNAb sensitivity (fold change of IC_50_) for YU2 pseudovirus mutants compared with the wild type.

To determine how known Env escape mutations affect the neutralizing activity of 1-18, we evaluated the sensitivity of HIV-1_YU2_ pseudovirus variants. As expected, removal of potential *N*-linked glycosylation sites (PNGSs) in the V2 (N160_gp120_) and V3 (N301_gp120_, N332_gp120_) loops mediated resistance to the V1/V2-directed bNAbs PG16 and PDGM1400 and the V3-directed bNAbs 10-1074 and PGT128, respectively (Mouquet et al., 2012, Pejchal et al., 2011, Sok et al., 2014, Walker et al., 2009, Walker et al., 2011), but did not affect neutralization by 1-18 or other CD4bs bNAbs (Figure 4). Similarly, removal of a PNGS adjacent to the CD4bs (N276_gp120_) reduced sensitivity to the gp120-gp41 interface bNAb 8ANC195 (Scharf et al., 2014) but had no effect on 1-18 (Figure 4).

V_H_-restricted CD4bs bNAbs typically interact with loop D residues N279_gp120_ and/or N280_gp120_, and changes in these residues have been associated with viral rebound from CD4bs therapy (Diskin et al., 2013, Horwitz et al., 2013, Julg et al., 2017, Klein et al., 2012, Lynch et al., 2015a). When we tested HIV-1_YU2_ variants with mutations at these residues, we observed reduced or abrogated sensitivity to VRC01-class bNAbs and to the V_H_1-46-derived CD4bs bNAb 8ANC131 (Figure 4). In contrast, these mutations had no or only minimal effects on 1-18 (Figure 4). Maintained neutralizing activity against these variants might be mediated by increased contacts of 1-18’s extended CDRH1 that formed compensatory interactions, alleviating the necessity for loop D contacts normally required by CD4bs antibodies. Additionally, the portion of 1-18’s CDRL3 that contacts loop D utilizes a glycine-rich ^92^GGT^94^ motif rather than the ^92^SST^94^ motif in 8ANC131. This could accommodate mutations in loop D (N279K, N280Y) through increased flexibility. Finally, the 7-Å shift in CDRL2 location between 1-18 and 8ANC131 could allow greater accommodation of a glycan at N279_gp120_ in two HIV-1_YU2_ variants (A281T and T278I/A281T).

Mutations in the β23 and β24 strands surrounding the V5 loop (gp120 residues 451-471) were associated with viral resistance against 8ANC131 but were tolerated by 1-18 (Figure 4). V5 loop residue D457_gp120_ interacts with 1-18 R64_HC_, a somatic mutation from the V_H_1-46 germline that is present in 1-18 but not in 8ANC131. We hypothesize that the R64_HC_-D457_gp120_ salt bridge is a crucial interaction between 1-18 and gp120 that potentially allows it to tolerate common routes of Env escape within the V5 loop.

We conclude that 1-18 maintains full activity against viruses carrying mutations associated with viral resistance against other CD4bs bNAbs *in vitro*.

### Mutational Antigenic Profiling of 1-18 Reveals Restricted HIV-1 Escape

**Figure S5:**
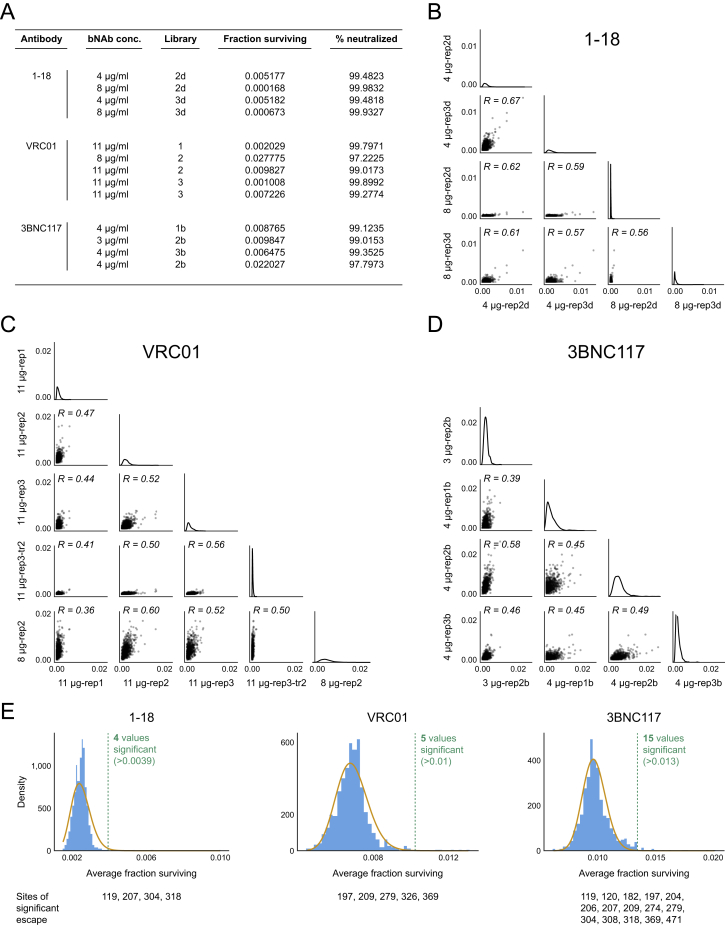
Mutational Antigenic Profiling: Fractions Surviving, Correlation between Replicates, and Determination of Sites of Significant Escape, Related to Figure 5 (A) Antibody concentration during selection, batch of mutant virus library, and fraction of library surviving antibody selection for each biological replicate. (B–D) Correlation between biological replicate selections of average excess fraction surviving at each site in the presence of (B) 1-18, (C) VRC01, and (D) 3BNC117. (E) Distribution of average fraction surviving at each site for each antibody (blue bars). The yellow line overlays the gamma distribution fit using robust regression to site fraction surviving data. Dotted lines mark sites that fall beyond this distribution at a false discovery rate of 0.01. Number of sites beyond this cutoff is labeled in green and individual sites are listed at the bottom. Data for 3BNC117 and VRC01 are from Dingens et al. (2019).

To identify potential pathways of viral escape from 1-18, we used mutational antigenic profiling with libraries of HIV-1_BG505_ variants containing all single amino acid substitutions within the ecto- and transmembrane domains of Env (Figure S5; Dingens et al., 2017, Haddox et al., 2018). In this assay, the effects of Env mutations on antibody resistance are quantitatively determined by deep sequencing of cells that become infected in the presence versus the absence of an antibody.

**Figure 5:**
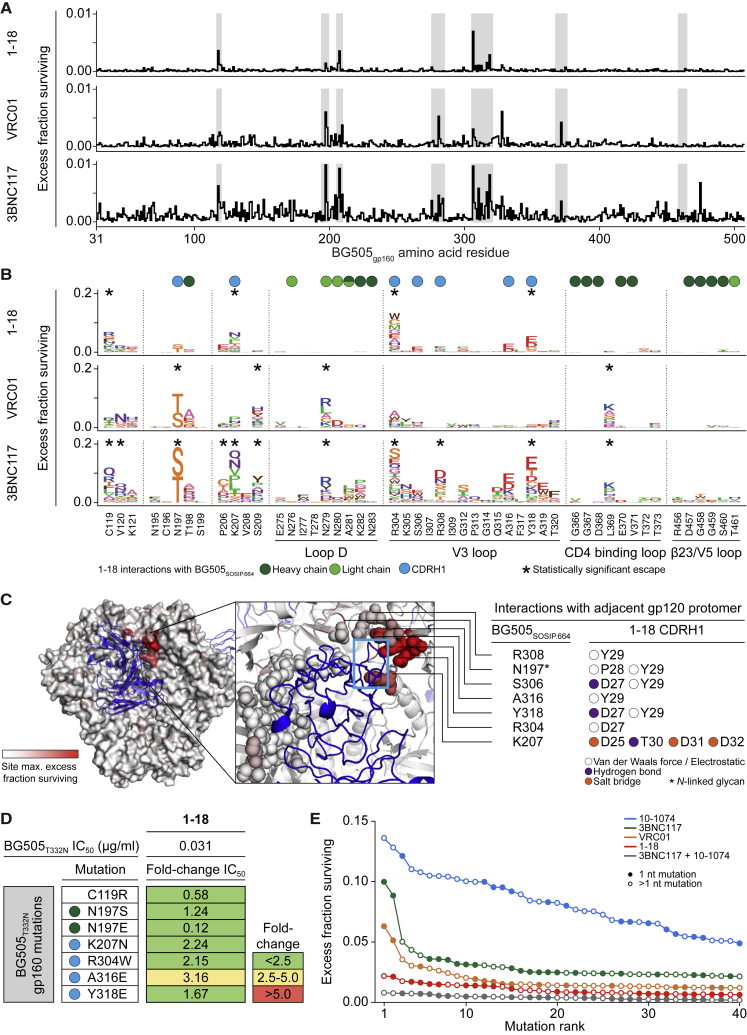
Restricted Pathways of Escape from 1-18 Identified by Mutational Antigenic Profiling (A) Line plots indicate the HIV-1_BG505_ library excess fraction surviving antibody neutralization, averaged across all mutations at each site. Data for antibodies other than 1-18 in all panels are from Dingens et al. (2019). Regions in gray are detailed in (B). (B) HIV-1_BG505_ escape profiles, with letter heights indicating the excess fraction surviving for each mutation. Circles indicate HIV-1_BG505_ residues interacting with 1-18 (cryo-EM). Asterisks indicate residues with statistically significant antibody escape. (C) The BG505_SOSIP.664_ trimer, colored according to the maximum excess fraction surviving 1-18 at each site, with 1-18 shown in blue. In the inset, structurally defined contacts are shown as spheres, and the CDRH1 is highlighted by the rectangle. Interactions of the CDRH1 with the adjacent gp120 protomer are indicated on the right. (D) The top row indicates IC_50_ of 1-18 against the BG505_T332N_ pseudovirus, and the panels show fold change in IC_50_ for BG505_T332N_ pseudovirus variants with mutations in the six residues showing the highest excess fraction surviving 1-18 neutralization. Circles indicate interactions as in (B). (E) Excess fraction surviving for the 40 mutations with the largest effect sizes for each antibody. Circles indicate the number of nucleotide changes required for the respective amino acid mutation. See also Figure S5.

1-18 only selected escape mutants at a small number of residues, all of which were outside of the canonical CD4bs. In contrast to 3BNC117 and VRC01 (Dingens et al., 2019), we observed no statistically significant escape from 1-18 in loop D and the CD4 binding loop (Figures 5A–5C and S5). Rather, 1-18-mediated selection was localized to the V3 loop and the stem of the V1/V2 loop of gp120 (Figure 5C). Among the four sites of significant escape, three residues (K207_gp120_, R304_gp120_, and Y318_gp120_) interact with the CDRH1 of 1-18 (Figure 5C). The fourth residue, C119_gp120_, generally forms a disulfide bond with C205_gp120_ at the stem of the V1/V2 loop. Thus, mutations at residue C119_gp120_ may reduce 1-18 accessibility to the highly conserved K207_gp120_ by disordering the V1/V2 loop structure (Leonard et al., 1990, van Anken et al., 2008).

Mutational antigenic profiling allows identification of the strongest escape mutations for each antibody (Figures 5A–5C and S5E). Importantly, although VRC01 escape mutations were associated with a 3- to more than 175-fold increase in the antibody IC_50_ values for HIV-1_BG505_ pseudovirus variants (Dingens et al., 2019), the effects were much less pronounced for potential 1-18 escape mutants (Figure 5D). When we evaluated HIV-1_BG505_ pseudoviruses carrying single mutations at the six residues with the highest level of 1-18-mediated selection, the sensitivity to 1-18 was reduced by less than 2.3-fold for 5 of the 6 tested viruses (Figure 5D). The sixth virus, carrying an A316E mutation, showed a 3.2-fold decrease in sensitivity (IC_50_ increased to 0.098 μg/mL) (Figure 5D). Therefore, all tested potential escape variants remained highly 1-18-sensitive when evaluated as pseudoviruses. To determine the ease of viral escape, we compared the effects of the 40 strongest mutations from antigenic profiling of 1-18 with those of VRC01, 3BNC117, 10-1074, or the combination of 3BNC117 and 10-1074 (Dingens et al., 2019). The levels of escape observed for 1-18 were lower than those for the single bNAbs and similar to the combination of 3BNC117 and 10-1074 (Figure 5E).

Overall, mutational antigenic profiling of 1-18 revealed a strong limitation of HIV-1_BG505_ escape via single amino acid mutations, with no evidence of selection at the canonical CD4bs that is critical for resistance against VRC01-class bNAbs.

### Full Suppression of Viremia by 1-18 Monotherapy *In Vivo*

**Figure S6:**
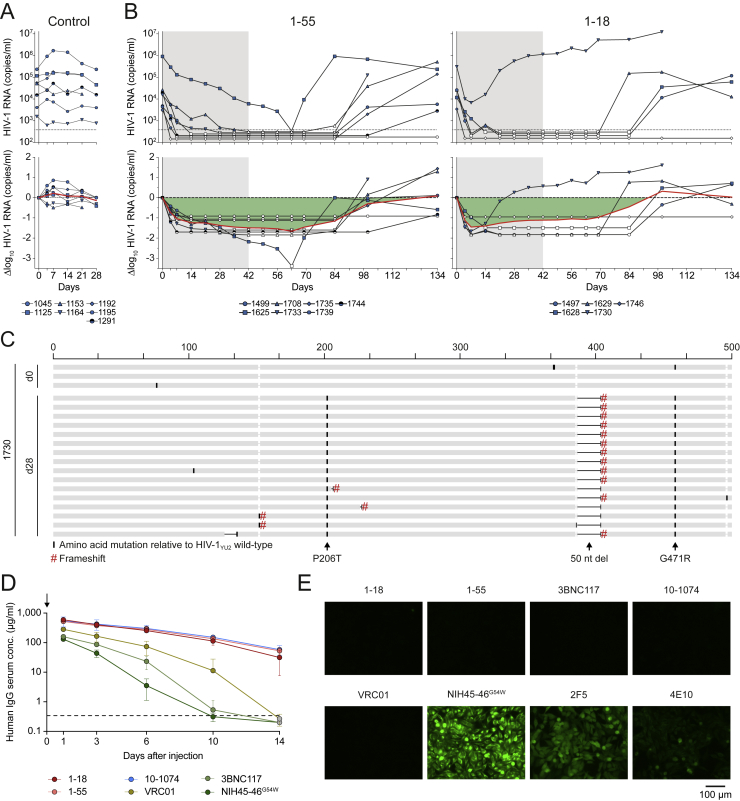
1-18 and 1-55 Antiviral Activity in HIV-1_YU2_-Infected Humanized Mice, Pharmacokinetics, and Autoreactive Properties, Related to Figures 6 and 7 (A) Absolute HIV-1 RNA copies (top) and log_10_ viral load changes (bottom) in untreated HIV-1_YU2_-infected humanized mice. Red line shows average log_10_ viral load change compared to baseline. Dashed line in top panel indicates quantitation limit of accuracy (384 copies/ml). (B) Absolute HIV-1 RNA copies (top) and log_10_ viral load changes (bottom) in HIV-1_YU2_-infected humanized mice treated with 1-55 (left) or 1-18 (right). Grey shading indicates duration of bNAb therapy. Dashed lines in top panels indicate quantitation limit of accuracy (384 copies/ml). Data points in white indicate viral loads < 384 copies/ml. Red lines show average log_10_ viral load change compared to baseline. (C) Alignment of plasma SGS-derived *env* sequences from mouse 1730 obtained on day 0 (top) and day 28 (bottom) based on nucleotide sequences. Indicated changes are amino acid mutations (black bars), mutations resulting in frameshifts (red hash), and nucleotide deletions (black horizontal lines) compared to YU2 wild-type sequence. Amino acid numbering on top is based on HIV-1_YU2_, and indicated mutations are numbered based on HIV-1_HXB2_. (D) Serum human IgG levels in NRG mice after intravenous injection of 0.5 mg of antibody on day 0 (left). Data are represented as mean ± standard deviation, respectively. (E) HEp-2 cell reactivity using the indicated monoclonal antibodies at a concentration of 100 μg/ml.

To determine the antiviral activity of 1-18 *in vivo*, we used HIV-1_YU2_-infected humanized mice that can maintain stable viremia with a rate of HIV-1 diversification similar to what is observed in humans (Gruell and Klein, 2017, Ince et al., 2010, Klein et al., 2012, Zhang et al., 2002; Figure S6A).

**Figure 6:**
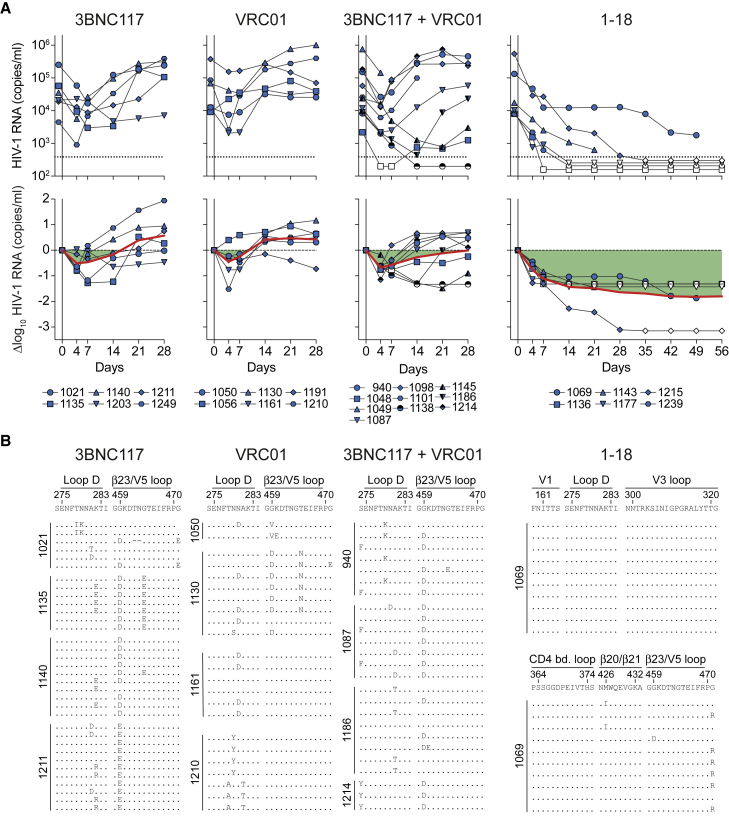
Full Suppression of Viremia by 1-18 Monotherapy *In Vivo* (A) HIV-1 RNA plasma copies (top) and log_10_ viral load changes compared with baseline (day -1) (bottom). Dashed lines in the top panels indicate the quantitation limit of accuracy (384 copies/mL), and data points in white indicate viral loads of less than 384 copies/mL. Red lines show average log_10_ viral load changes compared with baseline. (B) *Env* sequences obtained from day 28 plasma RNA of indicated mice by SGS. Letters indicate amino acid mutations compared with wild-type YU2 shown on top. Residues are numbered according to HIV-1_HXB2_. See also Figure S6 and Table S7.

**Figure 7:**
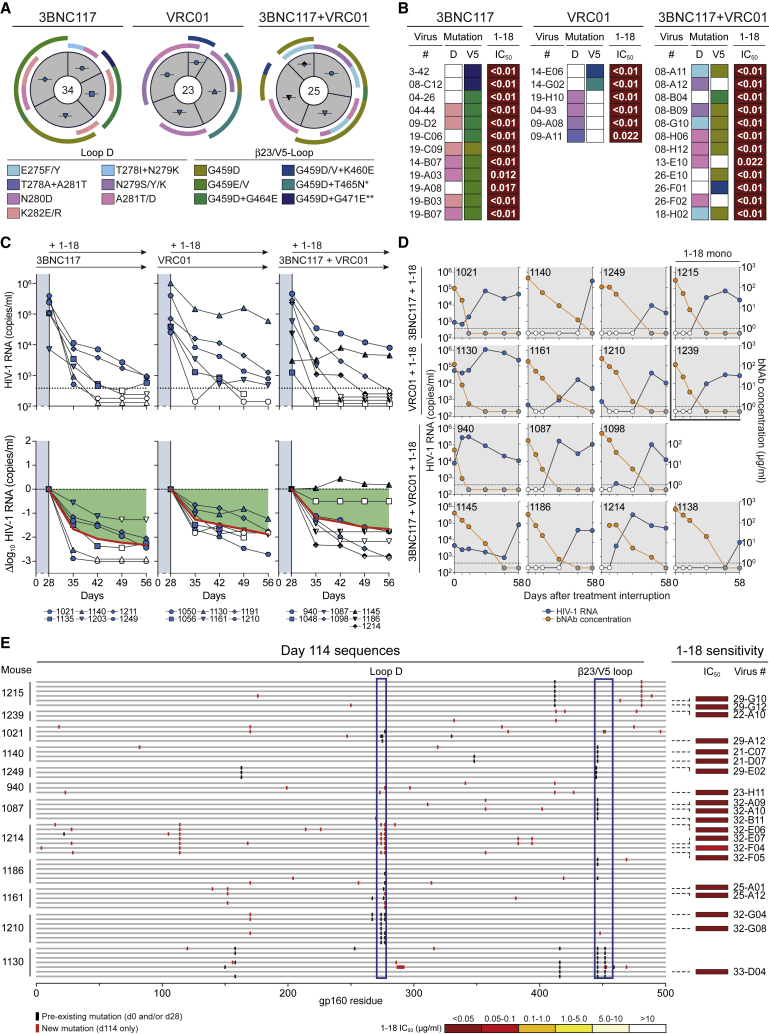
bNAb 1-18 Overcomes VRC01-Class Escape *In Vivo* (A) Pie charts indicate the number of plasma *env* sequences obtained from HIV-1_YU2_-infected humanized mice on day 28 of treatment with 3BNC117, VRC01, or 3BNC117+VRC01. Icons are as in Figure 6A. Outer bars indicate mutations in loop D and/or the β23 strand/V5 loop. ^∗^+ G471E mutation. ^∗∗^+ ΔT462 & ΔN463 mutations. (B) IC_50_s of 1-18 SGS-derived day 28 *env* sequence pseudoviruses with mutations as indicated in (A). (C) Addition of 1-18 treatment on day 28 to HIV-1_YU2_-infected humanized mice that showed viral rebound during 3BNC117, VRC01, or 3BNC117+VRC01 therapy (Figure 6A). The previous treatment regimen was continued. HIV-1 RNA plasma copies are shown at the top and log_10_ viral load changes compared with baseline (day 28) at the bottom. Dashed lines in the top panels indicate the quantitation limit of accuracy (384 copies/mL), and data points in white indicate viral loads of less than 384 copies/mL. Red lines show average log_10_ viral load changes compared with baseline (day 28). (D) HIV-1 RNA plasma copies (left y axis) and plasma bNAb levels determined by BG505_SOSIP.664_-ELISA (right y axis) after interruption of bNAb therapy on day 56 as indicated in (C). Only mice that could be followed for 58 days are included. Dashed lines indicate the HIV-1 RNA quantitation limit of accuracy (384 copies/mL). White circles show viral loads of less than 384 copies/mL, and gray circles indicate antibody levels of less than 1 μg/mL. (E) Plasma SGS-derived *env* sequences obtained on day 114. Black bars indicate amino acid mutations compared with the YU2 wild-type found previously; red bars indicate mutations only found on day 114 within individual mice. Numbering is according to HIV-1_YU2_. Boxes indicate IC_50_ values of 1-18 against the corresponding pseudoviruses (right). See also Table S7.

Following a 1-mg loading dose of antibody administered subcutaneously (s.c.), we treated HIV-1_YU2_-infected mice (n = 6–10 per group) twice a week with s.c. injections of 0.5 mg per bNAb for 4–8 weeks (Figure 6A). Treatment with 3BNC117, VRC01, or the combination of both bNAbs resulted in mean viral load reductions of 0.5, 0.5, and 0.7 log_10_ copies/mL, respectively. However, these effects were only transient, and viral rebound occurred within the first 2 weeks in most animals, indicating rapid viral escape (Figure 6A). Indeed, when plasma single-genome sequencing (SGS) (Salazar-Gonzalez et al., 2008) was performed at week 4 after the start of treatment, 79 of 82 isolated viruses from 16 mice showed mutations in the VRC01 and 3BNC117 target sites in loop D and/or the β23/V5 loop regions of gp120 (Figures 6B and 7A; Tables S7A–S7C).

In contrast to the transient effects of 3BNC117 and VRC01, monotherapy of HIV-1_YU2_-infected humanized mice with 1-18 resulted in sustained viral suppression over a period of 8 weeks in all treated animals (average drop of 1.7 log_10_ copies/mL; Figure 6A). Moreover, in all but one mouse, viremia dropped to levels below the level of quantitation (384 copies/mL) by day 35. From the one mouse (1069) that had quantifiable levels of viremia but was suppressed (drop of 1.9 log_10_ copies/mL), we analyzed HIV-1 *env* sequences at week 4 (Figures 6A and 6B). We detected no recurrent mutations at contact residues of 1-18 or typical CD4bs bNAbs, and pseudoviruses derived from these sequences remained sensitive to 1-18 (Figure 6B; Table S7D). These results were confirmed in an independent repeat experiment for 1-18 as well as for 1-55, another member of clone 4.1 (Figure S6B). Although five of six 1-18-treated mice in this experiment were fully suppressed, one mouse (1730) continued to show high HIV-1 RNA copy numbers (Figure S6B). However, all *env* sequences obtained from this mouse carried large V4 loop deletions as well as early stop codons (Figure S6C). Thus, despite detectable rebound of viremia in 1 of 18 mice treated with 1-18 or 1-55, no functional escape viruses were observed by SGS and evaluation of pseudoviruses.

Effective clinical application of bNAbs depends on favorable safety and pharmacokinetic profiles. Following a single injection of 1-18 or 1-55, their decline in serum of immunodeficient mice was slower compared with VRC01-class bNAbs and more similar to 10-1074, which has a longer half-life than 3BNC117 and VRC01 in humans (Bar-On et al., 2018, Caskey et al., 2015, Caskey et al., 2017, Ledgerwood et al., 2015, Lynch et al., 2015a, Mendoza et al., 2018; Figure S6D). In addition, whereas some bNAbs demonstrate binding to self-antigens (Haynes et al., 2005), we found no indication for autoreactivity of 1-18 or 1-55 when tested against HEp-2 cells (Figure S6E).

In summary, we conclude that 1-18 has exceptional antiviral *in vivo* activity against HIV-1_YU2_. This activity is superior to the CD4bs antibodies 3BNC117 and VRC01, which are currently being evaluated in clinical trials. Importantly, single bNAb therapy with 1-18 is sufficient to effectively prevent development of viral escape variants that rapidly emerge during HIV-1_YU2_ monotherapy with other bNAbs (Horwitz et al., 2013, Klein et al., 2012).

### 1-18 Overcomes VRC01-Class Escape Mutations *In Vivo*

To confirm that mutations occurring during 3BNC117 and/or VRC01 therapy conferred antibody resistance, we generated 30 pseudoviruses derived from day 28 *env* sequences of 11 VRC01-class-treated mice. We found 23 viral variants that were fully resistant to the administered antibodies (IC_50_ > 25 μg/mL) or showed increased VRC01-class resistance (>5-fold increase in IC_50_ values). Notably, however, all of these pseudoviruses remained sensitive to 1-18 *in vitro* (Figure 7B; Table S7).

To determine whether 1-18 can overcome escape from VRC01-class bNAbs *in vivo*, we added bNAb 1-18 therapy (1-mg loading dose s.c. followed by 0.5 mg s.c. twice weekly) to 3BNC117- and/or VRC01-pretreated animals while continuing 3BNC117 and/or VRC01 administrations (Figure 7C). Despite circulating VRC01-class-resistant viral variants, 1-18 effectively reduced viremia and maintained viral suppression in 17 of 18 mice (Figure 7C). Following interruption of bNAb therapy, viral rebound occurred in all fully suppressed mice when Env-reactive antibody plasma concentrations declined to a median of 0.1 μg/mL (Figure 7D). To determine whether declining 1-18 levels resulted in selection of 1-18-resistant escape variants, we performed plasma SGS of rebound viruses 8 weeks after treatment interruption (day 114) (Figure 7E). Although we found novel mutations compared with day 0 and day 28 in 39 of 60 sequences from 12 mice, there was no recurrent pattern of mutations that developed after 1-18 therapy (Figure 7E). Indeed, all 20 sequences tested as pseudoviruses were demonstrated to be fully sensitive to 1-18 (Figure 7E; Table S7).

We conclude that bNAb 1-18 effectively overcomes VRC01-class resistance *in vivo* and maintains viral suppression without the development of 1-18-resistant HIV-1_YU2_ variants.

## Discussion

Implementation of HIV-1-neutralizing antibodies for clinical practice requires antibodies with specific characteristics. These include safety, a favorable pharmacokinetic profile, and broad and highly potent neutralizing activity to effectively target the remarkable diversity of HIV-1 (Caskey et al., 2019). In addition, as for any drug against HIV-1, viral escape represents one of the biggest challenges for clinical application. This became evident when (1) single bNAbs were used for therapy and HIV-1 resistance developed within a few weeks (Bar et al., 2016, Caskey et al., 2015, Caskey et al., 2017, Klein et al., 2012, Lynch et al., 2015a, Scheid et al., 2016), and (2) antibody combinations resulted in improved viral control by preventing early development of resistance (Bar-On et al., 2018, Klein et al., 2012, Mendoza et al., 2018). Therefore, restriction of HIV-1 escape will be an antibody-dependent feature of utmost importance for successful bNAb applications.

VRC01-class antibodies targeting the functionally critical CD4bs have broad and potent neutralizing activity but fail to prevent viral escape *in vivo* (Bar et al., 2016, Caskey et al., 2015, Horwitz et al., 2013, Klein et al., 2012, Lynch et al., 2015a, Scheid et al., 2016). Through a combination of functional *in vitro* mapping and *in vivo* therapy of HIV-1-infected humanized mice, we demonstrated that the V_H_1-46-derived CD4bs bNAb 1-18 effectively restricts development of HIV-1 resistance. Contrasting other CD4bs bNAbs, we did not identify single amino acid mutations resulting in 1-18 resistance in two viral strains of different clades (BG505, clade A; YU2, clade B). Most importantly, in the setting of viral replication and diversification in HIV-1_YU2_-infected humanized mice, 1-18 monotherapy resulted in effective and sustained viral suppression. Notably, the *in vivo* activity of 1-18 was not affected by VRC01-class-resistant viral variants. Thus, despite mutations in up to two sites associated with escape from CD4bs antibodies, 1-18 effectively suppressed viremia and restricted the development of additional escape mutations.

In our cryo-EM analysis of a 1-18-BG505 complex, the highest-resolution HIV-1 Env trimer structure obtained to date (2.5 Å), the details of 1-18 recognition of Env were determined. The structure demonstrated that 1-18 combines favorable features found in potent V_H_1-2 bNAbs that likely contribute to its exceptional potency and breadth. These characteristics include (1) an aromatic residue that mimics residue Phe43 of CD4 to target the ‘Phe43_gp120_ pocket’, as seen in bNAb N6 (Huang et al., 2016a); (2) contacts with the adjacent gp120 protomer, as seen for bNAb 3BNC117 (Lee et al., 2017, Lyumkis et al., 2013) but with increased buried surface area (via its six-residue insertion in CDRH1); and (3) a larger buried surface area on gp120 than other V_H_1-2 bNAbs: 1,530 Å^2^ compared with 1,260 Å^2^ (VRC01) and 1,290 Å^2^ (3BNC117). Finally, 1-18’s unique mode of binding enables it to make additional contacts with conserved residues on gp120 not found in other CD4bs bNAbs, allowing 1-18 to rely less on classical CD4bs bNAb contacts and making viral escape more difficult. These characteristics may explain 1-18’s exceptional potency, breadth, and resistance to viral escape. Notably, functional antigenic mapping demonstrated that 1-18-mediated selection was focused on contact residues within the CDRH1. Moreover, the lack of the CDRH1 insertion in antibodies 1-18Δins and 2-12 was associated with reduced neutralizing activity compared with 1-18.

**Figure S7:**
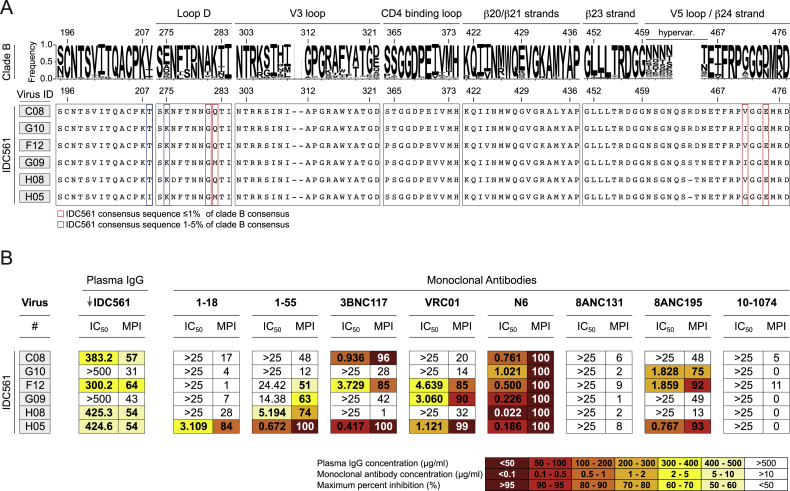
Plasma SGS-Derived *env* Sequences Obtained from Donor IDC561, Related to Figures 1 and S1 (A) Letter heights indicate amino acid frequency among 2,351 clade B sequences obtained from the Los Alamos National Laboratory (LANL) database (top). Bottom panels show selected sites of plasma SGS-derived *env* sequences obtained from IDC561 from the leukapheresis sample from which 1-18 and 1-55 were isolated. Boxes indicate amino acids for which IDC561 consensus sequence is represented in ≤ 1% (red) or 1%–5% (blue) of the LANL clade B sequences. Numbering relative to HIV-1_HXB2_ reference strain. (B) Neutralization sensitivity of pseudoviruses based on IDC561 sequences indicated in (A). Maximum percent inhibition (MPI) determined when tested at maximum concentrations of 500 μg/ml (purified IgG) or 25 μg/ml (monoclonal antibodies). Plasma IgG was obtained at the time of leukapheresis from which 1-18 was isolated.

Despite the remarkable neutralization breadth of 1-18 (covering 256 of 271 evaluated primary viruses and pseudoviruses), a small number of viruses was found to be 1-18 resistant. However, sequence analysis (West et al., 2013) did not identify single residues that were associated with 1-18 resistance. In addition, we analyzed viruses from individual IDC561, from whom 1-18 was identified. Although viremia was controlled in the absence of ART for more than 15 years, it was detectable by the time of 1-18 isolation. Indeed, viruses obtained at this time showed resistance against 1-18 and clonal members (Figure S7). Remarkably, however, their CD4bs sequences presented with highly infrequent amino acid residues (Figure S7). For example, among 2,351 clade B *env* sequences in the Los Alamos HIV Sequence Database, only 0.8% carried a glycine at the loop D residue 281_gp120_, and not a single virus had a glutamic acid at position 474_gp120_ (Figure S7). Similarly, uncommon amino acids were found in other loop D (282_gp120_) and V5 loop (471_gp120_) positions as well as at residue 208_gp120_, which neighbors K207_gp120_ that strongly interacts with the CDRH1 of 1-18 (Figure S7). Taking the results from profiling escape pathways *in vitro* together with the lack of viral escape in humanized mouse experiments, the occurrence of multiple uncommon amino acid residues in 1-18-resistant viruses suggests a restricted escape pathway from 1-18 that may require accumulation of multiple rare mutations.

Current strategies to combine bNAbs are based on the use of antibodies targeting non-overlapping epitopes (Bar-On et al., 2018, Barouch et al., 2013, Huang et al., 2016b, Klein et al., 2012, Mendoza et al., 2018, Shingai et al., 2013, Xu et al., 2017). To this end, the combination of 1-18 with bNAbs targeting other epitopes (e.g., the MPER antibody DH511.2_K3, which neutralizes 100% of tested clade C viruses; Williams et al., 2017) may be a promising option. Because the most potent known CD4bs bNAbs are of the VRC01-class, synergistic effects by combinations of these antibodies are not expected. However, given their different binding, neutralization, and escape patterns, a combination of 1-18 with VRC01-class CD4bs bNAbs may be highly beneficial. The capacity of 1-18 to overcome VRC01-class escape mutations *in vivo* as well as a calculated breadth of more than 99% when 1-18 is combined with VRC01-class bNAbs (e.g., N6 or 3BNC117) offers new possibilities of bNAb combinations, such as a dual anti-CD4bs therapy. Finally, CD4bs bNAbs have been demonstrated to induce escape variants with reduced viral fitness (Lynch et al., 2015b, Otsuka et al., 2018, Sather et al., 2012). Applying double CD4bs-targeting pressure may force the virus to more extensively mutate this functionally critical epitope and, therefore, result in impaired viral variants and prolonged viral control.

In summary, by combining outstanding neutralizing activity and a high barrier for viral escape, 1-18 provides a new option for highly effective treatment and prevention of HIV-1 infection.

## STAR★Methods

### Key Resources Table

**Table:** 

REAGENT or RESOURCE	SOURCE	IDENTIFIER
**Antibodies**		

Anti-Human CD19-Alexa Fluor 700 (Clone HIB19)	BD Biosciences	Cat#557921; RRID: AB_396942
Anti-Human IgG-APC (Clone G18-145)	BD Biosciences	Cat#550931; RRID: AB_398478
Anti-Human CD20-Alexa Fluor 700 (Clone 2H7)	BD Biosciences	Cat#560631; RRID: AB_1727447
Anti-Human IgD-Pe-Cy7 (Clone IA6-2)	BD Biosciences	Cat#561314; RRID: AB_10642457
Anti-Human IgM-FITC (Clone G20-127)	BD Biosciences	Cat#555782; RRID: AB_396117
Anti-Human CD27-PE (Clone M-T271)	BD Biosciences	Cat#560985; RRID: AB_10563213
Peroxidase AffiniPure Goat Anti-Human IgG, Fcγ fragment specific	Jackson ImmunoResearch	Cat#109-035-098; RRID: AB_2337586
AffiniPure Goat Anti-Human IgG, Fcγ fragment specific	Jackson ImmunoResearch	Cat#109-005-098; RRID: AB_2337541
Anti-6X His tag antibody	Abcam	Cat#ab9108; RRID: AB_307016
IgG1, Kappa from human myeloma plasma	Sigma-Aldrich	Cat#I5154; RRID: AB_1163610
Monoclonal anti-HIV-1 Env 3BNC117	NIH Aids Reagent Program	Cat#12474; RRID: AB_2491033
Monoclonal anti-HIV-1 Env VRC01	NIH Aids Reagent Program	Cat#12033; RRID: AB_2491019
Monoclonal anti-HIV-1 Env N6	NIH Aids Reagent Program	Cat#12968
Monoclonal anti-HIV-1 Env NIH45-46^G54W^	NIH Aids Reagent Program	Cat#12174
Monoclonal anti-HIV-1 Env 2F5	NIH Aids Reagent Program	Cat#1475; RRID: AB_2491015
Monoclonal anti-HIV-1 Env 4E10	NIH Aids Reagent Program	Cat#10091; RRID: AB_2491029
Monoclonal anti-HIV-1 Env 10-1074	NIH Aids Reagent Program	Cat#12477; RRID: AB_2491062
Monoclonal anti-HIV-1 Env PGDM14000	Sok et al., 2014	N/A
Monoclonal anti-HIV-1 Env 8ANC131	Scheid et al., 2011	N/A
Monoclonal anti-HIV-1 Env 8ANC195	Scheid et al., 2011	RRID: AB_2491037
Monoclonal anti-HIV-1 Env PGT128	NIH Aids Reagent Program	Cat#13352; RRID: AB_2491047
Monoclonal anti-HIV-1 Env 561_01_18	This paper	N/A
Monoclonal anti-HIV-1 Env 561_01_55	This paper	N/A
Monoclonal anti-HIV-1 Env 561_02_12	This paper	N/A
Monoclonal anti-HIV-1 Env 1-18Δins	This paper	N/A
Monoclonal anti-HIV-1 Env Patient IDC561-derived antibodies	This paper	N/A

**Bacterial and Virus Strains**		

Global Panel: 12 HIV-1 Env-pseudotyped viruses	NIH Aids Reagent Program	Cat#12670
f61 Panel: 20 HIV-1 Env-pseudotyped viruses	Doria-Rose et al., 2017	N/A
Cross Clade Panel: 119 HIV-1 Env-pseudotyped viruses	M.S. Seaman, BIDMC; Seaman et al., 2010	N/A
Clade C Panel: 100 HIV-1 Env-pseudotyped viruses	Hraber et al., 2017	N/A
Replication-competent HIV-1_YU2_ (YU2 *env* in pNL4-3)	P.D. Bieniasz, The Rockefeller University; Zhang et al., 2002	N/A
YU2-pseudotyped viruses carrying mutations in common anti-HIV-1-mAb binding sites	Klein et al., 2012	N/A
BG505.T332N-pseudotyped viruses carrying mutations in 1-18-selected residues	This paper	N/A

**Biological Samples**		

PBMCs of donor IDC561	This paper	N/A
IgGs of donor IDC561	This paper	N/A
Plasma of donor IDC561	This paper	N/A
Viral outgrowth culture of donor IDC561	This paper	N/A
Placental tissue and human cord blood for isolation of human CD34+ cells	This paper	N/A

**Chemicals, Peptides, and Recombinant Proteins**		

DMSO	Sigma-Aldrich	Cat#D2650; CAS: 67-68-5
DAPI	Thermo Fisher	Cat#D1306; CAS: 581-88-4
RC1	Escolano et al., 2019	N/A
BG505_SOSIP.664_	J.P. Moore, Weill Cornell Medical College; Sanders et al., 2013	N/A
BG505_SOSIP.664_-GFP	Sliepen et al., 2015	N/A
BG505_SOSIP.664_-His	J.P. Moore, Weill Cornell Medical College; Sanders et al., 2013	N/A
YU2_gp140_ (fold-on trimer)	R. Wyatt, The Scripps Research Institute; Yang et al., 2000	N/A
YU2_gp120_	J. Sodroski, Dana Farber Cancer Institute	N/A
BAL_gp140_	Pietzsch et al., 2010	N/A
93THO57_gp120_	Anderson et al., 2000	N/A
RSC3	N.A. Doria-Rose, VRC; Wu et al., 2010	N/A
eOD-GT8	L. Stamatatos, Fred Hutch; Dosenovic et al., 2019	N/A
Streptavidin PE	BD Biosciences	Cat#349023
DTT	Promega	Cat#P1171
RNasin	Promega	Cat#N2515
RNaseOUT	Thermo Fisher	Cat#10777019
SuperScript III Reverse Transcriptase	Thermo Fisher	Cat#18080044
SuperScript IV Reverse Transcriptase	Thermo Fisher	Cat#18090050
Platinum Taq DNA Polymerase	Thermo Fisher	Cat#10966034
Q5 Hot Start High Fidelity DNA Polymerase	NEB	Cat#M0493L
Branched Polyethylenimine, 25 kDa	Sigma-Aldrich	Cat#408727; CAS: 9002-98-6
FreeStyle Expression Medium	Thermo Fisher	Cat#12338001
Protein G Sepharose 4 Fast Flow	GE Life Sciences	Cat#17061805
HiTrap MabSelect Protein A column	GE Life Sciences	Cat#8408255
ABTS solution	Thermo Fisher	Cat#002024
Peroxidase streptavidin	Jackson ImmunoResearch	Cat#016-030-084
Platinum Taq Green Hot Start DNA Polymerase	Thermo Fisher	Cat#11966034
KAPA HiFI HotStart ReadyMix (2X)	Roche	Cat#KK2602
RNase-free DNase I	QIAGEN	Cat#79254
RNase H	Thermo Fisher	Cat#18021071
Phusion Hot Start Flex DNA Polymerase	New England Biolabs	Cat#M0535L
Phytohemagglutinin PHA-M	Sigma-Aldrich	Cat#L8902
Human IL-2, premium grade	Miltenyi Biotec	Cat#130-097-746
Polybrene	Sigma-Aldrich	Cat#H9268; CAS: 28728-55-4
FuGENE 6 Transfection Reagent	Promega	Cat#E2691
Taqman RNA-to-Ct 1-Step Kit	Thermo Fisher	Cat#4392938
Triton X-100	Carl Roth	Cat#3051.4; CAS: 9036-19-5
Dulbecco’s Modified Eagle Medium (DMEM)	Thermo Fisher	Cat#11960-044
Fetal bovine serum (FBS)	Sigma-Aldrich	Cat#F9665
Sodium Pyruvate	Thermo Fisher	Cat#11360-070
L-Glutamine	Thermo Fisher	Cat#25030024
Gentamicin	Sigma-Aldrich	Cat#G1397; CAS: 1405-41-0
ProCHO-5 media	Lonza	Cat#12-766Q
HEPES	Thermo Fisher	Cat#15630-080
GlutaMAX	Thermo Fisher	Cat#35050-061
MEM NEAA	Thermo Fisher	Cat#11140-050
Hygromycin B	Thermo Fisher	Cat#10687-010
Bright-Glo Luciferase Assay System	Promega	Cat#E2650
D-Luciferin, Sodium Salt	GoldBio	Cat#LUCNA-1G; CAS: 103404-75-7
IGEPAL	Sigma-Aldrich	Cat#I8896; CAS: 9002-93-1
ATP	Sigma-Aldrich	Cat#A26209; CAS: 34369-07-8
MgCl_2_	Sigma-Aldrich	Cat#M8266; CAS: 7786-30-3
Coenzyme A sodium salt hydrate	Sigma-Aldrich	Cat#C3144; CAS: 55672-92-9
T4 DNA Polymerase	New England Biolabs	Cat#M0203L
Platinum Taq Green Hot Start	Thermo Fisher	Cat#11966034
Platinum Taq High Fidelity	Thermo Fisher	Cat#11304029
NP-40	Thermo Fisher	Cat#85124
dNTP Mix	Thermo Fisher	Cat#R1122
DTT	Sigma-Aldrich	Cat#GE17-1318-01
Ni-NTA Agarose	Macherey-Nagel	Cat#745400.25
SMARTScribe Reverse Transcriptase	Takara Bio	Cat#639537

**Critical Commercial Assays**		

NOVA Lite HEp-2 ANA Kit	Inova Diagnostics	Cat#708100

**Deposited Data**		

1-18/10-1074/BG505SOSIP.664 coordinates	This paper	PDB: 6UDJ
1-18/10-1074/BG505SOSIP.664 EM map	This paper	EMDB: EMDB-20739
1-55/10-1074/RC1SOSIP.664 coordinates	This paper	PDB: 6UDK
1-55/10-1074/RC1SOSIP.664 EM map	This paper	EMDB: EMDB-20740
Cloned and tested antibodies of IDC561	This paper	GenBank: MN867951 - MN868062
Mouse plasma SGS-derived gp160 sequences	This paper	GenBank: MN870987 - MN871327
IDC 561 plasma SGS-derived gp160 sequences	This paper	GenBank: MN871328 - MN871333
Mutational antigenic profiling analysis of 1-18	This paper	https://github.com/jbloomlab/MAP_118
Mutational antigenic profiling sequencing data of 1-18	This paper	SRA: SRX6752366 - SRX6752371

**Experimental Models: Cell Lines**		

293-6E cells	NRC	NRC file 11565
HEK293T cells	ATCC	Cat#CRL-11268
TZM-bl cells	NIH AIDS Reagent Program; Platt et al., 1998	Cat#8129
SupT1-R5 cells	Didigu et al., 2014	N/A
Expi293 cells	Thermo Fisher	Cat#A14635
Chinese Hamster Ovary (CHO) cells	J.P. Moore and A. Cupo, Weill Cornell Medical College; Chung et al., 2014	N/A

**Experimental Models: Organisms/Strains**		

NOD.Cg-*Rag1*^*tm1mom*^*Il2rg*^*tm1Wjl*^/SzJ (NRG) mice	The Jackson Laboratory	Cat#007799

**Oligonucleotides**		

HIV-1 qPCR primer and probe set	Horwitz et al., 2013	N/A
SGS primer for humanized mice	Klein et al., 2012, Horwitz et al., 2017	N/A
SGS primer for patient samples	Kirchherr et al., 2007, Schoofs et al., 2016	N/A
CG_RT	Ozawa et al., 2006	N/A
IgG_Internal RT	Tiller et al., 2008	N/A
OPT5/oPR-Primer-Mix	Kreer et al., 2019	N/A
Random Hexamer Primer	Thermo Fisher	Cat#SO142

**Recombinant DNA**		

pSG3Δenv	NIH Aids Reagent Program	Cat#11051
YU2 Env expression plasmid	M.S. Seaman, BIDMC	N/A
pBG505.T332N Env expression plasmid	Rogier W. Sanders, Amsterdam Medical Center	N/A
Human antibody expression vectors (IgG1, Igκ, Igλ)	Tiller et al., 2008	N/A

**Software and Algorithms**		

Geneious R10 and Geneious Prime	Geneious	RRID: SCR_010519
Prism	GraphPad	RRID: SCR_002798
Neutralization fingerprinting analysis	Doria-Rose et al., 2017	N/A
IgBlast	National Library of Medicine; Ye et al., 2013	RRID: SCR_002873
Antibody Database v2.0	West et al., 2013	N/A
PyMOL (Version 1.8.2.1)	Schrodinger, LLC	RRID: SCR_000305
UCSF Chimera	Goddard et al., 2007	RRID: SCR_004097
APBS/PDB2PQR	Unni et al., 2011	N/A
PDBePISA	Krissinel and Henrick, 2007	RRID: SCR_015749
Local Res	Kucukelbir et al., 2014	N/A
Coot	Emsley et al., 2010	RRID: SCR_014222
Phenix	Adams et al., 2010	RRID: SCR_014224
MolProbity	Chen et al., 2010	RRID: SCR_014226
Python (Version 3)	Python Software Foundation	RRID: SCR_008394
SciPy	SciPy developers	RRID: SCR_008058
Dms_tools2 (Version 2.5.1)	https://jbloomlab.github.io/dms_tools2/; Bloom, 2015	N/A
HIV Assembler	T.Y. Oliveira, The Rockefeller University; Gaebler et al., 2019	N/A

**Other**		

Pan B Cell Isolation Kit, human	Miltenyi Biotec	Cat#131-101-638
B Cell Isolation Kit II, human	Miltenyi Biotec	Cat#130-091-151
IgG^+^ Memory B Cell Isolation Kit, human	Miltenyi Biotec	Cat#130-094-350
EZ Link Sulfo NHS Biotin and Labeling Kit	Thermo Fisher	Cat#21326
QuikChange II XL Site-Directed Mutagenesis Kit	Agilent	Cat#200521
Q5 Site-Directed Mutagenesis Kit	New England Biolabs	Cat#E0554S
Nextera DNA Library Prep Kit	Illumina	Cat#FC-121-1031
Nextera Index Kit	Illumina	Cat#FC-121-1012
AMPure XP Beads	Beckman Coulter	Cat#A63880
MiSeq Reagent Nano Kit v2 (300-cycles)	Illumina	Cat#MS-103-1002
MiSeq Reagent Kit v3 (600-cycle)	Illumina	Cat#MS-102-3003
MinElute Virus Spin Kit	QIAGEN	Cat#57704
CD4^+^ T Cell Isolation Kit, human	Miltenyi Biotec	Cat#130-096-533
CD8 MicroBeads, human	Miltenyi Biotec	Cat#130-045-201
CD34 MicroBeads, human	Miltenyi Biotec	Cat#130-046-703
CD19 MicroBeads, human	Miltenyi Biotec	Cat#130-050-301
Superdex-200 Increase 10/300 Column	GE Life Sciences	Cat#28990944
HiLoad Superdex-200 16/60 Column	GE Life Sciences	Cat#28989335
IGHV 1-46^∗^1 sequence	GenBank	X92343.1

### Lead Contact and Materials Availability

Further information and requests for resources and reagents should be directed to and will be fulfilled by the Lead Contact, Florian Klein (florian.klein@uk-koeln.de). Nucleotide sequences of all generated antibodies were deposited at GenBank, and expression plasmids for 1-18, 1-55, 2-12, and clonal members will be shared upon request.

### Experimental Model and Subject Details

#### Human Subjects

Blood and leukapheresis samples were obtained under protocols approved by the Institutional Review Board of the University of Cologne (protocols 13-364 and 16-054) and the local IRBs. All participants provided written informed consent. Participants of the neutralization screening cohort are recruited at hospitals and/or private practices in Germany (Cologne, Essen, and Frankfurt), Cameroon (Yaoundé), Nepal (Kathmandu), and Tanzania (Mbeya). At the time of leukapheresis, IDC561 was a 48-year-old male who was diagnosed with HIV-1 infection 21 years earlier. He was recruited at the University Hospital Cologne. PBMCs for bulk viral outgrowth cultures were obtained from individuals recruited in Germany (Cologne).

#### Mouse Models

NOD.Cg-*Rag1*^*tm1mom*^*Il2rg*^*tm1Wjl*^/SzJ (NRG) mice were purchased at The Jackson Laboratory and bred and maintained at the Decentralized Animal Husbandry Network (Dezentrales Tierhaltungsnetzwerk) of the University of Cologne under specific pathogen-free (SPF) conditions with 12-hour light/dark cycles. For breeding purposes, mice were provided with ssniff 1124 breeding feed; for experimental purposes, mice were provided with ssniff 1543 maintenance feed. Humanized mice were generated as previously described with modifications (Klein et al., 2012). In brief, human CD34^+^ hematopoietic stem cells were isolated from cord blood and perfused placental tissues using CD34 microbeads (Miltenyi Biotec). Collection of cord blood and placental tissues was conducted under a protocol approved by the Institutional Review Board of the University of Cologne (16-110), and all donors provided written informed consent. NRG mice were sublethally irradiated within 5 days of birth and intrahepatically injected with purified human CD34^+^ stem cells 4 to 6 hours later. Success of humanization was determined approximately 12 weeks later by FACS analysis of blood for human PBMCs. All mouse experiments were authorized by the State Agency for Nature, Environmental Protection, and Consumer Protection North Rhine-Westphalia (LANUV).

#### Cell Lines

HEK293T cells (American Type Culture Collection) were maintained at 37°C and 5% CO_2_ in Dulbecco’s Modified Eagle Medium (DMEM, Thermo Fisher) supplemented with 10% fetal bovine serum (FBS, Sigma-Aldrich), 1 mM sodium pyruvate, 2 mM L-glutamine, and 1x antibiotic-antimycotic (all from Thermo Fisher). TZM-bl cells (Platt et al., 1998) were maintained at 37°C in 5% CO_2_ in DMEM supplemented with 10% FBS, 1 mM sodium pyruvate, 2 mM L-glutamine, 50 μg/ml gentamicin (Merck), and 25 mM HEPES (Millipore). 293-6E cells (National Research Council of Canada) were maintained at 37°C and 6% CO_2_ in FreeStyle Expression Medium (Thermo Fisher) and kept under constant shaking at 90-120 rpm. Expi293 cells (Thermo Fisher) were maintained at 37°C and 8% CO_2_ in Expi293 Expression medium (Thermo Fisher) and kept under constant shaking at 130 rpm. CHO cells were maintained at 37°C and 5% CO2 in ProCHO-5 media (Lonza) supplemented with 0.1 M HEPES, 1x GlutaMAX, 1x MEM NEAA, 1 mM sodium pyruvate, and 0.5 mg/ml hygromycin B (all from Thermo Fisher). SupT1-R5 cells (Didigu et al., 2014) were maintained at 37°C and 5% CO_2_ in RPMI 1640 supplemented with 300 mg/l L-glutamine (Thermo Fisher), 10% FBS (Sigma-Aldrich), and 1% penicillin/streptomycin (Thermo Fisher). The sex of HEK293T, TZM-bl, 293-6E, Expi293, and CHO cell lines is female, and the sex of SupT1-R5 cells is male. Cell lines were not specifically authenticated.

### Method Details

#### Clinical Samples

Peripheral blood mononuclear cells (PBMCs) were isolated by density-gradient centrifugation and stored at −150°C in 90% FBS and 10% DMSO (Sigma-Aldrich) until further use. Plasma and serum samples were stored at −80°C until further use.

#### Serum and Plasma IgG Isolation

Serum and plasma samples were heat-inactivated (56°C for 40 min) and incubated with Protein G Sepharose (GE Life Sciences) overnight at 4°C. IgGs were eluted from Protein G in chromatography columns using 0.1 M glycine (pH = 3.0) and buffered in 1 M Tris (pH = 8.0). Subsequently, buffer exchange to PBS and antibody concentration was performed using Amicon 30 kDa spin membranes (Millipore). Purified IgGs were stored at 4°C until further use.

#### Single Cell Sort

B cells were isolated from PBMCs using the Pan B Cell Isolation Kit, B Cell Isolation Kit II, or IgG^+^ Memory B Cell Isolation Kit (Miltenyi Biotec). Isolated cells were labeled with anti-human CD19-AF700 (BD), anti-human IgG-APC (BD), DAPI (Thermo Fisher), and the respective HIV-1 Env bait for 30 minutes on ice. BG505_SOSIP.664_-GFP or biotinylated YU2_gp140_ that was labeled with Streptavidin-PE (BD) were used as HIV-1 Env baits. Env-reactive CD19^+^IgG^+^DAPI^-^ single cells were sorted into 96-well plates containing 4 μl of lysis buffer (0.5x PBS, 10 mM DTT (Thermo Fisher), 2 U/μl RNasin (Promega), and 1 U/μl RNaseOUT (Thermo Fisher)) per well using a BD FACSAria Fusion. Plates were stored at −80°C until further use.

#### Single Cell cDNA Synthesis and PCR

Sorted cells were incubated with 0.75 μl Random Hexamer Primer (Thermo Fisher), 0.5 μl NP-40 (Thermo Fisher), and 5.6 μl RNase-free H_2_O for 1 min at 65°C. Subsequently, 3 μl of 5x RT Buffer (Thermo Fisher), 0.5 μl dNTPs mix (25 mM, Thermo Fisher), 1 μl DTT (100 mM, Sigma Aldrich), 2.05 μl of RNase-free H_2_O, 0.1 μl RNasin (40 U/μl, Promega), 0.1 μl RNaseOUT (40 U/μl, Promega), and 0.25 μl Superscript IV (200 U/μl, Thermo Fisher) were added and samples were incubated at 42°C for 10 min, 25°C for 10 min, 50°C for 10 min, and 94°C for 5 min. Antibody sequences for single cell analysis were amplified by semi-nested PCRs using Platinum Taq DNA Polymerase or Platinum Taq Green Hot Start DNA Polymerase (Thermo Fisher) and previously described primers, including the novel OPT5/oPR-primer set optimized for detection of highly mutated IgG sequences (Kreer et al., 2019), using the OPT5/oPR-primer mix and CG_RT 5′-AGGTGTGCACGCCGCTGGTC (Ozawa et al., 2006) for the 1^st^ PCR, and the OPT5/oPR-primer mix and IgG_Internal RT 5′-GTTCGGGGAAGTAGTCCTTGAC (Tiller et al., 2008) for the 2^nd^ PCR. First-round PCR was run at 94°C for 2 min; followed by 50 cycles of 94°C for 30 s, 55°C for 30 s, and 72°C for 55 s. Second-round PCR was run at 94°C for 2 min; followed by 50 cycles of 94°C for 30 s, 55°C for 30 s, and 72°C for 45 s. Second-round PCR products were sequenced by Sanger sequencing and used for further sequence analyses.

#### Antibody Sequence Analysis

Sequences with a mean Phred score ≥ 28 and a minimal length of 240 nucleotides were annotated with IgBLAST (Ye et al., 2013) and trimmed from framework region (FWR) 1 of the variable region to the end of the J gene. Base calls with a Phred score < 16 were masked and sequences with > 15 masked nucleotides, frameshifts, or stop codons were excluded from further analyses. To analyze the sequences for potential clonalities, all productive heavy chain sequences were grouped by identical V genes and the pairwise Levenshtein distance of their CDRH3s was determined. Individual sequences were grouped into clones when they shared the same V gene and had a minimal CDRH3 identity of 75%. After 10 rounds with a randomized input of sequences, the result that yielded the lowest number of unassigned (non-clonal) sequences was selected for further analyses. All clones were re-validated manually by the investigators in order to identify shared mutations. Sequences that were initially assigned to different clones but shared the same VDJ genes and amino acid and/or silent nucleotide mutations were subsequently grouped into subclones. Nucleotide sequence identity to germline was calculated using IgBLAST. The maximum-likelihood phylogenetic tree in Figure 1D was generated using nucleotide sequences of heavy-chain V genes (FWRH1-FWRH3) of subclones 4.1, 4.2, 4.3, and 4.4 (n = 86 sequences) and of the IGHV1-46^∗^01 *Homo sapiens* allele (GenBank X92343.1). All sequences were aligned using ClustalW (Geneious R10; cost matrix: IUB; gap open cost: 15; gap extend cost: 6.66) and the maximum-likelihood phylogenetic tree was calculated using PhyML with 1,000 bootstrap replicates (Guindon et al., 2010) (substitution model: general time reversible [GTR]; Geneious R10). The best-scoring tree was then rooted to IGHV1-46^∗^01.

#### Antibody Cloning and Production

For cloning of single cell-derived antibodies, the 1^st^ PCR product of single cell-PCR was used as template and amplified using Q5 Hot Start High Fidelity DNA Polymerase (New England Biolabs) and specific forward- and reverse primers that resembled the respective nucleotide sequence of the V- and J-regions (Tiller et al., 2008) with expression vector overhangs for subsequent sequence and ligation independent cloning (SLIC). PCR was run at 98°C for 30 s; 35 cycles of 98°C for 10 s, 65°C for 30 s, and 72°C for 30 s; and 72°C for 2 min. 561_01_18_ΔINS was generated by cloning a synthesized (Eurofins Genomics) heavy-chain variable region DNA fragment of 1-18 lacking the CDRH1 insertion (^28^PYTDDD^33^). PCR products or synthesized DNA fragments were cloned into human antibody expression vectors (IgG1, kappa, or lambda chain) by SLIC assembly as previously described (von Boehmer et al., 2016). Antibodies were produced in 293-6E cells (National Research Council Canada) by transfection using 25 kDa branched polyethylenimine (PEI) (Sigma-Aldrich). Cells were maintained at 37°C and 6% CO2 in FreeStyle 293 Expression Medium (Thermo Fisher) and 0.2% penicillin/streptomycin (Thermo Fisher). 5-7 days after transfection, culture supernatants were harvested, filtered, and incubated with Protein G Sepharose (GE Life Sciences) overnight at 4°C. Antibodies were eluted from chromatography columns using 0.1 M glycine (pH = 3.0) and buffered in 1 M Tris (pH = 8.0). Subsequent buffer exchange to PBS and antibody concentration was performed using Amicon 30 kDa spin membranes (Millipore). Antibodies were filter-sterilized using Ultrafree-CL or Ultrafree-MC 0.22 μm membranes (Millipore) and stored at 4°C.

#### Pseudovirus Production

Pseudoviruses for the 12-strain global screening panel and f61 finger printing panel were produced in HEK293T cells by co-transfection with pSG3ΔEnv plasmid as described previously (Doria-Rose et al., 2017, Hraber et al., 2017, Sarzotti-Kelsoe et al., 2014, Seaman et al., 2010). Single genome sequencing (SGS)-derived pseudoviruses were generated by co-transfection of SGS-derived CMV promoter-Env products and pSG3ΔEnv as previously described (Kirchherr et al., 2007). For sequences obtained from mice, *env/rev* cassettes were amplified from the first-round SGS PCR product using primers env1Atopo 5′-CACCGGCTTAGGCATCTCCTATGGCAGGAAGAA and envB3in 5′-CACCTTAGGCATCTCCTATGGCAGGAAGAAG. Pseudoviruses were only produced from sequences containing no ambiguities. For patient-derived sequences, *env/rev* cassettes were amplified from the first-round SGS product using primers env1Atopo and Rev19 5′-ACTTTTTGACCACTTGCCACCCAT. CMV promoter and *env/rev* overlap PCR was performed using primers CMVenv 5′-AGTAATCAATTACGGGGTCATTAGTTCAT and Rev19. Mouse-derived sequences were amplified using the Platinum Taq High Fidelity Polymerase (Thermo Fisher), patient-derived sequences using the Phusion Hot Start Flex Polymerase (New England Biolabs).

#### Neutralization Assays

Neutralization assays were performed as previously described (Sarzotti-Kelsoe et al., 2014). In brief, pseudoviruses and dilution series of antibodies or purified IgG were mixed and co-incubated at 37°C for 1 h, followed by the addition of TZM-bl cells at a final concentration of 10^4^ cells per well on a 96-well plate in 250 μl medium supplemented with DEAE-dextran at a final concentration of 10 μg/ml. Following a 2-day incubation at 37°C and 5% CO_2_, 150 μl of culture supernatant was removed and 100 μl luciferase assay reagent was added. After a 2 min incubation, 150 μl of lysate was transferred to a black microtiter assay plate and luminescence was determined using a luminometer. After subtracting background relative luminescence units (RLUs) of non-infected TZM-bl cells, 50% and 80% inhibitory concentrations (IC_50_s and IC_80_s) were determined as the antibody/IgG concentrations resulting in a 50%/80% RLU reduction compared to untreated virus control wells. Murine leukemia virus (MuLV)-pseudotyped virus was used to determine unspecific activity. Initial screening of isolated antibody clonal members was performed using a single dilution series per antibody. Antibodies or purified serum IgG in all further neutralization assays were tested in duplicates. For screening assays, assays against culture-derived viruses, assays of pseudovirus mutant variants, and assays of IDC561-derived pseudoviruses, bioluminescence was determined after adding a luciferin/lysis-buffer (10 mM MgCl2, 0.3 mM ATP, 0.5 mM Coenzyme A, 17 mM IGEPAL (all Sigma-Aldrich), and 1 mM D-Luciferin (GoldBio) in Tris-HCL). For assays against the 119-pseudovirus panel, the 100-pseudovirus clade C panel, and the f61 panel, bioluminescence was determined after adding Bright-Glo reagent (Promega).

#### Neutralization Fingerprinting Panel-Based Antibody Epitope Prediction

Computational epitope prediction of serum IgG neutralizing activity was conducted as previously described (Doria-Rose et al., 2017). In brief, neutralizing serum IgG activity was determined against the 20 pseudoviruses included in the f61 fingerprinting panel by a TZM-bl cell neutralization assay as described above. The determined IgG neutralization fingerprint is compared to the fingerprint of 10 bNAbs picked as reference for their specific epitope, and the prevalence of these reference antibody epitope specificities is computationally predicted and assigned a delineation score between 0 (low) and 1 (high).

#### HIV-1 Envelope Protein Production and Purification

YU2_gp120_, YU2_gp140_ (foldon trimer), and BaL_gp140_ (foldon trimer) (Pietzsch et al., 2010) were produced in 293-6E cells after transfection with polyethylenimine. Proteins were purified from culture supernatants using Ni-NTA Agarose beads (Macherey-Nagel) according to the manufacturer’s instructions and stored at −80°C until further use after buffer exchange to PBS. eOD-GT8 was produced as previously described (Dosenovic et al., 2019). 93THO527 (Anderson et al., 2000) was produced in 293-6E cells in the presence of kifunensine at a concentration of 1 mg/l.

#### HIV-1 Env ELISAs

High-binding ELISA plates (Corning) were coated with HIV-1 Env antigens at 2 μg/ml in PBS overnight at 4°C. Wells were blocked with 3% BSA (Sigma Aldrich) in PBS for 60 min at 37°C. HIV-1 antibodies were diluted in PBS and incubated for 60 min at RT, followed by horseradish peroxidase (HRP)-conjugated anti-human IgG (Jackson ImmunoResearch) diluted 1:1,000 in 3% BSA in PBS for 60 min at room temperature (RT). Absorbance was determined on a microplate reader (Tecan) after addition of ABTS solution (Thermo Fisher). Plates were washed with 0.05% Tween 20 (Carl Roth) in PBS between each step.

#### Competition ELISAs

Antibodies of interest were biotinylated using the EZ Link Sulfo NHS Biotin and Labeling Kit (Thermo Fisher) according to the manufacturer’s instructions, followed by a buffer exchange to PBS using Amicon 10 kDa centrifugation filter membranes (Millipore). High-binding ELISA plates (Corning) were coated with anti-6x His tag antibody (Abcam) at 2 μg/ml overnight at 4°C. Wells were blocked with 3% BSA in PBS for 60 min at 37°C, and incubated with BG505_SOSIP.664_-His at 2 μg/ml in PBS for 60 min at 37°C. Competing antibodies were incubated in a 1:3 dilution series starting at a concentration of 32 μg/ml in PBS for 60 min at RT. Biotinylated antibodies of interest were diluted to 0.5 μg/ml in 3% BSA in PBS and incubated for 60 min at RT, followed by peroxidase-streptavidin (Jackson ImmunoResearch) diluted 1:5,000 in 1% BSA/0.05%Tween 20 in PBS. Absorbance at 415 nm was determined on a microplate reader (Tecan) after addition of ABTS solution (Thermo Fisher). Plates were washed with 0.05% Tween 20 in PBS between each step.

#### Generation of HIV-1_YU2_ and HIV-1_BG505_ Pseudovirus Mutants

Point mutations were introduced into HIV-1_YU2_ and HIV-1_BG505_ envelope expression plasmids using either the QuikChange II XL Site-Directed Mutagenesis Kit (Agilent) or the Q5 Site-Directed Mutagenesis Kit (New England Biolabs). Pseudoviruses were produced as described above.

#### Recombinant HIV-1 Production

Replication-competent recombinant HIV-1 (YU2 *env* in NL4-3 backbone (Zhang et al., 2002)) was produced by transfection of HEK293T cells using FuGENE 6 Transfection Reagent (Promega). Harvested viral supernatants were stored at −80°C to −150°C.

#### HIV-1-Infected Humanized Mice and Viral Load Measurements

Humanized NRG mice were challenged with replication-competent HIV-1 intraperitoneally. HIV-1-infected mice were treated using 0.22 μm-filtered monoclonal antibodies diluted in PBS, starting 25–26 days after viral challenge. Antibodies were injected subcutaneously. Following a 1-mg loading dose per antibody, doses of 0.5 mg per antibody were injected every 3-4 days. Plasma RNA was extracted from EDTA plasma samples using the MinElute Virus Mini Spin Kit (QIAGEN), including an on-column DNase I (QIAGEN) digestion step. Viral loads were determined by quantitative real-time PCR using *pol*-specific primers 5′-TAATGGCAGCAATTTCACCA and 5′-GAATGCCAAATTCCTGCTTGA, and 5′-/56-FAM/CCCACCAACARGCRGCCTTAACTG/ZenDQ/ as probe, as previously described (Horwitz et al., 2013). qPCR was performed on a LightCycler 480 II (Roche) using the Taqman RNA-to-Ct 1-Step-Kit (Thermo Fisher). An HIV-1_YU2_ standard produced by infection of SupT1-R5 cells was included for every PCR run, and HIV-1 copy number of the standard was determined using the quantitative cobas 6800 HIV-1 kit (Roche). The limit of accuracy of the qPCR was determined as 384 copies/ml. Log_10_ changes for viral loads < 384 copies/ml were calculated by assigning a copy number of 383 copies/ml.

#### Humanized Mouse Plasma RNA-Derived Single Genome Sequencing

Single genome sequencing has been described previously (Salazar-Gonzalez et al., 2008). Plasma RNA was extracted using the MinElute Virus Spin Kit (QIAGEN), including a DNase I (QIAGEN) digestion step. cDNA was generated from plasma RNA using the antisense primer YB383 5′-TTTTTTTTTTTTTTTTTTTTTTTTRAAGCAC (Horwitz et al., 2017) and Superscript IV (Thermo Fisher) according to the manufacturer’s protocol, followed by incubation with 0.25 U/μl RNase H (Thermo Fisher) at 37°C for 20 min. *Env* cDNA was subsequently amplified by nested PCR at dilutions that yield < 30% positive PCR reactions so that > 80% of positive reactions would be amplified from a single virion (based on Poisson distribution). First-round PCR was conducted using primers YB383 5′-TTTTTTTTTTTTTTTTTTTTTTTTRAAGCAC and YB50 5′-GGCTTAGGCATCTCCTATGGCAGGAAGAA, and run at 94°C for 2 min; 35 cycles of 94°C for 30 s, 55°C for 30 s, and 72°C for 4 min; and 72°C for 15 min. 1 μL of first-round PCR product was used as template for the second-round PCR that was conducted using primers YB49 5′-TAGAAAGAGCAGAAGACAGTGGCAATGA and YB52 5′-GGTGTGTAGTTCTGCCAATCAGGGAAGWAGCCTTGTG, and run at 94°C for 2 min; 45 cycles of 94°C for 30 s, 55°C for 30 s, and 72°C for 4 min; and 72°C for 15 min. PCR was performed using the Platinum Taq Green Hot Start DNA Polymerase (Thermo Fisher) or Phusion Hot Start Flex DNA Polymerase (New England Biolabs).

#### Illumina Dye Sequencing of Humanized Mouse SGS-Derived *env* Amplicons

Libraries of purified PCR products were prepared for Illumina dye sequencing as described before with modifications (Kryazhimskiy et al., 2014, Schoofs et al., 2016). In brief, PCR products were cleaved by tagmentation using the Nextera DNA Library Prep Kit (Illumina). Indices (Nextera Index Kit, Illumina) were added by limited cycle PCR using the KAPA HiFi HotStart ReadyMix (Roche), followed by adaptor addition (P1, 5′-AATGATACGGCGACCACCGA; P2, 5′-CAAGCAGAAGACGGCATACGA) by limited cycle PCR using the KAPA HiFi HotStart ReadyMix. PCR products were purified using AMPure XP beads (Beckman Coulter), pooled, and sequenced using the MiSeq 300-cycle Nano Kit v2 (Illumina) spiked with approximately 10% PhiX. Paired-end reads were assembled as previously described (Gaebler et al., 2019). For further analyses, a consensus sequence was generated and nucleotides with < 75% identity across reads were defined as ambiguities. Only full-length envelope sequences with high base call quality, less than 10 ambiguities, and no early stop-codons (unless due to ambiguities) were analyzed. Otherwise acceptable sequences showing ambiguities resulting in stop-codons or a frameshift were corrected manually.

#### Human Plasma RNA-Derived Single Genome Sequencing

Plasma RNA was extracted using the MinElute Virus Spin Kit (QIAGEN), including a DNase I (QIAGEN) digestion step. cDNA was generated using the antisense primer envB3out 5′-TTGCTACTTGTGATTGCTCCATGT and SuperScript III Reverse Transcriptase (Thermo Fisher), followed by an RNase H digest (Thermo Fisher). *Env* cDNA was subsequently amplified as described previously with modifications (Mendoza et al., 2018, Salazar-Gonzalez et al., 2008). PCR was performed using the Phusion Hot Start Flex DNA Polymerase (New England Biolabs). First-round PCR was run at 98°C for 45 s; 35 cycles of 98°C for 15 s, 55°C for 30 s, and 72°C for 4 min; and 72°C for 15 min. 1 μL of first-round PCR product was used as template for the second-round PCR which was run at 98°C for 45 s; 45 cycles of 98°C for 15 s, 55°C for 30 s, and 72°C for 4 min; and 72°C for 15 min. Purified PCR products were sequenced using Sanger sequencing and analyzed using Geneious software (Geneious).

#### Bulk Viral Outgrowth Cultures

CD4^+^ T cells were isolated from PBMCs of HIV-1-infected individuals using the CD4^+^ T cell isolation MACS kit (Miltenyi Biotec) and stimulated by co-culture with irradiated (50 Gy) healthy donor PBMCs in T cell medium (RPMI 1640 supplemented with 300 mg/l L-glutamine (Thermo Fisher), 10% FBS (Sigma-Aldrich), and 1% penicillin/streptomycin (Thermo Fisher)) in the presence of 1 μg/ml PHA-M (Sigma-Aldrich) and 100 U/ml interleukin-2 (IL-2) (Miltenyi Biotec). One day later, medium was changed to T cell medium supplemented with 100 U/ml IL-2 and 5 μg/ml polybrene (Sigma-Aldrich). In addition, healthy donor PBMCs were added that had been stimulated for two days in T cell medium supplemented with 1 μg/ml PHA-M and 100 U/ml IL-2. Before addition, donor PBMCs were depleted of CD8^+^ T cells using CD8 MACS microbeads (Miltenyi Biotec). Additional CD8^+^ T cell-depleted donor PBMCs were added weekly. Culture supernatants were monitored for p24 production using the Architect HIV Ag/Ab combo assay (Abbott), and p24-positive culture supernatants were stored at −80°C to −150°C after harvesting.

#### *In vivo* Antibody Pharmacokinetic Analysis

NOD.Cg-*Rag1*^*tm1mom*^ *Il2rg*^*tm1Wjl*^/SzJ mice (The Jackson Laboratory) aged 33-42 weeks were intravenously injected (tail vein) with 0.5 mg of purified antibody in PBS. Total serum concentrations of human IgG were determined by ELISA as previously described with minor modifications (Klein et al., 2012). In brief, high-binding ELISA plates (Corning) were coated with anti-human IgG (Jackson ImmunoResearch) at a concentration of 2.5 μg/ml overnight at RT. Subsequently, wells were blocked with blocking buffer (2% BSA (Carl Roth), 1 μM EDTA (Thermo Fisher), and 0.1% Tween 20 (Carl Roth) in PBS). To generate a standard curve, human IgG1 kappa purified from myeloma plasma (Sigma-Aldrich) was diluted in PBS. Serial dilutions of the IgG standard (in duplicates) and serum samples in PBS were incubated for 90 min at RT, followed by HRP-conjugated anti-human IgG (Jackson ImmunoResearch) diluted 1:1,000 in blocking buffer for 90 min at RT. Following the addition of ABTS (Thermo Fisher), optical density at 415 nm was determined using a microplate reader (Tecan). Plates were washed with 0.05% Tween 20 in PBS between each step. Serum samples obtained before the antibody injection confirmed baseline absence of human serum IgG.

For determination of bNAb-levels following treatment interruption in HIV-1-infected humanized mice, high-binding ELISA plates (Corning) were coated overnight with BG505_SOSIP.664_ at a concentration of 2 μg/ml at 4°C. Subsequently, wells were blocked with 3% BSA in PBS for 5 h at RT. Plasma samples were inactivated in 1% Triton X-100 (Carl Roth) for 1 h at RT. Triton X-100-treated 1-18 diluted in PBS was used as standard in duplicates. Serial dilutions of plasma samples in PBS and standard were incubated for 90 min at RT, followed by HRP-conjugated anti-human IgG (Jackson ImmunoResearch) diluted 1:2,000 in 3% BSA in PBS for 90 min at RT. Following the addition of ABTS (Thermo Fisher), optical density at 415 nm was determined using a microplate reader (Tecan). Plates were washed with 0.05% Tween 20 in PBS between each step.

#### HEp-2 Cell Assay

HEp-2 cell autoreactivity analysis was performed using the NOVA Lite Hep-2 ANA Kit (Inova Diagnostics) according to the manufacturer’s instructions using monoclonal antibodies at a concentration of 100 μg/ml in PBS. Images were acquired using a DMI 6000 B fluorescence microscope (Leica) with 3 s exposure at 100% intensity and gain 10.

#### Unbiased B Cell Repertoire Analyses

B cells were isolated from PBMCs using CD19 microbeads (Miltenyi Biotec) and stained with DAPI (Thermo Fisher), CD20-AF 700, IgG-APC, IgD-Pe-Cy7, IgM-FITC, and CD27-PE (all BD Biosciences) for 30 min on ice. 200,000 CD20^+^IgG^+^IgM^-^IgD^-^CD27^-^ B cells were sorted into FBS (Sigma-Aldrich) using a BD FACSAria Fusion, and RNA of sorted B cells was isolated with the RNeasy Micro Kit (QIAGEN). cDNA was generated by template-switch reverse transcription according to the SMARTer RACE 5′/3′ manual using the SMARTScribe Reverse Transcriptase (Takara) with a template-switch oligo including an 18-nucleotide unique molecular identifier. Heavy-chain variable regions were amplified with an IgG-specific nested PCR and amplicons were used for library preparation and MiSeq 2x300 bp sequencing (Illumina). Raw NGS reads were pre-processed and assembled to final sequences as previously described (Ehrhardt et al., 2019).

#### Mutational Antigenic Profiling

Mutational antigenic profiling has been previously described (Dingens et al., 2017, Dingens et al., 2019). Briefly, 5x10^5^ infectious units of two independently generated HIV-1_BG505_ mutant virus libraries (Haddox et al., 2018) were neutralized with both 4 μg/ml or 8 μg/ml of 1-18 for one hour. Neutralized libraries were then used to infect 1x10^6^ SupT1.CCR5 cells in R10 (RPMI (GE Life Sciences) supplemented with 10% FBS, 2 mM L-glutamine, and 100 U/ml of penicillin and streptomycin) containing 100 μg/ml DEAE-dextran. Three hours post infection, the cells were resuspended in 1 mL R10. At twelve hours post infection, the non-integrated viral cDNA was isolated from cells via a miniprep. Each mutant virus library was also subjected to a mock selection, and four 10-fold serial dilutions of each mutant virus library were infected into 1x10^6^ cells to serve as an infectivity standard curve from which the overall fraction of the library that survived antibody neutralization was quantified using qPCR (Dingens et al., 2019). Viral cDNA from antibody- and mock-selected samples was then sequenced on an Illumina MiSeq using the previously described barcoded subamplicon sequencing approach (Haddox et al., 2016). Details on the analysis of the resulting data are provided in the Quantification and Statistical Analysis and Data and Code Availability subsections below.

#### Protein Expression and Purification for Cryo-EM Structures

1-18 IgG was expressed by transient transfection in Expi293 cells (Thermo Fisher) and purified from transfected cell supernatants using a HiTrap MabSelect Protein A column (GE Life Sciences). Fab fragments were isolated as described (Diskin et al., 2011) after papain cleavage of 1-18 IgG, removal of Fc by protein A chromatography, and then purification by size exclusion chromatography (SEC) on a Superdex-200 Increase 10/300 column (GE Life Sciences) equilibrated with TBS (20 mM Tris pH 8.0, 150 mM NaCl). 1-55 Fab was expressed as a light-chain C-terminal His_6_-tagged Fab by transient transfection in 293-6E cells (National Research Council of Canada) and purified from supernatants using Ni^2+^-NTA affinity chromatography (GE Life Sciences) followed by SEC purification with a Superdex-200 Increase 10/300 column equilibrated with TBS. All Fabs were stored at 4°C.

BG505_SOSIP.664_ trimer was stably expressed in Chinese hamster ovary cells (kind gift of J.P. Moore and A. Cupo) as described (Chung et al., 2014) and purified from cell culture supernatant over a 2G12 immunoaffinity column followed by SEC purification on a Superdex-200 16/60 column (GE Life Sciences) equilibrated with TBS. RC1_SOSIP.664_ was expressed by transient transfection in 293-6E cells and purified as described (Escolano et al., 2019). Individual SEC fractions of each SOSIP trimer were stored at 4°C.

#### Cryo-EM Sample Preparation

1-18 or 1-55 Fab and 10-1074 Fab were incubated with BG505_SOSIP.664_ or RC1 in a 3:3:1 molar ratio per protomer overnight at room temperature and then purified by SEC on a Superdex-200 Increase 10/300 column. Fab–Env complexes were concentrated to 2.2 mg/ml (1-18 complex) or 0.75 mg/mL (1-55 complex) in TBS, and 3 μL was added to a Quantifoil grid (R2/2 Cu 400 mesh for the 1-18 complex and R1.2/1.3 Cu 300 mesh for 1-55 complex; Electron Microscopy Services) that had been freshly glow-discharged using a PELCO easiGLOW (Ted Pella). Samples were vitrified in 100% liquid ethane using a Mark IV Vitrobot (Thermo Fisher) after blotting for 3-3.5 s with Whatman No. 1 filter paper at 22°C and 100% humidity.

#### Cryo-EM Data Collection and Processing

For the 1-18–BG505–10-1074 complex, micrographs were collected on a Titan Krios transmission electron microscope (Thermo Fisher) operating at 300 kV using EPU automated software (Thermo Fisher). Movies were obtained on a Gatan K2 Summit direct electron detector operating in counting mode at a nominal magnification of 130,000x (1.057 Å/pixel calibrated) using a defocus range of −1 to −2.6 μm. Movies were collected with an 8 s exposure time with a rate of 8 e^-^/pix/s, which resulted in a total dose of ~60 e^-^/Å^2^ over 40 fractions. Movies were motion corrected including dose-weighting using Motioncor2 (Zheng et al., 2017) within Relion-3 (Zivanov et al., 2018). The non-dose-weighted images were used for CTF estimation using Gctf (Zhang, 2016), and micrographs with power spectra that showed poor CTF fits or signs of crystalline ice were discarded. Particles were then picked in a reference-free manner using the Laplacian-of-Gaussian auto-picking function in Relion-3. A total of 352,598 particles were extracted, binned 4x4 (4.23 Å/pixel), and subjected to reference-free 2D classification in Relion-3. Particles corresponding to good classes were re-extracted and un-binned (1.057 Å/pixel). An *ab initio* volume was generated in cryoSPARC (Punjani et al., 2017) from micrographs that were collected from the same grid in a Talos Arctica that was used as an initial model for homogeneous 3D-refinement in Relion 3 (assuming C1 symmetry). Particles were then subjected to 3D classification (C1 symmetry), and classes with low-resolution features were removed. Selected classes that appeared 3-fold symmetric were thus subjected to homogeneous 3D refinement assuming C3 symmetry with a soft mask applied that did not include the Fab C_H_C_L_ domains. Per-particle motion correction and CTF refinement were performed in Relion-3, followed by a final homogeneous 3D refinement. A masked post-processed volume of 230,924 particles resulted in a gold-standard FSC (GSFSC) calculation of 2.5 Å (Scheres and Chen, 2012).

For the 1-55–RC1–10-1074 complex, data collection on a Thermo Fisher 200 kV Talos Arctica cryo-electron microscope equipped with a Falcon 3EC camera, and 10-1074 interactions with RC1 were previously described (Escolano et al., 2019). For analysis of the 1-55–RC1 interaction, we reprocessed the data using Relion-3, following a similar procedure as described above for the 1-18–BG505–10-1074 complex. Compared to the original reconstruction, per-particle motion correction and CTF refinement were done in Relion-3, followed by a final homogeneous 3D refinement. A masked post-processed volume of 110,126 particles resulted in a GSFSC calculation of 3.9 Å.

#### Structure Modeling and Refinement

Initial coordinates were generated by docking individual chains from reference structures into cryo-EM density using UCSF Chimera (Goddard et al., 2007). The following PDB coordinates were used: gp120: 5T3Z; gp41: 6MTJ; 10-1074: 5T3Z; 1-18 and 1-55: 4RWY. These initial models were then refined into cryo-EM maps using one round of rigid body refinement followed by real space refinement. Sequence-updated models were built manually in Coot (Emsley et al., 2010) and then refined using iterative rounds of refinement in Coot and Phenix (Adams et al., 2010). Glycans were modeled at PNGSs in Coot using ‘blurred’ maps processed with a variety of B-factors (Terwilliger et al., 2018). Water molecules were added to the 1-18–BG505–10-1074 model based on local density and distance to hydrogen bonding partners. Validation of model coordinates was performed using MolProbity (Chen et al., 2010) and is reported in Table S6.

#### Structural Analyses

Structural figures were made using PyMOL (Version 1.8.2.1 Schrodinger, LLC) or UCSF Chimera (Goddard et al., 2007). Electrostatic calculations were done using the APBS and PDB2PQR servers (Unni et al., 2011). Buried surface areas (BSAs) were calculated using the PDBePISA server (Krissinel and Henrick, 2007). Local resolution maps were calculated using the Local Res program embedded in Relion-3 (Kucukelbir et al., 2014).

### Quantification and Statistical Analysis

The mutational antigenic profiling data were analyzed with dms_tools2 version 2.5.1 (https://jbloomlab.github.io/dms_tools2/; Bloom, 2015). The fraction surviving and excess fraction surviving statistics have been previously described (Dingens et al., 2019, Doud et al., 2018) and are documented at https://jbloomlab.github.io/dms_tools2/fracsurvive.html. Sequencing wild-type DNA plasmid served as the error control during the calculation of the fraction surviving. The HIV Antibody Database (West et al., 2013) was used for the calculation of Env conservation in 1-18 contact residues and for the analysis of neutralization panel data. Clade B reference sequences were obtained through the Los Alamos National Laboratory HIV Database (Filtered Web Alignment, https://www.hiv.lanl.gov/). Median germline nucleotide identity and CDRH3 lengths of HIV-1 Env-reactive and total IgG^+^ B cells of IDC561 were compared using the Mann-Whitney U-test in Python 3 using the “stats” module in the “scipy” package. For the correlation of the neutralizing activity of 1-18 and serum IgG of IDC561, spearman’s rank correlation coefficient was calculated in Prism (GraphPad). The neutralizing activity of 1-18 and 1-18Δins was compared using the Wilcoxon matched-pairs signed rank test in Prism (GraphPad).

### Data and Code Availability

Heavy and light chain sequences of tested monoclonal antibodies have been deposited at GenBank (accession numbers MN867951 - MN868062). SGS-derived HIV-1 *env* obtained from HIV-1_YU2_-infected humanized mice and from individual IDC561 have been deposited at GenBank (accession numbers MN870987 - MN871327 and MN871328 - MN871333, respectively). Density maps and atomic coordinates for the 1-18–BG505–10-1074 and 1-55–RC1–10-1074 complexes were deposited in the Electron Microscopy Data Bank (EMDB) and Protein Data Bank (PDB) with accession numbers EMD-20739 and PDB 6UDJ (1-18 complex) and EMD-20740 and PDB 6UDK (1-55 complex). The entire mutational antigenic profiling analysis is available at https://github.com/jbloomlab/MAP_118, and the accompanying Illumina sequencing data is on the NCBI SRA with accession numbers SRX6752366 - SRX6752371.

## Supplemental Information

Table S1. HIV-1-Reactive Antibody Clones Isolated by Single-Cell Cloning from Individual IDC561, Related to Figure 1

Table S2. Binding of Members of Isolated Antibody Clones Against HIV-1 Env by ELISA, Related to Figure 1

Table S3. Neutralizing Activity against Global Pseudovirus Panel, Related to Figures 1 and 2

Table S4. Sequences of V_H_1-46-Derived bNAbs from Individual IDC561, Related to Figures 1 and 2

Table S5. Neutralizing Activity Against Multiclade, Clade C, and Primary Virus Panels, Related to Figure 2

Table S6. Cryo-EM Data Collection, Refinement, and Validation Statistics, Related to Figure 3

Table S7. Plasma SGS-Derived *env* Sequences Obtained from HIV-1_YU2_-Infected Humanized Mice and Antibody Sensitivity, Related to Figures 6 and 7
