# Supplementary material for: Restriction of HIV-1 Escape by a Highly Broad and Potent Neutralizing Antibody
Source: Cell. 2020 Feb 6;180(3):471–489.e22. doi: 10.1016/j.cell.2020.01.010 (PMC7042716; doi:10.1016/j.cell.2020.01.010)
Supplement: Table S1. HIV-1-Reactive Antibody Clones Isolated by Single-Cell Cloning from Individual IDC561, Related to Figure 1 [file mmc1.pdf]

Cell, Volume *180*

Supplemental Information

Restriction of HIV-1 Escape

by a Highly Broad and Potent Neutralizing Antibody

Philipp Schommers, Henning Gruell, Morgan E. Abernathy, My-Kim Tran, Adam S. Dingens, Harry B. Gristick, Christopher O. Barnes, Till Schoofs, Maike Schlotz, Kanika Vanshylla, Christoph Kreer, Daniela Weiland, Udo Holtick, Christof Scheid, Markus M. Valter, Marit J. van Gils, Rogier W. Sanders, Jörg J. Vehreschild, Oliver A. Cornely, Clara Lehmann, Gerd Fätkenheuer, Michael S. Seaman, Jesse D. Bloom, Pamela J. Bjorkman, and Florian Klein

Table S1. *HIV-1-Reactive Antibody Clones Isolated by Single Cell Cloning from Individual IDC561, Related to Figure 1*

		Heavy Chain						Light Chain						Activity		
		Genes			CDRH3			Genes			CDRL3			Binding (ELISA)		Neutralization
Clone	Antibody	V_H	D_H	J_H	Sequence	Length	Notes	κ/λ	V_L	J_L	Sequence	Length	Notes	BG505*	YU2**	Global panel***
1	561_01_35	1-2*02	6-19*01	4*02	ARDRPSVGSGWYPGSD	16 aa		λ	1-47*01	3*02	ASWDDRLSVGV	11 aa		-	-	-
2	561_09_03	1-69*01	6-19*01	2*01	AREGGSPVAAIYWFFDV	17 aa		λ	1-40*01	1*01	QSYDGNNFV	9 aa		-	-	-
3	561_01_05	4-4*07	2-8*01	2*01	VRDRPGILIGAVPRFDF	17 aa		κ	2-29*02	2*01	MQGIYLPHT	9 aa		-	-	-
4.1	561_01_18	1-46*01	3-10*01	6*03	ARDPFGDRAPHYNYHMDV	18 aa	6 aa ins. in CDRH1	κ	3-20*01	3*01	QRYGGTPIT	9 aa		+	+	+
4.1	561_01_21	1-46*01	5-24*01	6*03	ARDPFGDMYPHYNYHMDV	18 aa	6 aa ins. in CDRH1	κ	3-20*01	3*01	QRYGGTPIT	9 aa		+	+	+
4.1	561_01_33	1-46*01	3-10*01	6*03	ARDPFGDMYPHYNYHMDV	18 aa	6 aa ins. in CDRH1	κ	3-20*01	3*01	QRYGGTPIT	9 aa		+	+	+
4.1	561_01_54	1-46*01	3-10*01	6*03	ARDPFGDMYPHYNSHMDV	18 aa	6 aa ins. in CDRH1	κ	3-20*01	3*01	QRYGGTPIT	9 aa		+	+	+
4.1	561_01_55	1-46*01	3-10*01	6*03	ARDPFGDMYPHYNYHMDV	18 aa	6 aa ins. in CDRH1	κ	3-20*01	3*01	QRYGGTPIT	9 aa		+	+	+
4.1	561_02_10	1-46*01	3-10*01	6*03	ARDPFGNMYPHYNYHMDV	18 aa	6 aa ins. in CDRH1	κ	3-20*01	4*01	QRYGGTPIT	9 aa		+	+	+
4.1	561_02_22	1-46*01	3-10*01	6*03	ARDPFGDMYPHYNYHMDV	18 aa	6 aa ins. in CDRH1	κ	3-20*01	3*01	QRYGGTPIT	9 aa		+	+	+
4.1	561_02_27	1-46*01	3-10*01	6*03	ARDPFGDMYPHYNYHMDV	18 aa	6 aa ins. in CDRH1	κ	3-20*01	3*01	QRYGGTPIT	9 aa		+	+	+
4.1	561_02_47	1-46*01	3-10*01	6*03	ARDPFGDMYPHYNYHMDV	18 aa	6 aa ins. in CDRH1	κ	3-20*01	3*01	QRYGGTPIT	9 aa		+	+	+
4.1	561_03_59	1-46*01	3-10*01	6*03	ARDPFGDRAPHYNYHMDV	18 aa	6 aa ins. in CDRH1	κ	3-20*01	4*01	QRYGATPIT	9 aa		+	+	+
4.1	561_05_18	1-46*01	5-24*01	6*03	ARDPFGDMYPHYNYHMDV	18 aa	6 aa ins. in CDRH1	κ	3-20*01	4*01	QRYGGTPIT	9 aa		+	+	+
4.1	561_08_10	1-46*01	3-10*01	6*03	ARDPFGDRAPHYNYHMDV	18 aa	6 aa ins. in CDRH1	κ	3-20*01	4*01	QRYGGTPIT	9 aa		+	+	+
4.1	561_09_23	1-46*01	3-10*01	6*03	ARDPFGERAPHYNYHMDV	18 aa	6 aa ins. in CDRH1	κ	3-20*01	4*01	QRFGATPIT	9 aa		+	+	+
4.1	561_10_07	1-46*01	3-10*01	6*03	ARDPFGDRAPHYNYHMDV	18 aa	6 aa ins. in CDRH1	κ	3-20*01	4*01	QRYGGTPIT	9 aa		+	+	+
4.2	561_08_52	1-46*01	6-13*01	6*03	ARDPLGEKSPAYSHHMDV	18 aa	2 aa del. in FWR1	κ	3-20*01	4*01	QTYGAITPIT	10 aa	2 aa ins. in FWR3	+	+	+
4.2	561_09_89	1-46*01	1-26*01	6*03	ARDPLGEKSPAYSHHMDV	18 aa	2 aa del. in FWR1	κ	3-20*01	4*01	QTYGAITPIT	10 aa	2 aa ins. in FWR3	+	+	+
4.3	561_09_71	1-46*01	3-16*01	6*04	ARDSFGETFRHSGDQPYQMDV	21 aa	2 aa del. in FWR1	κ	3-20*01	4*01	QSYGSITPLI	10 aa		+	+	+
4.4	561_01_23	1-46*01	3-16*01	6*04	ARDSFGETFRGHDQPYQMDV	20 aa	2 aa del. in FWR1	κ	3-20*01	4*01	QSYGSITPLV	10 aa		+	+	+
4.4	561_01_29	1-46*01	3-16*01	6*04	ARDSFGETFRGHDQPYQMDL	20 aa	2 aa del. in FWR1	κ	3-20*01	4*01	QSYGSITPLV	10 aa		+	+	+
4.4	561_02_12	1-46*01	3-16*01	6*04	ARDPFGETFRGHDQPYRMDV	20 aa	2 aa del. in FWR1	κ	3-20*01	4*01	QSYGSITPLV	10 aa		+	+	+
4.4	561_02_21	1-46*01	3-16*01	6*04	ARDPFGETFRGREQPYQMDV	20 aa	2 aa del. in FWR1	κ	3-20*01	4*01	QSYGPITPLV	10 aa		+	+	+
4.4	561_03_07	1-46*01	3-16*01	6*04	ARDPFGETFRGHDQPYRMDV	20 aa	2 aa del. in FWR1	κ	3-20*01	4*01	QSYGSITPLV	10 aa		+	+	+
4.4	561_03_78	1-46*01	3-16*01	6*04	ARDPFGETFRGHDQPYQMDV	20 aa	2 aa del. in FWR1	κ	3-20*01	4*01	QSYGSITPLV	10 aa		+	+	+
6	561_01_30	3-30*18	4-17*01	6*03	VKDGEGVDYGDYTAYYYYMDV	21 aa		λ	1-47*01	1*01	AAWDDSLSGRYV	12 aa		-	-	-
7	561_01_50	3-13*01	3-3*01	6*03	TRGEAPYDFWGGHLDYHYFYMDI	23 aa		κ	2-30*02	2*01	MQASHWPHA	9 aa		+	+	+
8	561_01_02	5-51*01	5-24*01	3*02	ARPLCQGDVTCINAFDI	17 aa		κ	4-1*01	2*01	EQFYDTPYT	9 aa		+	+	-
8	561_02_17	5-51*01	5-24*01	3*02	ARPFCQGDVTCINAFDI	17 aa		κ	4-1*01	2*01	EQFYDTPYT	9 aa		+	+	+
9	561_02_32	4-34*01	3-10*01	6*03	VRYYPLHMDV	10 aa		κ	2-24*01	2*01	MQASQFPHT	9 aa	2 aa del. in FWR1	-	-	-
10	561_08_24	1-69*01	1-26*01	6*02	AVGSNVNGFFHMDH	14 aa	6 aa del. in FWR3	λ	3-10*01	2*01	YLADRNINMGI	11 aa		+	+	+
11	561_02_73	1-69*01	2-21*02	6*03	VRGGDVVGGSVDLTETDYYYQMDV	24 aa		κ	3-15*01	2*03	QQYNNWPPYS	10 aa		+	+	-
13	561_05_16	1-18*01	2-8*02	4*02	ARDLNYPFCTETTCYETLAY	20 aa		κ	1-39*01	2*01	QQSYSTPYT	9 aa		+	+	+
14	561_05_75	1-2*02	3-3*01	6*03	ARAKTATMFDIIRNPKSGNNYMDA	24 aa		κ	3-20*01	4*01	QQYATSVT	8 aa		-	+	-
17	561_05_54	1-69*01	3-9*01	4*02	EGRYKYGSSSTSGPFDS	17 aa		κ	1-33*01	3*01	QQYDNIPPRAT	11 aa		+	+	-
18	561_05_11	1-69*01	3-9*01	4*02	VGRYKYRSASTSGPFDS	17 aa		κ	1-33*01	3*01	QQCDNIPPRAT	11 aa		+	+	-
20	561_02_33	1-69*01	3-9*01	4*02	VARYKYQHGNSGPFDS	16 aa		κ	1-33*01	3*01	QQYDNVPPGAT	11 aa		+	+	-
21	561_05_02	1-69*01	3-3*01	3*02	ARNTISINGDDAFDI	15 aa		λ	2-8*01	2*01	GSLVGNWDVI	10 aa	6 aa del. in CDRL1	-	-	-
22	561_05_44	1-69*11	5-18*01	6*03	ARSSLVTVMDYEAEELEESHYYYMDV	26 aa		κ	4-1*01	4*01	HQYYNTPLT	9 aa		+	+	+
23	561_06_44	1-69*12	3-10*01	6*03	ARGSVDTVLEFETERLEENYYYYMDV	26 aa		κ	4-1*01	4*01	QQYFSIPLT	9 aa		+	+	+
24	561_05_83	3-23*01	4-17*01	4*02	ARGPLLRFFDL	11 aa		λ	2-23*02	2*01	SSYARGTSFL	10 aa		+	+	-
25	561_06_09	3-23*04	3-10*01	4*02	TKDQGLLRAHDY	12 aa		λ	2-14*01	2*01	SSYTSSRTSS	10 aa		+	+	-
26	561_05_65	3-23*04	3-16*01	4*02	ASILTAQISTFITDVGFLH	19 aa		κ	1-9*01	2*01	HQLNSPRYT	9 aa		+	+	-
27	561_05_09	3-30*18	6-13*01	4*02	AKDKAPRARKQGFDF	15 aa		κ	1D-12*01	2*01	QQANSFPFT	9 aa		-	+	-
28	561_06_69	3-30-3*02	5-18*01	4*02	ARGGFTYGLIFDY	13 aa		κ	2-29*03	2*01	LQGVDLPPYT	10 aa		+	+	-
30	561_05_23	4-34*01	6-6*01	5*02	AGTKRVANRPKGGWFDT	17 aa		κ	1-39*01	2*01	QQSYSTPYT	9 aa		-	-	-
32	561_05_10	4-34*01	3-16*01	5*02	ARLRAKNGYVDWFDP	15 aa		λ	1-47*01	1*01	AAWDDSFDYV	10 aa		+	+	-
34	561_07_60	4-34*01	1-7*01	4*02	AGRLAGTRNFNS	12 aa		κ	1-5*03	3*01	QQYNSIERLFT	11 aa		+	+	-
36	561_07_83	4-34*01	6-6*01	4*02	ARGPKGPRPFGGPADQ	16 aa		κ	3-15*01	5*01	QQYYYWPSIT	10 aa		-	+	-
37	561_07_14	1-69*01	3-16*01	6*03	ARGSVDTVMEFGAGRWEENYFYYADV	26 aa		κ	4-1*01	4*01	QQYFTIPLT	9 aa		+	+	-
40	561_07_07	3-23*01	6-25*01	4*02	ASVLAAQISTFITDVGFLH	19 aa		κ	1-9*01	2*01	HQLNSPRYT	9 aa		+	+	-
41	561_05_78	1-69*12	3-16*01	6*03	ATDGGAGAISITAADNPFDYDYSDYSYYLMDV	32 aa		κ	3-20*01	3*01	QQYGSSPVT	9 aa		+	+	+
43	561_07_10	3-7*03	3-9*01	4*02	VRYFDSRASEARLFDF	16 aa		λ	3-10*01	1*01	YSTDKKGLHYV	11 aa		+	+	-
45	561_05_25	3-11*01	2-21*01	3*02	ARDRRQVLFKTGAGDAFSM	19 aa		λ	3-21*02	3*02	QVWDYSRIHCV	11 aa		+	+	+
54	561_09_01	1-69*06	6-6*01	4*02	ARSAAPRFRLDASGLGNLPH	20 aa	9 aa ins. in CDRH2	κ	3-11*01	4*01	QQSSAWPLT	9 aa		+	+	-

Single Cell Sort Summary by Clone

		Identifying Baits	
Clone	Cells	BG505*	YU2**
1	2	+	-
2	18	+	-
3	106	+	-
4.1	37	+	+
4.2	2	+	-
4.3	7	+	+
4.4	42	+	+
6	2	+	-
7	17	+	+
8	12	+	+
9	7	+	-
10	8	+	-
11	9	+	+
13	5	-	+
14	5	+	+
17	4	-	+
18	8	-	+
20	21	+	+
21	5	-	+
22	13	-	+
23	3	-	+
24	6	-	+
25	6	-	+
26	6	-	+
27	5	-	+
28	6	-	+
30	8	-	+
32	7	-	+
34	2	-	+
36	3	-	+
37	4	-	+
40	6	-	+
41	6	+	+
43	2	+	+
45	3	-	+
54	10	+	+

aa, amino acids; ins., insertion; del., deletion; *, $BG505_{SOSIP.664}$; **, $YU2_{gp140}$ fold-on trimer; *** Neutralization defined as neutralizing activity detected against at least one viral strain.
