# Supplementary material for: Restriction of HIV-1 Escape by a Highly Broad and Potent Neutralizing Antibody
Source: Cell. 2020 Feb 6;180(3):471–489.e22. doi: 10.1016/j.cell.2020.01.010 (PMC7042716; doi:10.1016/j.cell.2020.01.010)
Supplement: Table S2. Binding of Members of Isolated Antibody Clones Against HIV-1 Env by ELISA, Related to Figure 1 [file mmc2.pdf]

Table S2. *Binding of Members of Isolated Antibody Clones Against HIV-1 Env by ELISA, Related to Figure 1*

Clone	Antibody	$YU2_{gp140}$ Maximum $OD_{415\text{-}695nm}$*	$YU2_{gp140}$ EC_{50} (µg/ml)	$BG505_{SOSIP.664}$ Maximum $OD_{415\text{-}695nm}$*	$BG505_{SOSIP.664}$ EC_{50} (µg/ml)
1	561_01_35	0.07	N/A	0.10	N/A
2	561_09_03	0.06	N/A	0.08	N/A
3	561_01_05	0.08	N/A	0.08	N/A
4.1	561_01_18	0.75	0.20	1.46	0.07
4.1	561_01_21	0.51	0.20	1.40	0.09
4.1	561_01_33	0.70	0.15	1.39	0.08
4.1	561_01_54	0.62	0.27	1.40	0.11
4.1	561_01_55	0.75	0.34	1.37	0.08
4.1	561_02_10	0.33	0.63	1.25	0.16
4.1	561_02_22	0.85	0.18	1.40	0.13
4.1	561_02_27	0.72	0.36	1.22	0.07
4.1	561_02_47	0.69	0.52	1.21	0.09
4.1	561_03_59	0.93	0.23	1.58	0.14
4.1	561_05_18	0.66	0.18	1.48	0.12
4.1	561_08_10	0.99	0.18	1.59	0.12
4.1	561_09_23	0.80	0.25	0.56	0.30
4.1	561_10_07	0.79	0.21	0.70	0.21
4.2	561_08_52	1.38	0.10	1.67	0.10
4.2	561_09_89	1.42	0.07	1.20	0.12
4.3	561_09_71	1.55	0.05	1.22	0.13
4.4	561_01_23	1.58	0.06	1.52	0.06
4.4	561_01_29	1.38	0.07	1.54	0.07
4.4	561_02_12	1.47	0.06	1.64	0.06
4.4	561_02_21	1.48	0.07	1.58	0.07
4.4	561_03_07	1.49	0.07	1.65	0.08
4.4	561_03_78	1.43	0.06	1.73	0.08
6	561_01_30	0.07	N/A	0.08	N/A
7	561_01_50	1.63	0.31	1.68	0.08
8	561_01_02	1.58	2.20	1.56	0.13
8	561_02_17	1.51	0.99	1.37	0.04
9	561_02_32	0.04	N/A	0.07	N/A
10	561_08_24	1.52	0.91	1.33	0.77
11	561_02_73	1.44	0.10	1.57	0.21
13	561_05_16	1.75	0.06	1.68	0.08
14	561_05_75	1.54	0.05	0.07	N/A
17	561_05_54	1.50	0.04	1.18	3.93
18	561_05_11	1.47	0.05	0.68	9.43
20	561_02_33	1.42	0.04	1.10	5.13
21	561_05_02	0.04	N/A	0.03	N/A
22	561_05_44	1.22	0.15	1.49	0.89
23	561_06_44	0.99	0.47	1.30	1.76
24	561_05_83	0.95	21.4	0.91	9.50
25	561_06_09	1.19	0.43	1.53	0.94
26	561_05_65	1.52	0.04	1.46	5.36
27	561_05_09	1.01	0.25	0.04	N/A
28	561_06_69	1.23	0.06	0.56	N/A
30	561_05_23	0.06	2.53	0.10	4.44
32	561_05_10	1.60	0.15	1.37	0.04
34	561_07_60	0.92	1.35	0.35	0.13
36	561_07_83	1.37	0.15	0.08	4.41
37	561_07_14	1.12	0.38	1.35	0.83
40	561_07_07	1.31	0.12	1.16	4.03
41	561_05_78	1.56	0.06	1.66	0.09
43	561_07_10	1.54	0.22	1.73	0.13
45	561_05_25	1.59	0.10	1.58	0.10
54	561_09_01	1.04	0.89	0.41	0.73

	Maximum $OD_{415\text{-}695nm}$*	EC_{50} (µg/ml)
(white)	< 0.25	> 5.00
(light grey)	0.25 - 0.75	0.50 - 5.00
(medium grey)	0.75 - 1.00	0.25 - 0.50
(dark)	> 1.00	< 0.25

* Antibodies were tested up to 10 µg/ml. 3BNC117 was included as reference on each ELISA plate and ODs were determined when the $OD_{415\text{-}695nm}$ for 3BNC117 was at a median of 1.53 for $YU2_{gp140}$ (range 1.32-1.73) and 1.49 for $BG505_{SOSIP.664}$ (range 1.22-1.70).
OD, optical density; EC50, half-maximal effective concentration.
