# Supplementary material for: Restriction of HIV-1 Escape by a Highly Broad and Potent Neutralizing Antibody
Source: Cell. 2020 Feb 6;180(3):471–489.e22. doi: 10.1016/j.cell.2020.01.010 (PMC7042716; doi:10.1016/j.cell.2020.01.010)
Supplement: Table S3. Neutralizing Activity against Global Pseudovirus Panel, Related to Figures 1 and 2 [file mmc3.pdf]

Table S3. *Neutralizing Activity Against Global Pseudovirus Panel, Related to Figures 1 and 2*

A

Antibodies identified from IDC561

Clone	Antibody	IC$_{50}$ (µg/ml) 398F1	CNE8	CNE55	246F3	X2278	Tro11	CH119	BJOX2000	25710	CE1176	CE0217	X1632	GeoMean*
1	561_01_35	>25	>25	>25	>25	>25	>25	>25	>25	>25	>25	>25	>25	n/a
2	561_09_03	>25	>25	>25	>25	>25	>25	>25	>25	>25	>25	>25	>25	n/a
3	561_01_05	>25	>25	>25	>25	>25	>25	>25	>25	>25	>25	>25	>25	n/a
4.1	561_01_18	0.07	0.02	0.02	0.03	0.02	0.02	0.05	0.04	0.03	0.23	0.04	0.03	0.035
4.1	561_01_21	0.33	0.05	0.02	0.04	0.03	0.03	0.90	0.05	0.05	0.54	0.11	0.07	0.086
4.1	561_01_33	0.21	0.04	0.03	0.06	0.03	0.03	0.17	0.04	0.06	0.64	0.11	0.04	0.071
4.1	561_01_54	4.48	0.08	0.07	0.09	0.07	0.06	0.59	0.13	0.08	1.86	0.25	0.14	0.198
4.1	561_01_55	0.27	0.04	0.05	0.07	0.03	0.04	0.14	0.06	0.05	0.50	0.07	0.07	0.075
4.1	561_02_10	2.36	0.06	0.08	0.12	0.03	0.04	0.33	0.10	0.11	2.95	0.27	0.17	0.172
4.1	561_02_22	0.09	<0.01	0.03	0.01	0.02	0.02	0.06	0.02	0.04	0.38	0.03	0.03	0.032
4.1	561_02_27	0.22	0.02	0.05	0.03	0.02	0.04	0.20	0.05	0.06	0.92	0.07	0.03	0.063
4.1	561_02_47	0.46	0.02	0.04	0.03	0.02	0.03	0.15	0.04	0.05	0.98	0.08	0.05	0.065
4.1	561_03_59	0.27	0.05	0.04	0.06	0.03	0.05	0.18	0.12	0.07	0.99	0.07	0.08	0.090
4.1	561_05_18	0.29	0.03	0.04	0.04	0.02	0.05	0.22	0.05	0.06	0.87	0.06	0.05	0.073
4.1	561_08_10	0.15	0.10	0.05	0.07	0.03	0.04	0.14	0.08	0.07	0.40	0.11	0.05	0.082
4.1	561_09_23	3.97	0.05	0.03	0.07	0.03	0.03	0.23	0.09	0.06	0.39	0.10	2.51	0.135
4.1	561_10_07	0.20	0.04	0.04	0.08	0.03	0.04	0.17	0.05	0.06	0.30	0.12	0.05	0.071
4.2	561_08_52	0.29	0.04	0.04	0.10	0.03	0.05	0.05	0.04	0.13	0.45	0.05	>25	0.074
4.2	561_09_89	0.35	<0.01	0.02	0.04	0.02	0.01	0.04	<0.01	0.04	0.27	<0.01	>25	0.033
4.3	561_09_71	12.95	2.95	<0.01	2.63	0.02	0.01	<0.01	0.02	0.02	0.07	<0.01	>25	0.084
4.4	561_01_23	0.87	0.04	0.02	0.07	<0.01	0.02	<0.01	0.01	0.03	0.09	0.01	>25	0.034
4.4	561_01_29	10.14	0.51	0.04	0.77	0.03	0.02	0.04	0.03	0.06	0.30	0.01	>25	0.110
4.4	561_02_12	3.22	0.08	0.03	0.22	0.03	0.04	0.05	0.03	0.08	0.36	0.04	>25	0.090
4.4	561_02_21	7.27	0.11	0.03	0.45	0.03	0.04	0.08	0.02	0.18	0.34	0.04	>25	0.115
4.4	561_03_07	9.10	0.14	0.04	0.31	0.04	0.04	0.10	0.03	0.08	0.46	<0.01	>25	0.111
4.4	561_03_78	4.32	0.13	0.04	0.22	0.02	0.03	0.05	0.02	0.04	0.29	0.02	>25	0.075
6	561_01_30	>25	>25	>25	>25	>25	>25	>25	>25	>25	>25	>25	>25	n/a
7	561_01_50	>25	>25	>25	>25	>25	3.24	>25	>25	>25	>25	>25	>25	3.24
8	561_01_02	>25	>25	>25	>25	>25	>25	>25	>25	>25	>25	>25	>25	n/a
8**	561_02_17	>5	>5	>5	>5	>5	>5	>5	>5	>5	>5	2.17	>5	2.17
9	561_02_32	>25	>25	>25	>25	>25	>25	>25	>25	>25	>25	>25	>25	n/a
10	561_08_24	>25	>25	>25	>25	>25	0.35	>25	>25	>25	>25	>25	>25	0.350
11	561_02_73	>10	>10	>10	>10	>10	>10	>10	>10	>10	>10	>10	>10	n/a
13	561_05_16	>25	3.10	>25	>25	>25	>25	>25	>25	23.40	>25	>25	>25	8.52
14	561_05_75	>25	>25	>25	>25	>25	>25	>25	>25	>25	>25	>25	>25	n/a
17	561_05_54	>10	>10	>10	>10	>10	>10	>10	>10	>10	>10	>10	>10	n/a
18	561_05_11	>25	>25	>25	>25	>25	>25	>25	>25	>25	>25	>25	>25	n/a
20	561_02_33	>25	>25	>25	>25	>25	>25	>25	>25	>25	>25	>25	>25	n/a
21	561_05_02	>25	>25	>25	>25	>25	>25	>25	>25	>25	>25	>25	>25	n/a
22	561_05_44	>25	>25	>25	>25	>25	>25	>25	>25	8.29	>25	>25	>25	8.29
23	561_06_44	>25	>25	>25	>25	>25	>25	>25	>25	10.83	>25	>25	>25	10.83
24	561_05_83	>25	>25	>25	>25	>25	>25	>25	>25	>25	>25	>25	>25	n/a
25	561_06_09	>10	>10	>10	>10	>10	>10	>10	>10	>10	>10	>10	>10	n/a
26	561_05_65	>25	>25	>25	>25	>25	>25	>25	>25	>25	>25	>25	>25	n/a
27	561_05_09	>25	>25	>25	>25	>25	>25	>25	>25	>25	>25	>25	>25	n/a
28	561_06_69	>25	>25	>25	>25	>25	>25	>25	>25	>25	>25	>25	>25	n/a
30	561_05_23	>25	>25	>25	>25	>25	>25	>25	>25	>25	>25	>25	>25	n/a
32	561_05_10	>25	>25	>25	>25	>25	>25	>25	>25	>25	>25	>25	>25	n/a
34	561_07_60	>25	>25	>25	>25	>25	>25	>25	>25	>25	>25	>25	>25	n/a
36	561_07_83	>25	>25	>25	>25	>25	>25	>25	>25	>25	>25	>25	>25	n/a
37	561_07_14	>17.5	>17.5	>17.5	>17.5	>17.5	>17.5	>17.5	>17.5	>17.5	>17.5	>17.5	>17.5	n/a
40	561_07_07	>25	>25	>25	>25	>25	>25	>25	>25	>25	>25	>25	>25	n/a
41	561_05_78	>25	>25	>25	>25	>25	>25	>25	>25	13.54	>25	>25	>25	13.54
43	561_07_10	>25	>25	>25	>25	>25	>25	>25	>25	>25	>25	>25	>25	n/a
45	561_05_25	>25	6.78	>25	>25	>25	>25	>25	>25	6.11	>25	>25	>25	6.44
54	561_09_01	>25	>25	>25	>25	>25	>25	>25	>25	>25	>25	>25	>25	n/a

IC$_{50}$ (µg/ml)
<0.1
0.1-0.5
0.5-1
1-2
2-5
5-10
>10

* Geometric mean IC$_{50}$ of neutralized viruses.

** Antibody tested at a maximum concentration of 5 µg/ml.

B

1-18 vs. 1-18Δins

Antibody	IC$_{50}$ (µg/ml) 398F1	CNE8	CNE55	246F3	X2278	Tro11	CH119	BJOX2000	25710	CE1176	CE0217	X1632	GeoMean*
561_01_18	0.07	0.02	0.02	0.03	0.02	0.02	0.05	0.04	0.03	0.23	0.04	0.03	0.035
561_01_18_Δins**	0.97	0.04	0.11	0.13	0.03	0.03	0.03	<0.01	0.10	1.64	0.85	0.30	0.114

* Geometric mean IC$_{50}$ of neutralized viruses.

** Engineered 1-18 variant in which the 6 amino acid CDRH1 insertion was removed

CDRH1 561_01_18: `DDPYTDDDTFTKYW`

CDRH1 561_01_18_Δins: `DD------TFTKYW`

IC$_{50}$ (µg/ml)	<0.1	0.1-0.5	0.5-1	1-2
