# Supplementary material for: Restriction of HIV-1 Escape by a Highly Broad and Potent Neutralizing Antibody
Source: Cell. 2020 Feb 6;180(3):471–489.e22. doi: 10.1016/j.cell.2020.01.010 (PMC7042716; doi:10.1016/j.cell.2020.01.010)
Supplement: Table S4. Sequences of VH1-46-Derived bNAbs from Individual IDC561, Related to Figures 1 and 2 [file mmc4.pdf]

Table S4. *Sequences of V_H1-46-Derived bNAbs from Individual IDC561, Related to Figures 1 and 2*

A

Regions (left to right): FWR1, CDR1, FWR2, CDR2, FWR3, CDR3, FWR4

Clone	Antibody	Sequence
	**IGHV1-46*01**	QVQLVQSGAEVKKPGASVKVSCKASGY------TFTSYYMHWVRQAPGQGLEWMGIINPSGGSTSYAQKFQGRVTMTRDTSTSTVYMELSSLRSEDTAVYYCAR------------------------------
**4.1**	**561_01_18**	.GR.F.......R.....RI..R.DDDPYTDDD...K.WT..I......RP..L.V.S.HFARPI.SY..RD.L.L...S.LTA..L..KG.QPD.SGI.F...DPFGDRAP---HYNYHMDVWGGGTAVIVSS
**4.1**	**561_01_55**	.RR.F...T...R......I..R.DDDPYTDDD...K..T..I......PP..L.V.S.HFARPI.SY..RD.L.L...S.LTA..F..RG.QPD..GI.F...DPFGDMYP---HYNYHMDVWGGGTTVIVSA
**4.1**	561_01_21	.RR.F...T...R......I..R.DDDPYTDDD...K..T..I......RP..L.V.S.HFARPI.SY...D.L.L...S.LTA..F..RG.QPD..GI.F...DPFGDMYP---HYNYHMDVWGGGTTVIVSA
**4.1**	561_01_33	.GR.F...T...R......I..R.DDDPYTDDD...K..T..I......RP..L.V.S.HFARPI.SY..RD.L.L...S.LTA..F..RG.QPD..GI.F...DPFGDMYP---HYNYHMDVWGGGTTVIVSA
**4.1**	561_01_54	.GR.F...T...R......I..R.DDDPYTDDD...K..T..I......RP..L.V.S.HFARPI.SY...D.L.L...S.LTA..F..RD.Q.D..GI.F...DPFGDMYP---HYNSHMDVWGGGTTVIVSA
**4.1**	561_02_10	.GR.F...T...R......I..R.DDDPYTDDD...K.HT..I......RP..L.V.S.HYARPI.SY...D.L.L...S.LTA..F..RG.QPD..GI.F...DPFGNMYP---HYNYHMDVWGGGTTVIVSA
**4.1**	561_02_22	.GR.F.......R......I..R.DDDPYTDDD...K..T..I......RP..L.V.S.HFARPI.SY..RD.L.L...S.LTA..F..RG.QPD..GI.F...DPFGDMYP---HYNYHMDVWGGGTTVIVSA
**4.1**	561_02_27	.GR.F...T...R......I..R.DDDPYTDDD...K..T..I......RP..L.V.S.HYARPI.SY..RD.L.L...S.LTA..F..RG.QPD..GI.F...DPFGDMYP---HYNYHMDVWGGGTTVIVSA
**4.1**	561_02_47	.RR.F...T...R......I..R.DDDPYTDDD...K..T..I......RP..L.V.S.HYARPI.SY...D.L.L...S.LTA..F..RG.QPD..GI.F...DPFGDMYP---HYNYHMDVWGGGTTLIVSA
**4.1**	561_03_59	.RR.F...T...R.....RI..R.DDDPYTDDD...K.WT..I......RP..L.V.S.HFARPI.SY...D.L.L...S.LTG..L..KG.QLD.SGI.F...DPFGDRAP---HYNYHMDVWGGGTAVIVSS
**4.1**	561_05_18	.RR.F...T...R......I..R.DDDPYTDDD...K..T..I......RP..L.V.S.HFARPI.SY...D.L.L...S.LTA..F..RG.QPD..GI.F...DPFGDMYP---HYNYHMDVWGGGTTVIVSA
**4.1**	561_08_10	.GR.F.......R.....RI..R.DDDPYTDDD...K.WT..I......RP..L.V.S.HFARPI.SY..RD.L.L...S.LTA..L..KG.QPD.SGI.F...DPFGDRAP---HYNYHMDVWGGGTAVIVSS
**4.1**	561_09_23	.AH.F.....L.R.....RI..R.DDDPYTDDD...K..T..I......RP..L.V.S.HFARPI.SY...D.L.L...S.LTA..L..R...LD..GI.....DPFGERAP---HYNYHMDVWGAGTTVIVSS
**4.1**	561_10_07	.GRFF.......R.....RI..R.DDDPYTDDD...K.WT..I......RP..L.V.S.HFARPI.SY..RD.L.L...S.LTA..L..QG.QPD.SGI.....DPFGDRAP---HYNYHMDVWGGGTAVIVSS
**4.2**	561_08_52	--....W.GG..R.....RI..QCPED------...K..I........R....L.MVS.H..RPFHTSE.RD.L.....IHET.HH.V..G.GVA.SGT.F...DPLGEKSPA---YSHHMDVWGGGATVIVSS
**4.2**	561_09_89	--....W.GG..R.....RI..QCPED------...K..I........R....L.MVS.H..RPFHTSE.RD.L....EIHET.HH.V..G.GVG.SGT.F...DPLGEKSPA---YSHHMDVWGGGATVIVSS
**4.3**	561_09_71	--..A...GG.........I..VTPES------...K.WL..........F..L.VVS.H..RPMF.N..RD.L.L...IH.T.H....RG.T.D...I.....DSFGETFRHSGDQPYQMDVWGGGTNIVVSS
**4.4**	561_01_23	--......GG.RR......I..ETPE.------...K.WL..L.....R......VVS.H..RPMF.FE.RD.L.L...IH.T.H....RG.T.D.....F...DSFGETFRG-HDQPYQMDVWGGGTTVVVSS
**4.4**	561_01_29	--......GG.RR.........ETPE.------...K.WL..L.....R......VVS.H..RPMF.FE.RD.L.L...IH.T.H....RG.T.D.....F...DSFGETFRG-HDQPYQMDLWGGGTTVVVSS
**4.4**	561_02_12	--......GG.RR......I..ETPED------...K.WL..L............VVS.H..RPMF.FE.RD.L.L...IH.T.HF...RG.T.D.........DPFGETFRG-HDQPYRMDVWGGGTTIVVSS
**4.4**	561_02_21	--......GG..R......I..ETPE.------...K.WL..L............VVS.H..RPMF.FE.RD.L.L...IH.T.H....GG.TLD.........DPFGETFRG-REQPYQMDVWGGGTTIVVTS
**4.4**	561_03_07	--......GG.RR......I..ETPED------...K.WL..L............VVS.H..RPMF.FE.RD.L.L...IH.T.HF...GG.T.D.........DPFGETFRG-HDQPYRMDVWGGGTTIVVSS
**4.4**	561_03_78	--......GG.RR......I..ETPE.------...K.WL..L.....R......VVS.H..RPMF.FE.RD.L.L...IH.T.H....GG.T.D.........DPFGETFRG-HDQPYQMDVWGGGTTIVVSS

B

Regions (left to right): FWR1, CDR1, FWR2, CDR2, FWR3, CDR3, FWR4

Clone	Antibody	Sequence
	**IGKV3-20*01**	EIVLTQSPGTLSLSPGERATLSCRASQSVSSSYLAWYQQKPGQAPRLLIYGASSRATGIPDRF--SGSGSGTDFTLTISRLEPEDFAVYYCQQYGS-SP------------
**4.1**	**561_01_18**	.V......AI..V...D.VI.......GLD..H....RF.R..I.T.V.F.T.N..R.T....--......A........V......T....R..G-T.ITFGGGTTLDKK
**4.1**	**561_01_55**	.A......AI..V...D.VI...K..EGL...D....RF.G..I.T.V.F...N..R.T....--.............N.V......T....R..G-T.ITFGGGTTLDKK
**4.1**	561_01_21	.V......AI..V...D.VI...K..EGL...D....RF.G..I.T.V.F.T.T..R.T....--...............V......T....R..G-T.ITFGGGTTLDKK
**4.1**	561_01_33	.V......AI..V...D.VI...K..EGL...D....RF.G..I.T.V.F.T.N..R.T....--.............N.VQ.....T....R..G-T.ITFGGGTTLDKK
**4.1**	561_01_54	.V......AI..V...D.VI...K..EGL...D....RF.S..I.T.V.F...N..R.T....--.............N.V......T....R..G-T.ITFGGGTTLDKK
**4.1**	561_02_10	.V......AI..V...D.VI...K..EGL...D....RF.R..I.T.V.F...T..R.T....--.............N.V......T....R..G-T.ITFGGGTTLDKK
**4.1**	561_02_22	.V......AI..V...D.VI...K..EGL...D....RF.G..I.T.V.FAT.N..R.T....--..............KVQ.....T....R..G-T.ITFGGGTTLDKK
**4.1**	561_02_27	.V......AI..V...D.VI...K..EGL...D....RF.G..I.T.V.F...N..R.T....--.............N.V......T....R..G-T.ITFGGGTTLDKK
**4.1**	561_02_47	.V......AI..V...D.VI...K..EGL...D....RF.S..I.T.V.F...N..R.T....--A............N.V......T....R..G-T.ITFGGGTTLDKK
**4.1**	561_03_59	.V......AI..V...D.VSF.....EGLDT.Q....RF.R..I.T.V.FAT.N..R.T....--......A......N.V......T....R..A-T.ITFGGGTTLDKK
**4.1**	561_05_18	.V......AI..V...D.VI...K..EGL...D....RF.G..I.T.V.F.T.T..R.T....--...............V......T....R..G-T.ITFGGGTTLDKK
**4.1**	561_08_10	.V......AI..V...D.VI.......GLD..H....RF.R..I.T.V.F.T.N..R.T....--......A........V......T....R..G-T.ITFGGGTTLDKK
**4.1**	561_09_23	.V......AI..A...D.VV......EGLD..Q....RF.D..I...VLF.V.N..R.T....--..G...A........V.R....T....RF.A-T.ITFGGGTRLDMN
**4.1**	561_10_07	.V......AI..V...D.VI......EGLDPTH....RF.R..I.T.V.F.T.N..R.T....--.....EA......T.V......T....R..G-T.ITFGGGTTLDKK
**4.2**	561_08_52	AV......V...V....T.I......HGLDTRHVT.F...R........FATYR..S.VS...RATD...A...N...TAV..A...T.F..T..AIT.ITFGGGTKVDLK
**4.2**	561_09_89	AV......V...V....S.V......HGLDTRHVT.F...R........FAT.R..S.VS...RATDG..A...N...TAV..A...T....T..AIT.ITFGGGTKLDLK
**4.3**	561_09_71	.P........................GF.VDH...F.KR..RP.....FET.R..N.S....--...A..AEY.....NV...........S...IT.LIFGGGTRVDVK
**4.4**	561_01_23	.P..............G.......R.GF.ADHV..F.K...RP.....FE..R..S.T.E..--..G...PEY....T.V...........S...IT.LVFGGGTRVDVK
**4.4**	561_01_29	.P..............GV......R.GF.ADHV..F.K...RP.....FET.R..S.T.E..--..G...PEY....T.V...........S...IT.LVFGGGTRVDVK
**4.4**	561_02_12	.P..........V...G.......R.GF.ADHV..F.K...RP.....FE..R..S.T.E..--......PEY....T.V.A.........S...IT.LVFGGGTRVDVK
**4.4**	561_02_21	.AL.............G.......R.GF.ADHV..F.K...RP.....FE..R..S.T.E..--......PEY....T.V...........S...IT.LVFGGGTRVDVK
**4.4**	561_03_07	.P..........V...G.......R.GF.ADHV..F.K...RP.....FE..R..S.T.E..--......PEY....T.V...........S...IT.LVFGGGTRVDVK
**4.4**	561_03_78	.P......................R.GF.ADHV..F.K...RP.....FE..R..S.T.E..--......PEY....T.V...........S...IT.LVFGGGTRVDVK
