# Supplementary material for: Restriction of HIV-1 Escape by a Highly Broad and Potent Neutralizing Antibody
Source: Cell. 2020 Feb 6;180(3):471–489.e22. doi: 10.1016/j.cell.2020.01.010 (PMC7042716; doi:10.1016/j.cell.2020.01.010)
Supplement: Table S5. Neutralizing Activity Against Multiclade, Clade C, and Primary Virus Panels, Related to Figure 2 [file mmc5.pdf]

Table S5. *Neutralizing Activity Against Multiclade, Clade C, and Primary Virus Panels, Related to Figure 2*

A 119 Pseudovirus Multiclade Panel

Virus	Clade	561_01_18 IC_{50}	561_01_18 IC_{80}	561_01_55 IC_{50}	561_01_55 IC_{80}	561_02_12 IC_{50}	561_02_12 IC_{80}	N6 IC_{50}	N6 IC_{80}	3BNC117 IC_{50}	3BNC117 IC_{80}	VRC01 IC_{50}	VRC01 IC_{80}	10-1074 IC_{50}	10-1074 IC_{80}	PGT121 IC_{50}	PGT121 IC_{80}
MS208.A1	A	0.031	0.116	0.046	0.166	0.048	0.169	0.060	0.213	0.020	0.135	0.114	0.695	>20	>20	>20	>20
Q23.17	A	0.017	0.062	0.019	0.068	0.026	0.125	0.060	0.189	0.016	0.035	0.084	0.241	0.004	0.020	0.003	0.019
Q461.e2	A	0.053	0.200	0.079	0.274	0.069	0.280	0.199	0.781	0.064	0.169	0.443	1.444	>20	>20	>20	>20
Q769.d22	A	0.020	0.088	0.021	0.109	0.009	0.046	0.031	0.138	0.009	0.042	0.026	0.103	>20	>20	>20	>20
Q259.d2.17	A	0.018	0.066	0.030	0.104	0.016	0.055	1.011	>5	0.017	0.061	0.074	0.256	3.054	>20	7.516	>20
Q842.d12	A	0.006	0.014	0.008	0.023	0.006	0.015	0.015	0.043	0.003	0.015	0.019	0.062	12.812	>20	0.012	0.043
3415.v1.c1	A	0.076	0.238	0.014	0.040	0.089	0.274	0.040	0.139	0.110	0.270	0.087	0.225	>20	>20	>20	>20
0260.v5.c36	A	0.010	0.042	0.156	0.417	0.030	0.159	0.191	0.706	0.192	0.503	0.617	1.529	0.157	0.443	0.059	0.144
3365.v2.c2	A	0.025	0.105	0.116	0.381	0.015	0.073	0.060	0.161	0.014	0.068	0.048	0.164	0.021	0.179	0.142	1.952
191955_A11	A	0.017	0.068	0.025	0.065	0.021	0.221	0.030	0.139	>20	>20	0.980	7.140	>20	>20	>20	>20
191084 B7-19	A	0.023	0.085	0.056	0.183	0.018	0.092	0.031	0.109	0.066	0.218	0.168	0.567	0.032	0.120	0.021	0.116
9004SS_A3_4	A	0.031	0.113	0.059	0.197	0.036	0.175	0.116	0.321	0.064	0.168	0.505	1.750	0.009	0.026	0.004	0.015
6535.3	B	0.068	0.353	0.178	1.135	0.055	0.300	0.171	0.495	0.339	2.172	1.560	5.637	0.008	0.033	0.003	0.012
QH0692.42	B	0.089	0.397	0.067	0.289	0.211	0.946	0.449	1.931	0.236	0.721	1.465	3.930	0.148	0.595	0.540	9.175
SC422661.8	B	0.009	0.034	0.015	0.049	0.020	0.074	0.067	0.234	0.046	0.138	0.111	0.353	0.062	0.332	0.072	0.378
PVO.4	B	0.017	0.059	0.034	0.148	0.013	0.081	0.067	0.225	0.068	0.185	0.417	1.379	0.070	0.314	0.114	0.491
TRO.11	B	0.015	0.054	0.027	0.088	0.012	0.047	0.161	0.505	0.047	0.104	0.313	1.233	0.013	0.053	0.007	0.036
AC10.0.29	B	0.369	1.772	0.420	3.077	0.296	2.897	0.267	0.902	6.457	>20	1.462	4.317	0.022	0.071	0.026	0.102
RHPA4259.7	B	0.015	0.066	0.020	0.095	0.006	0.020	0.020	0.063	0.023	0.064	0.049	0.158	0.018	0.091	0.013	0.047
THRO4156.18	B	1.584	9.632	0.928	13.784	0.481	2.359	0.565	4.367	2.634	12.176	3.714	17.013	>20	>20	>20	>20
REJO4541.67	B	0.016	0.059	0.020	0.122	0.011	0.042	0.030	0.102	0.028	0.100	0.059	0.230	>20	>20	3.843	>20
TRJO4551.58	B	0.060	0.237	0.084	0.316	0.018	0.064	0.111	0.357	0.068	0.282	0.084	0.258	0.152	0.536	2.732	14.312
WITO4160.33	B	0.006	0.033	0.020	0.074	0.005	0.047	0.079	0.247	0.023	0.088	0.127	0.382	0.179	1.294	0.519	3.532
CAAN5342.A2	B	0.804	3.427	1.314	6.602	0.496	2.446	0.208	0.609	0.633	1.681	1.077	3.298	0.005	0.027	0.007	0.029
WEAU_d15_410_5017	B	0.010	0.059	0.054	0.175	0.004	0.018	0.018	0.063	0.052	0.190	0.114	0.268	0.044	0.179	0.031	0.104
1006_11_C3_1601	B	0.039	0.146	0.059	0.276	0.005	0.043	0.076	0.255	0.035	0.115	0.181	0.520	0.002	0.008	0.003	0.011
1054_07_TC4_1499	B	0.475	1.656	0.408	5.938	0.456	2.661	0.312	1.103	0.088	0.500	0.889	3.772	0.082	0.335	0.066	0.396
1056_10_TA11_1826	B	0.033	0.192	0.051	0.638	0.017	0.169	0.137	0.700	0.267	1.194	1.006	3.514	0.048	0.213	0.014	0.188
1012_11_TC21_3257	B	0.008	0.042	0.023	0.112	0.013	0.084	0.038	0.183	0.022	0.073	0.111	0.406	0.011	0.053	0.005	0.035
6240_08_TA5_4622	B	0.312	1.073	0.315	1.409	0.165	0.518	0.195	0.723	0.254	0.688	0.769	2.204	0.047	0.172	0.055	0.315
6244_13_B5_4576	B	0.023	0.148	0.049	0.173	0.028	0.139	0.074	0.353	0.036	0.109	0.269	0.691	0.129	0.503	0.105	1.596
62357_14_D3_4589	B	0.060	0.258	0.041	0.193	0.019	0.092	0.057	0.267	0.053	0.173	1.066	4.845	>20	>20	2.571	>20
SC05_8C11_2344	B	0.045	0.159	0.066	0.197	0.086	0.308	0.215	0.739	0.134	0.430	0.593	1.793	0.025	0.068	0.030	0.147
Du156.12	C	0.022	0.081	0.081	0.360	0.106	1.995	0.014	0.051	0.031	0.114	0.088	0.248	0.012	0.056	0.005	0.018
Du172.17	C	>20	>20	>20	>20	2.555	>20	0.077	0.446	0.354	3.288	>20	>20	0.040	0.216	0.048	0.768
Du422.1	C	0.063	0.216	0.144	0.477	0.050	0.238	0.027	0.119	>20	>20	>20	>20	0.045	0.153	0.040	0.173
ZM197M.PB7	C	0.091	0.321	0.214	0.699	0.137	0.714	0.124	0.536	0.350	1.145	0.520	1.673	>20	>20	>20	>20
ZM214M.PL15	C	0.048	0.654	0.098	1.333	0.007	0.076	0.125	0.600	0.089	0.692	0.767	3.344	0.434	2.752	0.443	2.353
ZM233M.PB6	C	0.042	0.159	11.256	>20	0.438	6.876	0.084	0.594	0.201	0.901	2.732	10.823	0.025	0.348	2.121	2.673
ZM249M.PL1	C	0.020	0.073	0.060	0.292	5.632	>20	0.031	0.102	0.040	0.300	0.070	0.460	>20	>20	>20	>20
ZM53M.PB12	C	0.256	1.204	0.387	2.175	0.123	0.571	0.319	0.838	0.222	0.846	0.880	3.230	>20	>20	0.001	0.007
ZM109F.PB4	C	9.916	>20	>20	>20	>20	>20	0.108	0.351	0.075	0.763	0.124	0.481	>20	>20	9.124	>20
ZM135M.PL10a	C	1.729	13.124	>20	>20	>20	>20	0.255	1.117	0.060	0.205	0.811	5.016	0.070	0.331	0.870	6.359
CAP45.2.00.G3	C	>20	>20	>20	>20	>20	>20	0.043	0.165	1.350	13.029	5.630	>20	>20	>20	1.317	14.585
CAP210.2.00.E8	C	0.818	4.420	1.044	6.600	>20	>20	>20	>20	9.797	>20	>20	>20	>20	>20	15.205	>20
HIV-001428-2.42	C	0.007	0.019	0.011	0.037	0.008	0.028	0.007	0.024	0.015	0.030	0.019	0.050	0.028	0.149	0.018	0.097
HIV-0013095-2.11	C	0.151	0.534	>20	>20	>20	>20	0.078	0.293	0.258	2.361	0.128	0.318	5.840	>20	>20	>20
HIV-16055-2.3	C	0.019	0.051	0.018	0.079	0.018	0.061	0.021	0.074	4.176	>20	0.099	0.303	>20	>20	0.456	9.310
HIV-16845-2.22	C	0.862	2.869	1.876	8.872	1.039	4.052	0.697	3.592	>20	>20	3.606	13.571	0.793	4.129	5.855	2.644
Ce1086_B2	C	0.092	0.238	0.062	0.322	0.051	0.215	0.058	0.219	0.071	0.237	0.571	1.772	>20	>20	0.001	0.011
Ce0393_C3	C	0.036	0.162	0.056	0.292	>20	>20	0.100	0.506	0.202	0.654	1.039	3.295	>20	>20	>20	>20
Ce1176_A3	C	0.199	0.694	0.544	2.667	0.156	0.805	0.427	1.200	0.194	0.547	2.315	6.331	0.021	0.073	0.014	0.056
Ce2010_F5	C	0.139	0.477	0.197	0.656	0.057	0.206	0.166	0.665	0.054	0.219	0.638	1.924	>20	>20	>20	>20
Ce0682_E4	C	0.015	0.071	0.016	0.070	0.019	0.129	0.042	0.160	0.032	0.133	0.175	0.723	>20	>20	>20	>20
Ce1172_H1	C	0.295	1.051	0.173	0.792	>20	>20	0.515	3.019	>20	>20	>20	>20	0.022	0.098	0.013	0.047
Ce2060_G9	C	0.086	0.408	0.440	2.420	0.091	0.443	0.163	0.672	0.214	0.712	0.444	1.889	>20	>20	>20	>20
Ce703010054_2A2	C	0.032	0.162	0.061	0.431	0.037	0.156	0.163	0.826	0.313	1.338	0.750	2.427	>20	>20	>20	>20
BF1266.431a	C	0.030	0.115	0.104	0.386	>20	>20	0.022	0.079	0.027	0.081	0.073	0.284	>20	>20	>20	>20
246F C1G	C	0.006	0.021	0.051	0.396	>20	>20	0.101	0.455	15.899	>20	7.000	>20	0.033	0.112	0.055	0.284
249M B10	C	0.027	0.099	0.064	0.291	19.962	>20	0.033	0.129	0.084	0.186	0.130	0.272	>20	>20	3.200	>20
ZM247v1(Rev-)	C	0.023	0.106	0.035	0.238	>20	>20	0.053	0.176	>20	>20	0.326	1.223	0.046	0.162	0.029	0.107
7030102001E5(Rev-)	C	0.121	0.417	0.203	0.957	0.091	0.445	0.244	0.794	0.315	1.228	1.065	3.166	0.008	0.022	0.008	0.029
1394C9G1(Rev-)	C	0.287	1.296	6.291	>20	0.941	>20	0.079	0.329	>20	>20	0.574	2.108	0.033	0.112	0.400	3.817
Ce704809221_1B3	C	0.078	0.362	0.114	0.847	0.148	1.121	0.445	1.664	0.074	0.302	0.878	2.728	0.096	0.476	0.039	0.283
3016.v5.c45	D	1.227	7.388	11.424	>20	0.024	0.086	0.020	0.072	0.804	3.007	0.153	0.389	>20	>20	>20	>20
A07412M1.vrc12	D	0.043	0.190	0.128	0.725	0.042	0.329	0.050	0.231	0.029	0.133	0.152	0.561	0.004	0.042	0.008	0.168
231965.c01	D	0.115	0.515	>20	>20	1.641	>20	0.048	0.240	0.042	0.203	0.357	1.188	10.000	>20	>20	>20
231966.c02	D	0.096	0.495	0.312	1.059	0.024	0.161	0.059	0.193	0.218	1.692	0.119	0.523	>20	>20	>20	>20
6405.v4.c34	D	>20	>20	>20	>20	14.354	>20	0.072	0.454	0.171	0.480	2.399	6.239	0.007	0.085	0.018	0.083
X1193_c1	G	0.050	0.151	0.025	0.174	0.360	16.152	0.061	0.235	0.065	0.216	0.138	0.381	0.072	0.285	0.022	0.101
P0402_c2_11	G	0.043	0.100	0.010	0.073	0.035	0.129	0.032	0.088	0.061	0.196	0.222	0.531	0.008	0.030	0.006	0.028
X1254_c3	G	0.027	0.126	0.142	0.684	0.203	0.932	0.031	0.091	0.078	0.223	0.057	0.158	0.075	0.243	0.019	0.085
X2088_c9	G	0.089	0.319	0.165	0.566	0.137	0.534	0.081	0.272	>20	>20	>20	>20	0.002	0.014	0.003	0.014
X2131_C1_B5	G	0.053	0.236	0.365	1.739	0.192	1.990	0.183	0.844	0.357	1.101	0.464	1.446	0.017	0.073	0.007	0.043
P1981_C5_3	G	11.131	>20	>20	>20	10.492	>20	0.131	0.357	0.483	1.791	0.403	0.967	0.002	0.016	0.001	0.012
X1632_S2_B10	G	0.017	0.084	0.020	0.145	>20	>20	0.068	0.244	4.981	19.351	0.109	0.623	>20	>20	>20	>20
3301.v1.c24	AC	0.005	0.023	0.022	0.077	0.010	0.086	0.012	0.043	0.032	0.082	0.126	0.297	0.010	0.044	0.009	0.035
6041.v3.c23	AC	0.004	0.018	0.013	0.037	0.003	0.014	0.014	0.046	0.009	0.049	0.023	0.085	>20	>20	>20	>20
6540.v4.c1	AC	0.005	0.040	0.015	0.079	0.014	1.019	0.064	1.326	>20	>20	>20	>20	>20	>20	>20	>20
6545.v4.c1	AC	0.004	0.020	0.013	0.046	4.100	>20	16.228	>20	>20	>20	>20	>20	>20	>20	>20	>20

Table S5. *Neutralizing Activity Against Multiclade, Clade C, and Primary Virus Panels, Related to Figure 2 (continued)*

119 Pseudovirus Multiclade Panel *(continued)*

Virus	Clade	561_01_18		561_01_55		561_02_12		N6		3BNC117		VRC01		10-1074		PGT121	
		IC_{50}	IC_{80}	IC_{50}	IC_{80}	IC_{50}	IC_{80}	IC_{50}	IC_{80}	IC_{50}	IC_{80}	IC_{50}	IC_{80}	IC_{50}	IC_{80}	IC_{50}	IC_{80}
620345.c01	AE	0.145	1.964	0.222	2.687	>20	>20	4.393	>20	>20	>20	>20	>20	>20	>20	>20	>20
CNE8	AE	0.005	0.030	0.026	0.097	0.022	0.206	0.112	0.483	0.086	0.361	0.678	1.672	>20	>20	>20	>20
C1080.c03	AE	0.020	0.100	0.069	0.492	0.010	0.112	0.383	1.560	0.120	0.790	1.613	9.664	>20	>20	>20	>20
R2184.c04	AE	0.004	0.020	0.011	0.038	0.005	0.027	0.021	0.104	0.032	0.110	0.081	0.313	>20	>20	>20	>20
R1166.c01	AE	0.006	0.026	0.015	0.065	0.006	0.037	0.200	0.959	0.203	0.622	1.642	5.640	>20	>20	>20	>20
R3265.c06	AE	0.010	0.086	0.030	0.266	0.005	0.034	0.040	0.323	0.060	1.952	0.347	2.266	>20	>20	>20	>20
C2101.c01	AE	0.038	0.330	0.077	0.450	0.003	0.036	0.073	0.486	0.060	0.188	0.189	0.709	>20	>20	>20	>20
C3347.c11	AE	0.027	0.090	0.018	0.080	0.016	0.060	0.012	0.054	0.031	0.105	0.077	0.325	>20	>20	>20	>20
C4118.c09	AE	0.040	0.249	0.161	1.108	0.020	0.086	0.089	0.392	0.030	0.187	0.111	0.606	>20	>20	>20	>20
CNE5	AE	0.184	0.868	2.789	15.607	0.137	0.838	0.139	0.618	0.344	1.068	0.332	0.996	>20	>20	>20	>20
BJOX009000.02.4	AE	0.386	1.300	19.308	>20	0.124	0.807	0.353	1.347	0.365	1.277	2.412	5.667	>20	>20	4.156	>20
BJOX015000.11.5	AE	0.117	0.773	0.097	1.148	0.033	0.308	0.067	0.524	0.053	0.303	0.293	1.058	>20	>20	>20	>20
BJOX010000.06.2	AE	0.526	3.408	3.403	14.691	0.228	1.538	0.587	2.595	1.772	6.245	9.188	17.773	>20	>20	>20	>20
BJOX025000.01.1	AE	0.120	0.839	0.054	0.584	0.024	0.238	0.020	0.102	0.063	0.262	4.126	>20	>20	>20	>20	>20
BJOX028000.10.3	AE	0.806	>20	>20	>20	0.027	1.812	0.003	0.013	0.012	0.048	0.532	1.149	>20	>20	>20	>20
T257-31	AG	0.050	0.199	0.105	0.475	0.183	1.345	0.171	0.855	0.133	0.507	2.145	6.753	>20	>20	>20	>20
928-28	AG	0.189	0.672	0.633	2.918	0.133	0.643	0.097	0.461	0.156	0.533	0.436	1.265	0.759	2.915	>20	>20
263-8	AG	0.016	0.058	0.038	0.104	0.019	0.096	0.047	0.213	0.030	0.090	0.200	0.588	0.262	4.362	1.008	7.901
T250-4	AG	0.019	0.067	0.037	0.144	>20	>20	0.016	0.055	>20	>20	>20	>20	0.001	0.005	0.001	0.011
T251-18	AG	0.044	0.137	0.070	0.235	0.044	0.218	0.365	1.905	0.210	0.744	3.419	10.447	0.387	2.427	10.177	3.240
T278-50	AG	>20	>20	10.349	>20	>20	>20	>20	>20	>20	>20	>20	>20	0.942	5.436	>20	>20
T255-34	AG	0.042	0.292	0.793	15.424	>20	>20	0.147	0.682	0.042	0.205	0.435	1.664	0.380	>20	9.198	>20
211-9	AG	0.076	0.283	0.422	2.072	>20	>20	0.358	2.182	0.346	1.197	16.437	>20	0.108	0.445	0.997	7.298
235-47	AG	0.007	0.027	0.015	0.072	0.027	0.166	0.020	0.069	0.025	0.158	0.044	0.169	0.019	0.137	0.184	1.116
CNE19	BC	0.006	0.022	0.011	0.040	0.006	0.028	0.055	0.188	0.023	0.099	0.245	0.861	0.199	>20	0.008	0.080
CNE20	BC	0.033	0.149	0.060	0.275	0.007	0.047	0.025	0.108	>20	>20	8.270	>20	0.001	0.006	0.001	0.007
CNE21	BC	0.039	0.185	0.056	0.230	0.017	0.064	0.067	0.346	19.054	>20	0.441	1.382	0.043	0.141	0.008	0.025
CNE17	BC	0.157	0.566	15.490	>20	0.086	0.481	0.328	1.219	4.893	>20	1.541	4.030	1.791	6.820	9.777	>20
CNE30	BC	0.076	0.274	0.150	0.635	0.075	0.269	0.291	1.039	0.336	1.112	0.825	2.582	0.299	0.948	0.077	0.291
CNE52	BC	0.007	0.026	0.011	0.038	0.003	0.014	0.052	0.144	0.027	0.092	0.215	0.595	1.887	10.909	2.843	>20
CNE53	BC	0.089	0.317	0.215	0.956	0.032	0.116	0.109	0.366	0.066	0.567	0.104	0.301	0.008	0.059	0.013	0.068
CNE58	BC	0.017	0.050	0.027	0.064	0.009	0.028	0.040	0.131	0.360	2.338	0.164	0.456	0.139	0.548	>20	>20
3817.v2.c59	CD	0.131	0.540	0.725	3.717	2.284	>20	0.562	3.584	0.197	0.636	>20	>20	0.734	5.714	18.892	>20
6480.v4.c25	CD	0.023	0.083	0.013	0.062	0.025	0.074	0.038	0.136	0.010	0.040	0.040	0.090	0.006	0.028	0.003	0.041
6952.v1.c20	CD	0.035	0.182	0.309	1.834	0.020	0.100	0.022	0.072	0.120	0.440	0.050	0.149	0.016	0.069	0.060	0.418
6811.v7.c18	CD	0.042	0.227	0.066	0.288	0.074	0.676	0.069	0.235	0.038	0.185	0.168	0.493	0.002	0.009	0.002	0.009
89-F1_2_25	CD	0.007	0.023	0.014	0.039	0.006	0.025	>20	>20	>20	>20	>20	>20	>20	>20	>20	>20
0815.v3.c3	ACD	0.009	0.044	0.011	0.047	0.003	0.014	0.017	0.080	0.015	0.037	0.040	0.103	0.022	0.146	0.020	0.080
3103.v3.c10	ACD	0.050	0.144	0.146	0.453	18.167	>20	0.331	1.223	0.270	1.023	1.193	3.179	0.020	0.057	0.017	0.084
GeoMean of Neutralized		0.048	0.183	0.096	0.346	0.053	0.188	0.086	0.314	0.111	0.339	0.349	0.994	0.049	0.166	0.066	0.169
Breadth (%)		96.6	94.1	92.4	87.4	86.6	78.2	97.5	95.0	88.2	83.2	89.9	85.7	61.3	56.3	65.5	57.1

N6 neutralization data were determined in the same laboratory as data for 1-18 family members, except for 6405.v4.c34 and 0330_V4_C3 which were derived from CATNAP.
VRC01 neutralization data were derived from CATNAP except for four strains that were tested in the same laboratory as 1-18 family members (249M B10, 89-F1_2_25, BJOX015000.11.1, and 231966.c02).
3BNC117, 10-1074, and PGT121 neutralization data were derived from CATNAP.
1-55 tested on the 119-panel was based on earlier sequence variants and contained primer-induced mutations at the start and end of the V genes (VH: 2 aa mut., Vk: 4 aa mut.).

Antibody concentration (μg/ml)	<0.1	0.1 - 0.5	0.5 - 1	1 - 2	2 - 5	5 - 10	>10
Breadth (%)	>95	90 - 95	80 - 90	70 - 80	60 - 70	50 - 60	<50

Table S5. *Neutralizing Activity Against Multiclade, Clade C, and Primary Virus Panels, Related to Figure 2 (continued)*

B 100 Pseudovirus Clade C Panel

Virus	Clade	561_01_18		561_02_12		N6		3BNC117		VRC01		10-1074		PGT121	
		IC_{50}	IC_{80}	IC_{50}	IC_{80}	IC_{50}	IC_{80}	IC_{50}	IC_{80}	IC_{50}	IC_{80}	IC_{50}	IC_{80}	IC_{50}	IC_{80}
0041_V3_C18	C	0.061	0.213	0.066	0.233	0.103	0.285	0.100	0.213	0.360	1.340	>20	>20	2.179	>20
0933_V4_C4	C	>20	>20	>20	>20	0.023	0.065	>20	>20	0.910	>10	>20	>20	>20	>20
0984_V2_C2	C	0.094	0.269	0.128	0.922	0.272	1.319	>20	>20	1.490	>10	0.954	5.375	0.268	2.065
1170887_08	C	0.003	0.014	>20	>20	0.001	0.005	0.004	0.014	0.110	0.420	>20	>20	>20	>20
1245045	C	0.253	1.150	0.294	1.445	0.904	4.006	3.298	>20	3.440	>10	>20	>20	>20	>20
18814602_H8_F3	C	0.015	0.059	0.007	0.047	0.004	0.011	0.007	0.031	0.020	0.110	0.006	0.015	0.001	0.003
19157834_V1	C	0.012	0.040	0.024	0.089	0.015	0.070	0.041	0.170	0.350	1.630	0.019	0.061	0.008	0.029
19252094_A5_G2	C	0.067	0.246	0.062	0.234	0.059	0.210	5.147	>20	0.470	1.720	0.033	0.120	0.025	0.125
19314479_A2_5	C	0.113	0.537	0.076	0.522	0.053	0.248	0.046	0.166	0.840	3.370	>20	>20	>20	>20
19707346_E8_C6	C	0.177	0.597	0.105	0.614	0.040	0.121	19.801	>20	1.730	6.280	>20	>20	>20	>20
20104663_E11_D2	C	>20	>20	>20	>20	0.015	0.059	>20	>20	>10	>10	0.005	0.013	0.008	0.019
20198102_E9_G1	C	0.028	0.072	0.051	0.214	0.053	0.176	0.366	>20	0.160	0.540	0.196	0.845	0.488	1.801
20258279_V2	C	0.078	0.223	0.190	0.633	0.069	0.239	0.303	1.037	0.540	1.950	0.053	0.185	3.417	10.001
20286961_C1_H8	C	0.058	0.187	0.078	0.325	0.144	0.355	0.130	0.458	0.400	1.770	>20	>20	1.375	14.150
20358510_01	C	0.014	0.046	0.050	0.148	0.074	0.204	0.192	1.459	0.390	1.410	8.535	>20	4.502	>20
20417927_07	C	0.396	1.759	>20	>20	>20	>20	>20	>20	>10	>10	>20	>20	>20	>20
20883229_C9_H6	C	0.056	0.149	0.044	0.117	0.015	0.039	18.336	>20	0.400	1.750	0.006	0.025	0.051	0.214
20915593	C	0.046	0.193	>20	>20	0.006	0.029	>20	>20	>10	>10	0.580	2.807	1.359	5.595
20927783	C	>20	>20	>20	>20	0.702	3.581	>20	>20	>10	>10	4.152	>20	0.129	0.553
20965238	C	0.135	0.506	0.087	0.449	0.069	0.259	0.157	1.033	1.100	3.810	>20	>20	>20	>20
21197826_V1	C	0.825	3.273	>20	>20	0.313	1.240	>20	>20	>10	>10	0.551	1.889	1.143	4.309
21203310_G7_C3	C	0.374	2.497	3.976	>20	2.063	8.817	5.812	18.724	>10	>10	0.043	0.271	0.077	0.283
21283649	C	0.074	0.274	0.051	0.271	0.042	0.135	0.165	0.564	0.310	1.480	>20	>20	>20	>20
21492713_B11_E3	C	0.031	0.125	0.055	0.227	0.066	0.227	0.089	0.354	0.140	0.550	0.015	0.049	0.002	0.014
21502011_F12_E2	C	0.309	1.443	0.542	2.632	0.372	1.817	1.582	5.924	9.770	>10	0.016	0.075	0.016	0.047
21561324_D3_B5	C	0.006	0.019	0.015	0.102	0.005	0.017	6.983	>20	0.440	2.050	0.004	0.009	0.081	0.618
234_F1_16_57	C	0.028	0.095	4.740	>20	0.039	0.104	>20	>20	>10	>10	>20	>20	0.581	2.810
235080_3G7_REV	C	0.322	17.831	0.027	0.471	0.028	0.062	>20	>20	8.410	>10	0.001	0.004	0.001	0.003
2768732_C5_16	C	0.246	0.930	0.287	1.524	0.271	1.302	0.304	1.142	1.600	4.420	>20	>20	>20	>20
2865_A11_12	C	0.411	1.482	0.315	1.799	0.135	0.500	0.268	1.198	0.880	3.320	2.057	6.610	1.132	5.495
2969249	C	>20	>20	0.125	0.951	0.280	1.857	0.640	3.509	1.370	>10	>20	>20	>20	>20
3312_D6_2	C	0.013	0.050	0.018	0.070	0.065	0.174	0.828	9.739	0.180	0.810	0.043	0.149	>20	>20
3426_V5_C17	C	0.037	0.184	0.027	0.204	0.034	0.126	0.049	0.200	0.260	1.400	0.008	0.024	0.007	0.033
3514597	C	0.103	0.346	0.239	8.256	0.022	0.078	0.038	0.510	0.500	2.280	0.099	0.293	0.021	0.091
3603_C11_13	C	0.040	0.138	0.050	0.178	0.045	0.128	0.015	0.056	0.310	0.980	0.027	0.101	0.032	0.123
541_F1_A7_2	C	0.099	0.434	0.049	0.217	0.032	0.090	0.070	0.246	0.170	0.600	>20	>20	>20	>20
556_F2_3_25	C	0.253	0.837	0.432	1.942	0.151	0.527	4.418	>20	3.320	>10	0.078	0.261	0.045	0.145
569_F1_37_10	C	0.260	1.241	0.062	0.281	0.041	0.114	0.226	1.069	0.300	1.520	0.869	5.453	>20	>20
6022_V7_C24	C	4.221	>20	>20	>20	0.842	2.746	0.021	0.098	1.980	6.980	0.017	0.046	0.023	0.121
6040_V4_C15	C	0.022	0.084	0.013	0.061	0.049	0.179	0.032	0.187	0.150	0.530	>20	>20	>20	>20
6146_V7_C23	C	0.072	0.208	0.111	0.535	0.083	0.223	>20	>20	0.900	5.210	0.002	0.008	0.002	0.010
6631_V3_C10	C	0.053	0.206	>20	>20	0.081	0.322	>20	>20	>10	>10	0.197	1.110	>20	>20
6980_V0_C31	C	0.022	0.064	0.027	0.156	0.012	0.040	0.072	0.195	0.100	0.330	>20	>20	0.206	1.177
703010054_2A2	C	0.032	0.162	0.037	0.156	0.108	0.309	0.331	1.338	0.750	2.427	>20	>20	>20	>20
703010131_1E2	C	0.027	0.162	>20	>20	0.020	0.085	0.287	2.381	>10	>10	>20	>20	>20	>20
704010042_2E5	C	0.012	0.048	0.010	0.044	0.046	0.144	0.040	0.111	0.160	0.600	>20	>20	>20	>20
704810053_2B7	C	0.076	0.267	0.109	0.312	0.072	0.255	1.582	9.119	>10	>10	>20	>20	>20	>20
705010185_TF	C	0.062	0.288	>20	>20	0.444	1.236	0.050	0.147	>10	>10	>20	>20	>20	>20
706010164_1A7	C	0.035	0.175	0.021	0.121	0.036	0.106	18.871	>20	0.240	0.820	0.033	0.114	0.025	0.073
722_G4_16	C	0.193	0.653	0.209	0.947	0.091	0.320	4.086	>20	>10	>10	0.006	0.023	0.013	0.048
B005018_8_F6_3	C	0.020	0.079	0.023	0.118	0.051	0.174	0.093	0.405	0.280	1.000	>20	>20	17.366	>20
CA146_H3_3	C	>20	>20	>20	>20	0.388	1.826	0.578	1.968	>10	>10	0.013	0.047	0.007	0.021
CA240_A5_5	C	0.562	4.381	>20	>20	0.082	0.229	0.631	3.391	1.330	4.950	0.004	0.014	0.002	0.007
CA392_H2_6	C	0.037	0.091	0.102	0.406	0.186	0.628	0.036	0.134	0.180	0.810	0.086	0.319	0.901	4.695
CAP136_1_16_E6_1	C	>20	>20	1.372	>20	0.087	0.528	0.253	1.641	>10	>10	0.050	0.165	0.047	0.198
CAP174_1_06_F3_1B	C	0.084	0.742	0.370	>20	0.007	0.083	19.454	>20	0.050	0.840	0.009	0.028	0.003	0.013
CAP177_1A3	C	0.018	0.066	0.070	0.425	0.011	0.027	0.527	>20	0.150	0.500	>20	>20	0.085	0.617
CAP188_1_D1_14_REV	C	0.012	0.044	0.006	0.032	0.010	0.029	1.364	16.567	0.180	0.620	>20	>20	1.842	13.275
CAP200_B8A	C	>20	>20	>20	>20	0.066	0.182	0.142	0.441	0.480	1.760	0.025	0.070	0.006	0.016
CAP210_TA5	C	0.904	4.099	>20	>20	>20	>20	5.720	>20	>10	>10	>20	>20	7.943	>20
CAP225_1_06_A2_18	C	0.176	0.804	0.056	0.286	13.232	>20	6.174	>20	1.170	4.740	0.250	0.914	0.567	2.551
CAP244_D3	C	0.082	0.197	0.051	0.242	0.102	0.452	0.088	0.398	0.671	2.558	>20	>20	>20	>20
CAP266_2_00_E9_H6	C	0.010	0.030	0.258	3.050	0.009	0.025	>20	>20	>10	>10	>20	>20	>20	>20
CAP290_2_00_23_6	C	0.071	0.255	0.060	0.454	0.023	0.091	>20	>20	1.980	>10	7.994	>20	9.854	>20
CAP291_2_00_H2_15	C	0.060	0.176	0.063	0.246	0.130	0.416	0.095	0.410	0.580	1.560	0.083	0.276	0.024	0.075
CAP304_2_00_F6_6	C	0.026	0.100	0.032	0.119	0.009	0.027	1.282	>20	0.040	0.130	0.678	4.313	0.115	0.525
CAP307_2_00_24_1	C	2.387	>20	0.120	0.829	0.045	0.220	0.125	0.556	0.290	1.010	0.076	0.262	0.093	0.370
CAP308_2_00_E11_35	C	>20	>20	>20	>20	>20	>20	>20	>20	>10	>10	0.533	2.991	2.151	10.480
CAP323_2_00_B6_45	C	0.600	3.024	0.084	0.415	0.145	0.669	0.141	0.714	>10	>10	0.105	1.520	0.050	0.382
CAP330_2_00_F2_41	C	0.017	0.050	>20	>20	0.003	0.012	>20	>20	0.560	8.710	>20	>20	>20	>20
CAP363_2_00_10_3	C	0.704	3.788	>20	>20	0.148	0.714	0.286	1.502	2.120	>10	0.036	0.103	0.009	0.033
CAP45_G3	C	>20	>20	>20	>20	0.043	0.135	1.342	13.029	5.959	>10	>20	>20	1.356	14.585
CAP69_1_12_TA7_1	C	>20	>20	>20	>20	0.025	0.069	>20	>20	>10	>10	0.024	0.069	0.007	0.031
CE0665_F2	C	0.048	0.187	>20	>20	0.092	0.304	0.125	0.500	0.480	1.750	0.114	0.276	0.062	0.177
CE1172_H1	C	0.295	1.051	>20	>20	0.530	2.539	>20	>20	>10	>10	0.024	0.098	0.013	0.047
CE2052_G10	C	0.003	0.036	0.040	0.191	0.043	0.119	0.048	0.131	0.180	0.590	>20	>20	>20	>20
CH0694	C	0.124	0.429	0.091	0.408	0.076	0.235	0.647	5.333	>10	>10	0.246	0.839	0.261	1.140
CH505_W4_3	C	0.007	0.026	0.005	0.014	0.010	0.027	0.011	0.034	0.027	0.110	>20	>20	>20	>20
CT072_56_7	C	0.011	0.042	0.033	0.114	0.019	0.046	0.014	0.037	0.060	0.310	0.002	0.006	0.001	0.006
CT140_140_B6	C	0.142	0.495	>20	>20	0.060	0.296	0.126	0.537	>10	>10	0.069	0.300	0.008	0.031
CT184_D3_15	C	1.076	13.000	0.011	0.031	0.032	0.087	0.133	0.925	0.100	0.360	0.002	0.007	0.001	0.002
CT565_C7_48	C	2.263	9.911	9.330	>20	5.233	>20	>20	>20	>10	>10	2.970	15.079	11.892	>20

Table S5. *Neutralizing Activity Against Multiclade, Clade C, and Primary Virus Panels, Related to Figure 2 (continued)*

100 Pseudovirus Clade C Panel *(continued)*

		561_01_18		561_02_12		N6		3BNC117		VRC01		10-1074		PGT121	
Virus	Clade	IC_{50}	IC_{80}	IC_{50}	IC_{80}	IC_{50}	IC_{80}	IC_{50}	IC_{80}	IC_{50}	IC_{80}	IC_{50}	IC_{80}	IC_{50}	IC_{80}
CT810_G4_7	C	0.071	0.361	>20	>20	0.100	0.450	1.191	8.124	0.510	1.920	0.264	0.912	0.053	0.286
CT966_E1_7	C	0.057	0.234	0.177	1.006	0.038	0.156	0.200	0.702	0.560	2.940	>20	>20	7.854	>20
DU422_1	C	0.063	0.216	0.050	0.238	0.030	0.156	>20	>20	>10	>10	0.041	0.153	0.045	0.173
KO426_T78_10	C	0.056	0.146	0.097	0.313	0.136	0.409	0.141	0.428	1.089	4.167	0.060	0.185	10.178	>20
KO459_T68_4	C	0.159	0.524	0.642	5.878	0.156	0.415	0.173	0.570	1.270	5.570	0.043	0.117	0.063	0.357
KO870_C2_10	C	0.157	0.358	0.064	0.180	0.042	0.117	0.131	0.433	0.230	0.870	>20	>20	>20	>20
ME178_G6_16	C	0.176	0.600	0.122	0.559	0.119	0.520	0.425	2.100	0.400	1.380	0.018	0.055	0.099	0.574
SO032_A2_8_1	C	0.035	0.133	>20	>20	0.147	0.533	4.873	>20	1.980	>10	0.005	0.015	0.004	0.012
SO405_T24_5	C	0.073	0.225	0.292	1.090	0.279	1.274	0.330	0.872	3.410	>10	0.001	0.005	0.007	0.019
SO431_C1_1	C	0.018	0.083	0.011	0.082	0.003	0.020	0.198	5.198	0.560	3.640	>20	>20	0.025	0.183
SO607_B6_9	C	0.071	0.257	0.049	0.233	0.131	0.347	0.124	0.433	0.490	1.740	>20	>20	>20	>20
ZM106_9	C	0.011	0.035	0.071	0.296	0.026	0.093	0.076	0.277	0.254	0.714	0.008	0.042	0.003	0.016
ZM135_10A	C	1.729	13.124	>20	>20	0.144	0.430	0.056	0.205	0.899	5.016	0.066	0.331	0.897	6.359
ZM197_7	C	0.091	0.321	0.137	0.714	0.061	0.342	0.335	1.145	0.516	1.673	>20	>20	>20	>20
ZM214_15	C	0.048	0.654	0.007	0.076	0.071	0.369	0.091	0.692	0.821	3.344	0.411	2.752	0.464	2.353
ZM215_8	C	0.039	0.205	0.027	0.193	0.043	0.199	0.013	0.086	0.166	0.839	0.007	0.061	0.012	0.052
ZM233_6	C	0.042	0.159	0.438	6.876	0.066	0.210	0.191	0.901	2.527	10.823	0.021	0.348	2.311	2.673
ZM249_1	C	0.020	0.073	5.632	>20	0.019	0.074	0.041	0.186	0.073	0.272	>20	>20	3.200	>20
GeoMean of Neutralized		0.074	0.279	0.084	0.316	0.062	0.201	0.259	0.613	0.471	1.374	0.050	0.154	0.087	0.201
Breadth (%)		90.0	88.0	74.0	68.0	97.0	95.0	81.0	66.0	76.0	64.0	63.0	60.0	72.0	63.0

N6, 3BNC117, VRC01, 10-1074, and PGT121 neutralization data were derived from CATNAP.

Antibody concentration (μg/ml)	<0.1	0.1 - 0.5	0.5 - 1	1 - 2	2 - 5	5 - 10	>10
Breadth (%)	>95	90 - 95	80 - 90	70 - 80	60 - 70	50 - 60	<50

Table S5. *Neutralizing Activity Against Multiclade, Clade C, and Primary Virus Panels, Related to Figure 2 (continued)*

C 51 Outgrowth Culture-Derived Viruses

Virus	Clade	561_01_18		561_01_55		N6		3BNC117		VRC01		10-1074		PGDM1400	
		IC_{50}	IC_{80}	IC_{50}	IC_{80}	IC_{50}	IC_{80}	IC_{50}	IC_{80}	IC_{50}	IC_{80}	IC_{50}	IC_{80}	IC_{50}	IC_{80}
IDC#0355	AG	1.461	3.205	1.432	3.608	2.127	7.093	6.871	>20	10.211	>20	0.095	0.267	5.903	>20
IDC#0763	AG	0.085	0.237	0.064	0.218	0.261	0.761	0.408	1.119	0.517	1.829	0.115	0.279	4.480	19.040
IDC#0034	B	2.228	5.254	2.007	5.646	3.683	12.057	1.995	6.491	>20	>20	0.029	0.078	>20	>20
IDC#0039	B	2.474	6.289	2.989	8.410	5.398	>20	9.121	>20	>20	>20	>20	>20	>20	>20
IDC#0049	B	0.225	0.579	0.224	0.633	0.257	0.773	0.277	1.588	0.471	1.468	>20	>20	>20	>20
IDC#0052	B	0.753	2.174	0.671	2.118	0.600	3.084	1.060	4.010	2.778	12.760	0.377	0.846	1.453	3.657
IDC#0114	B	1.193	3.752	1.203	4.837	3.157	15.679	1.492	6.716	11.891	>20	<0.010	0.041	0.092	0.596
IDC#0115	B	0.160	0.682	0.210	0.717	1.317	4.956	0.382	1.737	2.459	16.411	<0.010	0.017	>20	>20
IDC#0170	B	<0.010	0.017	<0.010	0.068	0.073	0.371	0.071	0.432	0.208	0.907	1.618	10.670	>20	>20
IDC#0171	B	0.162	0.403	0.227	0.627	2.196	7.958	0.601	2.336	8.549	>20	0.293	0.730	>20	>20
IDC#0176	B	1.399	2.722	2.076	5.159	3.276	14.943	1.010	5.675	17.053	>20	0.412	1.089	0.325	1.775
IDC#0183	B	0.542	1.927	0.660	2.295	0.474	3.203	5.017	>20	2.218	>20	0.585	2.128	>20	>20
IDC#0190	B	0.054	0.198	0.081	0.272	0.564	1.546	0.287	1.220	1.877	8.138	0.100	0.377	1.504	7.405
IDC#0213	B	2.554	6.397	2.317	7.688	19.416	>20	2.667	17.200	>20	>20	0.354	0.906	0.210	6.623
IDC#0228	B	4.784	11.207	8.618	>20	7.813	>20	2.300	9.107	>20	>20	1.774	15.570	>20	>20
IDC#0230	B	0.210	1.208	0.510	2.413	1.583	7.546	1.334	12.872	6.322	>20	0.060	0.915	>20	>20
IDC#0233	B	5.477	>20	2.562	8.446	>20	>20	1.606	8.980	>20	>20	5.466	>20	>20	>20
IDC#0246	B	2.368	6.492	1.925	6.026	12.309	>20	3.589	14.560	>20	>20	1.447	3.726	1.104	4.957
IDC#0261	B	0.604	1.809	0.995	2.619	0.585	2.832	0.503	3.235	1.210	7.087	2.043	8.232	0.401	1.938
IDC#0338	B	0.127	0.376	0.217	0.614	0.514	1.960	0.209	0.853	2.049	9.573	0.044	0.170	18.149	>20
IDC#0362	B	0.208	0.608	0.340	0.856	0.373	1.107	0.087	0.349	0.719	2.685	0.267	>20	1.229	>20
IDC#0365	B	0.315	0.838	0.396	1.021	0.673	2.012	0.440	1.481	1.938	6.335	<0.010	0.028	>20	>20
IDC#0403	B	6.062	15.186	9.595	>20	2.213	9.314	>20	>20	>20	>20	0.896	3.040	6.140	>20
IDC#0428	B	2.926	9.014	3.945	10.286	1.190	2.930	2.290	6.772	2.690	9.648	0.477	7.845	2.659	>20
IDC#0438	B	0.462	1.225	0.470	1.219	3.592	9.314	1.676	5.611	6.402	>20	0.024	0.120	>20	>20
IDC#0440	B	2.248	5.670	2.005	5.270	3.678	16.480	1.649	6.151	12.071	>20	>20	>20	0.289	1.750
IDC#0449	B	0.195	0.482	0.200	0.526	1.100	5.061	0.282	1.589	3.544	17.233	0.046	0.125	0.250	0.807
IDC#0453	B	1.626	4.447	1.668	4.049	2.117	6.169	1.467	4.291	10.605	>20	0.415	1.033	0.337	>20
IDC#0460	B	0.141	0.433	0.174	0.640	0.461	1.689	>20	>20	>20	>20	0.417	1.560	>20	>20
IDC#0464	B	0.202	1.016	0.337	1.201	0.236	1.330	0.495	2.536	1.215	5.483	2.762	>20	<0.010	>20
IDC#0467	B	0.205	0.696	0.283	0.997	0.381	1.358	0.530	2.064	3.242	14.350	0.109	0.426	0.317	1.900
IDC#0480	B	0.191	0.668	0.369	0.865	0.896	2.158	0.872	2.571	2.050	6.449	0.081	0.250	0.103	0.431
IDC#0527	B	>20	>20	>20	>20	>20	>20	>20	>20	>20	>20	>20	>20	>20	>20
IDC#0535	B	0.484	1.495	0.648	2.162	0.965	2.671	0.622	1.530	1.171	5.692	0.740	2.123	0.606	18.190
IDC#0540	B	1.331	3.092	1.729	4.460	2.878	9.422	1.355	3.861	7.124	>20	0.552	1.767	1.305	6.870
IDC#0541	B	0.566	2.039	1.118	2.458	8.265	19.597	3.331	7.394	19.438	>20	0.293	0.727	>20	>20
IDC#0548	B	0.163	0.547	0.282	0.764	1.696	4.501	0.985	2.541	7.670	>20	0.079	0.305	<0.010	<0.010
IDC#0551	B	0.995	2.497	1.333	3.006	1.153	2.923	0.230	0.694	1.550	4.865	2.635	6.679	>20	>20
IDC#0578	B	0.119	0.277	0.182	0.664	0.266	0.748	>20	>20	0.491	2.094	0.736	1.973	1.527	8.611
IDC#0579	B	0.185	0.525	0.315	0.917	0.766	2.150	>20	>20	>20	>20	2.072	>20	1.516	>20
IDC#0615	B	0.558	1.654	1.201	2.309	2.912	7.215	0.415	1.355	4.980	17.759	>20	>20	2.405	6.679
IDC#0648	B	0.554	1.762	0.958	2.501	1.340	4.197	0.423	1.172	1.578	8.994	0.078	0.334	1.952	7.911
IDC#0683	B	4.107	11.123	5.080	14.716	5.334	18.067	1.873	6.689	11.529	>20	>20	>20	0.720	2.661
IDC#0758	B	0.291	0.756	0.656	2.490	0.457	1.361	>20	>20	>20	>20	5.610	>20	3.726	13.729
IDC#0761	B	0.471	1.213	0.574	1.932	0.674	2.181	1.554	7.146	1.259	5.396	>20	>20	0.119	13.561
IDC#0779	B	0.888	8.496	1.049	4.104	11.159	17.434	>20	>20	2.689	10.735	>20	>20	0.462	1.992
IDC#0787	B	0.051	0.203	0.075	0.273	0.293	1.016	0.112	0.383	1.292	3.901	0.066	0.248	>20	>20
IDC#0799	B	1.605	5.506	4.690	13.625	0.659	1.925	1.482	4.547	3.819	>20	8.033	18.912	>20	>20
IDC#0801	B	2.457	6.553	2.534	10.521	1.973	5.450	1.374	3.889	5.114	>20	>20	>20	14.317	>20
IDC#0782	C	4.684	14.871	>20	>20	3.520	9.252	>20	>20	>20	>20	12.929	>20	17.801	>20
IDC#0508	D	>20	>20	>20	>20	>20	>20	>20	>20	>20	>20	>20	>20	>20	>20
GeoMean of Neutralized		0.555	1.568	0.698	1.899	1.319	3.659	0.879	2.908	2.872	6.018	0.313	0.752	0.816	2.955
Breadth (%)		96.1	94.1	94.1	90.2	94.1	86.3	82.4	76.5	76.5	45.1	80.4	68.6	62.7	43.1

Antibody concentration (μg/ml)	<0.1	0.1 - 0.5	0.5 - 1	1 - 2	2 - 5	5 - 10	>10
Breadth (%)	>95	90 - 95	80 - 90	70 - 80	60 - 70	50 - 60	<50
