# Supplementary material for: Restriction of HIV-1 Escape by a Highly Broad and Potent Neutralizing Antibody
Source: Cell. 2020 Feb 6;180(3):471–489.e22. doi: 10.1016/j.cell.2020.01.010 (PMC7042716; doi:10.1016/j.cell.2020.01.010)
Supplement: Table S6. Cryo-EM Data Collection, Refinement, and Validation Statistics, Related to Figure 3 [file mmc6.pdf]

Table S6. *Cryo-EM Data Collection, Refinement, and Validation Statistics, Related to Figure 3*

	**1-18–BG505–10-1074** (EMDB-20739) (PDB 6UDJ)	**1-55–RC1–10-1074** (EMDB-20740) (PDB 6UDK)
**Data collection and processing**		
Magnification	130,000x	73,000x
Voltage (kV)	300	200
Microscope	Titan Krios	Talos Arctica
Electron exposure (e–/Å^2)	60	39.1
Defocus range (μm)	1 - 2.6	1 - 3.4
Pixel size (Å)	1.057	1.40
Symmetry imposed	C3	C3
Initial particle images (no.)	352,598	160,254
Final particle images (no.)	230,924	110,126
Map resolution (Å)	2.5	3.9
FSC threshold	0.143	0.143
**Refinement**		
Initial model used (PDB code)	ab initio	5T3Z
Model resolution (Å)	2.5	3.9
FSC threshold	0.143	0.143
Map sharpening *B* factor (Å^2)	-71.0	-272.3
Model composition		
Non-hydrogen atoms (no.)	26,592	25,724
Protein residues (no.)	3,090	3,129
Ligands	BMA: 27<br>NAG: 105<br>MAN: 45	BMA: 8<br>NAG: 57<br>MAN: 23
B factors		
Protein (Å^2)	42.53	42.31
Ligand (Å^2)	62.19	48.20
R.m.s. deviations		
Bond lengths (Å)	0.010	0.004
Bond angles (°)	1.071	0.809
Validation		
MolProbity score	1.39	1.62
Clashscore	3.07	4.88
Poor rotamers (%)	0.41	0.52
Ramachandran plot		
Favored (%)	95.83	94.71
Allowed (%)	4.11	5.22
Disallowed (%)	0.07	0.07
