# Supplementary material for: Restriction of HIV-1 Escape by a Highly Broad and Potent Neutralizing Antibody
Source: Cell. 2020 Feb 6;180(3):471–489.e22. doi: 10.1016/j.cell.2020.01.010 (PMC7042716; doi:10.1016/j.cell.2020.01.010)
Supplement: Table S7. Plasma SGS-Derived env Sequences Obtained from HIV-1YU2-Infected Humanized Mice and Antibody Sensitivity, Related to Figures 6 and 7 [file mmc7.pdf]

Table S7. *Plasma SGS-Derived env Sequences Obtained from HIV-1$_{YU2}$-Infected Humanized Mice and Antibody Sensitivity, Related to Figures 6 and 7*

A 3BNC117 Treatment Group

Sensitivity

ID	Day	Virus	1-18 IC50	1-18 MPI	3BNC117 IC50	3BNC117 MPI	VRC01 IC50	VRC01 MPI
1021	-1	01-40	n/a	n/a	n/a	n/a	n/a	n/a
		06-32	n/a	n/a	n/a	n/a	n/a	n/a
		06-33	n/a	n/a	n/a	n/a	n/a	n/a
		06-35	n/a	n/a	n/a	n/a	n/a	n/a
		06-43	n/a	n/a	n/a	n/a	n/a	n/a
		06-46	n/a	n/a	n/a	n/a	n/a	n/a
		06-47	n/a	n/a	n/a	n/a	n/a	n/a
	28	03-31	n/a	n/a	n/a	n/a	n/a	n/a
		03-36	n/a	n/a	n/a	n/a	n/a	n/a
		03-42	<0.01	100	0.015	63	0.270	70
		03-46	n/a	n/a	n/a	n/a	n/a	n/a
		08-C04	n/a	n/a	n/a	n/a	n/a	n/a
		08-C12	<0.01	75	0.069	72	0.023	79
	114	21-A10	n/a	n/a	n/a	n/a	n/a	n/a
		21-B07	n/a	n/a	n/a	n/a	n/a	n/a
		29-A03	n/a	n/a	n/a	n/a	n/a	n/a
		29-A12	<0.01	100	>25	28	>25	26
1135	-1	02-34	<0.01	100	<0.01	100	<0.01	100
		07-30	n/a	n/a	n/a	n/a	n/a	n/a
		07-31	n/a	n/a	n/a	n/a	n/a	n/a
		07-34	n/a	n/a	n/a	n/a	n/a	n/a
		07-44	n/a	n/a	n/a	n/a	n/a	n/a
		07-47	n/a	n/a	n/a	n/a	n/a	n/a
	28	04-26	<0.01	100	0.026	93	0.017	88
		04-41	n/a	n/a	n/a	n/a	n/a	n/a
		04-42	n/a	n/a	n/a	n/a	n/a	n/a
		04-44	<0.01	100	>25	30	0.102	81
		09-D2	<0.01	99	>25	35	0.285	84
		09-D10	n/a	n/a	n/a	n/a	n/a	n/a
		28-E12	n/a	n/a	n/a	n/a	n/a	n/a
1140	-1	11-G02	n/a	n/a	n/a	n/a	n/a	n/a
		11-G12	n/a	n/a	n/a	n/a	n/a	n/a
		11-H02	n/a	n/a	n/a	n/a	n/a	n/a
		11-H05	n/a	n/a	n/a	n/a	n/a	n/a
		11-H06	n/a	n/a	n/a	n/a	n/a	n/a
		11-H07	n/a	n/a	n/a	n/a	n/a	n/a
		11-H11	n/a	n/a	n/a	n/a	n/a	n/a
		16-G01	n/a	n/a	n/a	n/a	n/a	n/a
		16-G03	n/a	n/a	n/a	n/a	n/a	n/a
		16-G04	n/a	n/a	n/a	n/a	n/a	n/a
		16-G11	n/a	n/a	n/a	n/a	n/a	n/a
	28	14-C04	n/a	n/a	n/a	n/a	n/a	n/a
		19-C02	n/a	n/a	n/a	n/a	n/a	n/a
		19-C03	n/a	n/a	n/a	n/a	n/a	n/a
		19-C05	n/a	n/a	n/a	n/a	n/a	n/a
		19-C06	<0.01	100	0.019	75	0.076	83
		19-C09	<0.01	100	>25	47	0.023	89
		19-C10	n/a	n/a	n/a	n/a	n/a	n/a
		19-C11	n/a	n/a	n/a	n/a	n/a	n/a
		19-D02	n/a	n/a	n/a	n/a	n/a	n/a
		19-D03	n/a	n/a	n/a	n/a	n/a	n/a
	114	21-C05	n/a	n/a	n/a	n/a	n/a	n/a
		21-C07	0.012	100	>25	42	0.100	88
		21-C10	n/a	n/a	n/a	n/a	n/a	n/a
		21-D07	<0.01	100	0.068	69	0.028	83

ID	Day	Virus	V1 loop / V2 loop (132–209)	Loop D (274–284)	V3 loop (298–331)	CD4 binding loop (363–375)	V4 loop / β20/β21 (385–438)	β23 / V5/β24 (450–471)
			TDLRNATNTTSSSWETMEKGEIKNCSFNITTSIRDKVQKEYALFYNLDVVPIDNASYRLISCNTSVITQACPKVS	SENFTNNAKTI	RPNNNTRKSINIGPGRALYTTGEIIGDIRQAHC	PSSGGDPEIVTHS	CNSTQLFTWNDTRKLNNTGRNITLPCRIKQIINMWQEVGKAMYAPP	TGLLLTRDGGKDTNGTEIFRPG
1021	-1	01-40	....	....	....	....	.............R................................	....
		06-32	....	....	....	....	....	....
		06-33	....	....	....	....	....	....
		06-35	....	....	....	....	....	....
		06-43	....	....	....	....	....	....
		06-46	....	....	....	....	....	....
		06-47	....	....	......-..........................	....	....	....
	28	03-31	....	....IK.....	....	....	....	....
		03-36	....	....IK.....	....	....	....	....
		03-42	....	....	....	....	....	.........D..--.......E
		03-46	.............................---...	.......T...	........................--.......	....	....	....
		08-C04	....	.......D...	....	....	....	......K...............
		08-C12	....	....	....	....	....	.........D...........E
	114	21-A10	....K...................................	........N..	....	............R	....	....
		21-B07	....K...................................	.......T...	....	....	....	..............EI......
		29-A03	....	....IK.....	....	....	....	....
		29-A12	.............................---...	.....K.....	....	....	....	....
1135	-1	02-34	....	....	....	....	....	....
		07-30	....	....	....	....	....	......K...............
		07-31	....	....	....	........M....	....	....
		07-34	....	....	....	....	....	....
		07-44	....	....	....	....	....	....
		07-47	....	....	....	....	....	....
	28	04-26	....	....	....	....	....	.........D....E.......
		04-41	....	........E..	....	....	....	.........D....E.......
		04-42	....	........E..	....	....	....	.........D....E.......
		04-44	....	........E..	....	....	....	.........D....E.......
		09-D2	....	........E..	....	....	....	.........D....E.......
		09-D10	....	....	....	....	....	.........D....E.......
		28-E12	....	....	....	....	....	.........D....E.......
1140	-1	11-G02	....	....	....	....	....	....
		11-G12	....	....	...................A.............	....	....	.........D............
		11-H02	....	....	....	....	....	....
		11-H05	....	....	....	....	....	....
		11-H06	....	....	................T................	....	....	.........D............
		11-H07	....	....	....	....	....	....
		11-H11	....	....	....	....	....	....
		16-G01	....	....	....	....	....	.........D............
		16-G03	....	....	....	....	....	....
		16-G04	....	....	....	....	....	................K.....
		16-G11	....	....	....	....	............K.................................	....
	28	14-C04	....	....	....	....	...................K..........................	.........D............
		19-C02	....	....	....	....	....................................K.........	.........D............
		19-C03	..............K..	....	....	....	....................................K.........	.........D............
		19-C05	....	....	....	....	....................................K.........	.........D............
		19-C06	....	....	....	....	....	.........D....E.......
		19-C09	....	........E..	....	....	....	.........D............
		19-C10	....	........E..	....	....	....................................K.........	.........D............
		19-C11	....	....	....	....	....................................K.........	.........D............
		19-D02	....	........E..	....	....	....	.........D............
		19-D03	....	....	....	....	....................................K.........	.........D............
	114	21-C05	....	........E..	.........................E.......	....	....	.........D............
		21-C07	....	........E..	....	....	....	.........D............
		21-C10	....	....	....	....	....	.........D............
		21-D07	....	....	....	....	....	.........D............

Table S7. *Plasma SGS-Derived env Sequences Obtained from HIV-1$_{YU2}$-Infected Humanized Mice and Antibody Sensitivity, Related to Figures 6 and 7 (continued)*

3BNC117 Treatment Group *(continued)*

ID	Day	Virus	Sensitivity: 1-18 IC_{50}	1-18 MPI	3BNC117 IC_{50}	3BNC117 MPI	VRC01 IC_{50}	VRC01 MPI
1211	-1	11-E01	n/a	n/a	n/a	n/a	n/a	n/a
		11-E08	n/a	n/a	n/a	n/a	n/a	n/a
		16-E04	n/a	n/a	n/a	n/a	n/a	n/a
		16-E12	n/a	n/a	n/a	n/a	n/a	n/a
		16-F10	n/a	n/a	n/a	n/a	n/a	n/a
	28	14-B07	<0.01	94	>25	36	0.033	83
		19-A03	<0.01	100	2.314	74	0.029	100
		19-A04	n/a	n/a	n/a	n/a	n/a	n/a
		19-A07	n/a	n/a	n/a	n/a	n/a	n/a
		19-A08	0.017	100	0.114	98	<0.01	99
		19-B02	n/a	n/a	n/a	n/a	n/a	n/a
		19-B03	<0.01	100	12.428	56	0.040	94
		19-B06	n/a	n/a	n/a	n/a	n/a	n/a
		19-B07	<0.01	100	13.957	58	0.154	91
		19-B09	n/a	n/a	n/a	n/a	n/a	n/a
		19-B10	n/a	n/a	n/a	n/a	n/a	n/a
1249	114	29-E02	<0.01	100	9.854	65	10.916	66
		29-F06	n/a	n/a	n/a	n/a	n/a	n/a
		29-F09	n/a	n/a	n/a	n/a	n/a	n/a

ID	Day	Virus	V1 loop / V2 loop (132–209)	Loop D (274–284)	V3 loop (298–331)	CD4 binding loop (363–375)	V4 loop / β20/β21 (385–438)	β23 / V5/β24 (450–471)
			TDLRNATNTTSSSWETMEKGEIKNCSFNITTSIRDKVQKEYALFYNLDVVPIDNASYRLISCNTSVITQACPKVS	SENFTNNAKTI	RPNNNTRKSINIGPGRALYTTGEIIGDIRQAHC	PSSGGDPEIVTHS	CNSTQLFTWNDTRKLNNTGRNITLPCRIKQIINMWQEVGKAMYAPP	TGLLLTRDGGKDTNGTEIFRPG
1211	-1	11-E01	....	....	....	..L..........	....	....
		11-E08	....	....	....	..L..........	....	....
		16-E04	....	....	....	.P...........	....	.........E............
		16-E12	....	....	....	...E.....L...	....	....
		16-F10	....	....	....	....	....	.........E............
	28	14-B07	....	.......D...	....	....	....	.........E............
		19-A03	....	.......D...	....	....	....	.........E............
		19-A04	....	....	....	....	....	.........E............
		19-A07	....	....	....	....	....	.........E............
		19-A08	....	....	....	....	......V.......................................	.........E............
		19-B02	....	........R..	....	....	.T..	.........E............
		19-B03	...........R...	........R..	....	....	.T..	.........E............
		19-B06	....	....	....	....	....	.........E............
		19-B07	....	.......D...	....	....	....	.........E............
		19-B09	................T..	........R..	....	....	....	.........E............
		19-B10	....	........R..	....	....	....	.........E............
1249	114	29-E02	................................R..	....	....	....	....	........D.............
		29-F06	................................R..	....	....	....	....	........D.............
		29-F09	................................R..	....	....	....	....	........D.............

Table S7. *Plasma SGS-Derived env Sequences Obtained from HIV-1$_{YU2}$-Infected Humanized Mice and Antibody Sensitivity, Related to Figures 6 and 7 (continued)*

B VRC01 Treatment Group

ID	Day	Virus	Sensitivity 1-18 IC_{50}	1-18 MPI	3BNC117 IC_{50}	3BNC117 MPI	VRC01 IC_{50}	VRC01 MPI
1050	-1	12-A05	n/a	n/a	n/a	n/a	n/a	n/a
		12-B11	n/a	n/a	n/a	n/a	n/a	n/a
		17-A10	n/a	n/a	n/a	n/a	n/a	n/a
		17-A11	<0.01	100	<0.01	100	0.043	100
		17-B09	n/a	n/a	n/a	n/a	n/a	n/a
	28	14-E03	n/a	n/a	n/a	n/a	n/a	n/a
		14-E06	<0.01	100	0.217	74	0.218	63
1130	-1	12-C07	n/a	n/a	n/a	n/a	n/a	n/a
		12-D01	n/a	n/a	n/a	n/a	n/a	n/a
		12-D05	n/a	n/a	n/a	n/a	n/a	n/a
		17-C07	n/a	n/a	n/a	n/a	n/a	n/a
		17-D08	n/a	n/a	n/a	n/a	n/a	n/a
	28	14-G02	<0.01	81	0.025	77	0.203	81
		14-G05	n/a	n/a	n/a	n/a	n/a	n/a
		14-G11	n/a	n/a	n/a	n/a	n/a	n/a
		19-H01	n/a	n/a	n/a	n/a	n/a	n/a
		19-H02	n/a	n/a	n/a	n/a	n/a	n/a
		19-H06	n/a	n/a	n/a	n/a	n/a	n/a
		19-H10	<0.01	95	1.243	67	>25	28
	114	25-C01	n/a	n/a	n/a	n/a	n/a	n/a
		25-C02	n/a	n/a	n/a	n/a	n/a	n/a
		25-C11	n/a	n/a	n/a	n/a	n/a	n/a
		25-D07	n/a	n/a	n/a	n/a	n/a	n/a
		33-C08	n/a	n/a	n/a	n/a	n/a	n/a
		33-D04	<0.01	100	0.118	78	0.180	78
		33-D08	n/a	n/a	n/a	n/a	n/a	n/a
1161	-1	02-82	n/a	n/a	n/a	n/a	n/a	n/a
		02-84	n/a	n/a	n/a	n/a	n/a	n/a
	28	04-73	n/a	n/a	n/a	n/a	n/a	n/a
		04-93	<0.01	100	0.412	80	9.639	74
		09-G04	n/a	n/a	n/a	n/a	n/a	n/a
		09-G07	n/a	n/a	n/a	n/a	n/a	n/a
		09-G09	n/a	n/a	n/a	n/a	n/a	n/a
		09-H01	n/a	n/a	n/a	n/a	n/a	n/a
		09-H09	n/a	n/a	n/a	n/a	n/a	n/a
	114	25-A01	<0.01	92	19.359	55	5.861	56
		25-A12	0.027	100	0.238	66	21.735	52
		33-A08	n/a	n/a	n/a	n/a	n/a	n/a
		33-A11	n/a	n/a	n/a	n/a	n/a	n/a
		33-B02	n/a	n/a	n/a	n/a	n/a	n/a
1210	-1	02-02	n/a	n/a	n/a	n/a	n/a	n/a
		02-04	n/a	n/a	n/a	n/a	n/a	n/a
		02-06	n/a	n/a	n/a	n/a	n/a	n/a
		02-07	n/a	n/a	n/a	n/a	n/a	n/a
		02-08	n/a	n/a	n/a	n/a	n/a	n/a
		02-12	n/a	n/a	n/a	n/a	n/a	n/a
		02-14	n/a	n/a	n/a	n/a	n/a	n/a
		02-20	n/a	n/a	n/a	n/a	n/a	n/a
		02-21	n/a	n/a	n/a	n/a	n/a	n/a
	28	04-01	n/a	n/a	n/a	n/a	n/a	n/a
		04-08	n/a	n/a	n/a	n/a	n/a	n/a
		04-23	n/a	n/a	n/a	n/a	n/a	n/a
		09-A08	<0.01	100	22.647	52	>25	45
		09-A11	0.022	100	>25	38	25.000	50
		09-A12	n/a	n/a	n/a	n/a	n/a	n/a
		09-B06	n/a	n/a	n/a	n/a	n/a	n/a
	114	24-G10	n/a	n/a	n/a	n/a	n/a	n/a
		24-H04	n/a	n/a	n/a	n/a	n/a	n/a
		32-G04	<0.01	100	22.820	57	>25	37
		32-G08	<0.01	100	<0.01	100	0.015	100
		32-H01	n/a	n/a	n/a	n/a	n/a	n/a
		32-H07	n/a	n/a	n/a	n/a	n/a	n/a
		32-H12	n/a	n/a	n/a	n/a	n/a	n/a

Virus	V1 loop / V2 loop (132–209)	Loop D (274–284)	V3 loop (298–331)	CD4 binding loop (363–375)	V4 loop / β20/β21 (385–438)	β23 / V5/β24 (450–471)
	TDLRNATNTTSSSWETMEKGEIKNCSFNITTSIRDKVQKEYALFYNLDVVPIDNASYRLISCNTSVITQACPKVS	SENFTNNAKTI	RPNNNTRKSINIGPGRALYTTGEIIGDIRQAHC	PSSGGDPEIVTHS	CNSTQLFTWNDTRKLNNTGRNITLPCRIKQIINMWQEVGKAMYAPP	TGLLLTRDGGKDTNGTEIFRPG
12-A05	....	....	....	....	....	.........V............
12-B11	....	....	....	....	....	....
17-A10	....	....	....	....	....	....
17-A11	....	....	....	....	....	.....................R
17-B09	....	....	....	....	....	.........D............
14-E03	....	......D....	....	....	....	.........V............
14-E06	....E......................	....	....	....	...................K..........................	.........VE...........
12-C07	....	....	....	....	....	....
12-D01	...........................S...	....	....	....	....................................K.........	....
12-D05	....	....	....	....	....	....
17-C07	....	....	....	....	....	....
17-D08	...........................S...	....	....	....	....................................K.........	....
14-G02	...........................S...	....	....	....	....................................K.........	.........D.....N......
14-G05	....	....	....	....	....................................K.........	.........D.....N.....E
14-G11	....	......D....	....	....	....................................K.........	.........D.....N......
19-H01	....	....	....	....	....................................K.........	.........D.....N......
19-H02	....	....	....	....	....................................K.........	.........D.....N......
19-H06	.............................N...	......D....	....	....	....	.........D.....N......
19-H10	....	......D....	....	....	....	......K...............
25-C01	...........................S...	....	......................A..........	....	....................................K.........	.........D.....N......
25-C02	...........................S...	....	....	....	....	.........D.....N......
25-C11	....	....	....	....	....	.........D.....N......
25-D07	.........................F.S...	....	....	....	....................................K.........	.........D.....N......
33-C08	...................E...	....	Y.TTI............................	....	....................................K.........	.........D.....NK.....
33-D04	....	....	....	....	....................................K.........	.........D.....N......
33-D08	...........................S...	....	....	....	....................................K.........	.........D.....N......
02-82	....	....	....	........M....	....	....
02-84	....	....	....	....	.................................I............	....
04-73	................I..	.....S.....	....	....	....	.........D............
04-93	....	......D....	....	....	....	....
09-G04	....	......D....	....	....	....	....
09-G07	....	....	....	....	....	....
09-G09	....	....	....	....	....	....
09-H01	....	......D....	....	....	....	....
09-H09	....	......D....	....	....	....	....
25-A01	.........P...........V...	......D....	....	....	....	....
25-A12	.....................V...	.......T...	....	....	....	....
33-A08	....	......D....	....	....	.K..	....
33-A11	.....................V...	.......T...	....	....	....	....
33-B02	....	.......T...	....	....	....	....
02-02	.................K...	....	....	....	....	....
02-04	....	....	....	....	....	....
02-06	....	....	....	....	....	....
02-07	....	....	....	....	....	....
02-08	....	....	....	....	....	....
02-12	....	....	....	....	....	.................V....
02-14	....	....	....	....	....	....
02-20	....	....	....	....	....	....
02-21	....	....	....	....	....	....
04-01	....	.....Y.....	....	....	....	....
04-08	....	.....Y.....	....	....	....	....
04-23	....	.....Y.....	....	....	............K.................................	....
09-A08	....	.....Y.....	........G........................	....	....	....
09-A11	....	....A..T...	....	....	....	....
09-A12	....	....A..T...	....	....	....	....
09-B06	....	....A..T...	....	....	....	....
24-G10	....K...................................	....A..T...	....	....	....	....
24-H04	....	....A......	....	....	....	....
32-G04	....K...................................	....A..T...	....	....	....	....
32-G08	....	....A......	....	....	....	....
32-H01	....K...................................	....A..T...	....	....	....	...........N..........
32-H07	....	....A..T...	....	....	....	....
32-H12	....	....A..T...	....	....	....	....

Table S7. *Plasma SGS-Derived env Sequences Obtained from HIV-1$_{YU2}$-Infected Humanized Mice and Antibody Sensitivity, Related to Figures 6 and 7 (continued)*

C 3BNC117 + VRC01 Treatment Group

ID	Day	Virus	1-18 IC$_{50}$	1-18 MPI	3BNC117 IC$_{50}$	3BNC117 MPI	VRC01 IC$_{50}$	VRC01 MPI	V1 loop / V2 loop (132–209)	Loop D (274–284)	V3 loop (298–331)	CD4 binding loop (363–375)	V4 loop / β20/β21 (385–438)	β23 / V5/β24 (450–471)
									TDLRNATNTTSSSWETMEKGEIKNCSFNITTSIRDKVQKEYALFYNLDVVPIDNASYRLISCNTSVITQACPKVS	SENFTNNAKTI	RPNNNTRKSINIGPGRALYTTGEIIGDIRQAHC	PSSGGDPEIVTHS	CNSTQLFTWNDTRKLNNTGRNITLPCRIKQIINMWQEVGKAMYAPP	TGLLLTRDGGKDTNGTEIFRPG
940	-1	01-22	n/a	n/a	n/a	n/a	n/a	n/a	....	....	....	....	....	....
940	-1	06-05	n/a	n/a	n/a	n/a	n/a	n/a	....	....	....	........M....	....	....
940	-1	06-06	n/a	n/a	n/a	n/a	n/a	n/a	....	....	....	....	....	....
940	-1	06-08	n/a	n/a	n/a	n/a	n/a	n/a	....	....	....	....	.................................I............	....
940	-1	06-09	n/a	n/a	n/a	n/a	n/a	n/a	....	....	....	....	....	....................L.
940	-1	06-10	n/a	n/a	n/a	n/a	n/a	n/a	....	....	....	....	....	....
940	-1	06-11	n/a	n/a	n/a	n/a	n/a	n/a	....	....	....	....	---...	.........D............
940	-1	06-12	n/a	n/a	n/a	n/a	n/a	n/a	....	....	....	....	....	....
940	-1	06-15	n/a	n/a	n/a	n/a	n/a	n/a	....	....	....	....	....	....
940	-1	06-16	n/a	n/a	n/a	n/a	n/a	n/a	....	....	....	....	....	....
940	-1	06-17	n/a	n/a	n/a	n/a	n/a	n/a	....	....	....	....	....	....
940	-1	06-18	n/a	n/a	n/a	n/a	n/a	n/a	....	....	....	....	....	...........G..........
940	-1	06-19	n/a	n/a	n/a	n/a	n/a	n/a	....	....	....	....	....	................K.....
940	28	03-18	n/a	n/a	n/a	n/a	n/a	n/a	....	.....K.....	....	....	..........N...................................	....
940	28	08-A07	n/a	n/a	n/a	n/a	n/a	n/a	....	.....K.....	....	....	....	.........D............
940	28	08-A11	<0.01	100	>25	48	2.748	70	....	F..........	....	....	....	.........D............
940	28	08-A12	<0.01	100	>25	32	>25	31	....	.....K.....	....	....	....	....
940	28	08-B04	<0.01	94	0.174	79	0.126	81	....T.....	....	....	....	....	.........D....E.......
940	28	08-B09	<0.01	96	>25	32	>25	31	....I..........................	.....K.....	....	....	....................K.........................	.........D............
940	28	08-B11	n/a	n/a	n/a	n/a	n/a	n/a	.............................N...	F..........	....	....	....	.........D............
940	114	23-G09	n/a	n/a	n/a	n/a	n/a	n/a	....	....	....	....	................................K.............	....
940	114	23-H11	<0.01	100	<0.01	99	0.033	100	....	...L.......	....	....	................................K.............	....
940	114	31-G10	n/a	n/a	n/a	n/a	n/a	n/a	.............................---....................................K......	.......T...	...S.............................	....	....	....
1087	-1	01-95	n/a	n/a	n/a	n/a	n/a	n/a	....	....	....	....	....	....
1087	-1	06-85	n/a	n/a	n/a	n/a	n/a	n/a	....	....	.........................E.......	....	...................K..........................	....
1087	-1	26-C06	n/a	n/a	n/a	n/a	n/a	n/a	....	....	....	....	....	....
1087	-1	26-D07	n/a	n/a	n/a	n/a	n/a	n/a	....	....	....	....	....	....
1087	28	03-84	n/a	n/a	n/a	n/a	n/a	n/a	....	......D....	....	....	....	.........D............
1087	28	08-G01	n/a	n/a	n/a	n/a	n/a	n/a	....	....	....	....	....	.........D............
1087	28	08-G10	<0.01	86	0.029	85	0.087	86	....N......................	F..........	....	....	..........N...................................	.........D............
1087	28	08-H05	n/a	n/a	n/a	n/a	n/a	n/a	....	....	....	....	....	.........D............
1087	28	08-H06	<0.01	100	>25	41	0.053	87	....	.......D...	....	....	....	.........D............
1087	28	08-H09	n/a	n/a	n/a	n/a	n/a	n/a	....	F..........	....	....	....	.........D............
1087	28	08-H12	<0.01	100	>25	46	0.063	80	....	.......D...	....	....	....	.........D............
1087	114	32-A01	n/a	n/a	n/a	n/a	n/a	n/a	....	....	....	....	....	.........D............
1087	114	32-A09	<0.01	100	0.012	100	0.022	100	....	....	.................W...............	....	....	.........D............
1087	114	32-A10	<0.01	100	0.015	100	0.079	92	....	....	....	....	......................I.......................	.........D............
1087	114	32-B08	n/a	n/a	n/a	n/a	n/a	n/a	....	....	....	....	....	.........D............
1087	114	32-B11	<0.01	99	17.500	57	17.160	59	....	F..........	....	....	....	.........D............
1186	-1	16-A06	n/a	n/a	n/a	n/a	n/a	n/a	....	....	....	........M....	..............S...............................	....
1186	-1	16-A09	<0.01	100	<0.01	100	0.058	100	....	....	....	....	....	.....................R
1186	-1	16-A10	n/a	n/a	n/a	n/a	n/a	n/a	....	....	....	....	....	.....................R
1186	-1	16-A12	n/a	n/a	n/a	n/a	n/a	n/a	....	....	....	........M....	....	....
1186	-1	16-B01	n/a	n/a	n/a	n/a	n/a	n/a	....	....	....	........M....	....	....
1186	28	13-E10	0.022	99	0.213	73	0.355	70	....	.......T...	....	....	....	....
1186	28	13-E11	n/a	n/a	n/a	n/a	n/a	n/a	....	....	....	....	....	.........D............
1186	28	13-F08	n/a	n/a	n/a	n/a	n/a	n/a	....	.......T...	....	....	....	....
1186	28	26-E07	n/a	n/a	n/a	n/a	n/a	n/a	....	....	....	....	....	....
1186	28	26-E10	<0.01	100	0.014	97	0.044	92	....	....	....	....	....	.........D............
1186	28	26-F01	<0.01	100	5.024	67	16.398	59	....	....	....	....	....	.........DE...........
1186	28	26-F02	<0.01	93	0.327	68	0.249	62	....	.......T...	....	....	....	....
1186	28	26-F10	n/a	n/a	n/a	n/a	n/a	n/a	....	.......T...	....	....	....	....
1186	114	24-C06	n/a	n/a	n/a	n/a	n/a	n/a	....	....	....	....	....	.........D............
1186	114	32-C05	n/a	n/a	n/a	n/a	n/a	n/a	....	....	....	....	....	.........D...........E
1186	114	32-C10	n/a	n/a	n/a	n/a	n/a	n/a	....	....	....	....	....	....
1186	114	32-D04	n/a	n/a	n/a	n/a	n/a	n/a	....	.......T...	....	....	....	....
1186	114	32-D07	n/a	n/a	n/a	n/a	n/a	n/a	....I.	....	....	....	....R......	.........D............
1186	114	32-D09	n/a	n/a	n/a	n/a	n/a	n/a	....N...................................	.......T...	....................A............	....	....	....

Table S7. *Plasma SGS-Derived env Sequences Obtained from HIV-1$_{YU2}$-Infected Humanized Mice and Antibody Sensitivity, Related to Figures 6 and 7 (continued)*

3BNC117 + VRC01 Treatment Group *(continued)*

ID	Day	Virus	Sensitivity 1-18 IC_{50}	1-18 MPI	3BNC117 IC_{50}	3BNC117 MPI	VRC01 IC_{50}	VRC01 MPI	V1 loop / V2 loop (132–209)	Loop D (274–284)	V3 loop (298–331)	CD4 binding loop (363–375)	V4 loop / β20/β21 (385–438)	β23 / V5/β24 (450–471)
									TDLRNATNTTSSSWETMEKGEIKNCSFNITTSIRDKVQKEYALFYNLDVVPIDNASYRLISCNTSVITQACPKVS	SENFTNNAKTI	RPNNNTRKSINIGPGRALYTTGEIIGDIRQAHC	PSSGGDPEIVTHS	CNSTQLFTWNDTRKLNNTGRNITLPCRIKQIINMWQEVGKAMYAPP	TGLLLTRDGGKDTNGTEIFRPG
1214	-1	11-C01	n/a	n/a	n/a	n/a	n/a	n/a	....	....	....	....	....	.........V............
		16-C03	n/a	n/a	n/a	n/a	n/a	n/a	....	....	....	....	....	....
		16-C07	n/a	n/a	n/a	n/a	n/a	n/a	....	....	....	....	....	.........V............
		16-C08	n/a	n/a	n/a	n/a	n/a	n/a	....	....	....	....	....	....
		16-C11	n/a	n/a	n/a	n/a	n/a	n/a	....	....	....	....	....	....
		16-D04	n/a	n/a	n/a	n/a	n/a	n/a	....	....	....	....	....	....
		16-D06	n/a	n/a	n/a	n/a	n/a	n/a	....	....	....	....	....	.........V............
		26-G01	n/a	n/a	n/a	n/a	n/a	n/a	....	....	....	....	....	.....................R
		26-G07	n/a	n/a	n/a	n/a	n/a	n/a	....	....	....	....	....	.........V............
		26-G09	n/a	n/a	n/a	n/a	n/a	n/a	....	....	....	....	....	.........V............
		26-H02	n/a	n/a	n/a	n/a	n/a	n/a	....	....	....	....	....	....
		26-H05	n/a	n/a	n/a	n/a	n/a	n/a	....	....	....	....	....	.........S............
	28	18-G02	n/a	n/a	n/a	n/a	n/a	n/a	.....G...I.........	Y..........	....	....	....	.........D............
		18-G05	n/a	n/a	n/a	n/a	n/a	n/a	....	Y..........	....	....	....................................K.........	.........D............
		18-H02	<0.01	100	>25	34	24.643	50	....	Y..........	....	....	....	.........D............
	114	24-E06	0.041	96	>25	29	>25	26	....	....A..T...	....	....	....	....
		24-F02	n/a	n/a	n/a	n/a	n/a	n/a	....	.......T...	....	....	....	....
		24-F06	n/a	n/a	n/a	n/a	n/a	n/a	....	....A..T...	....	....	....	....
		32-E06	n/a	n/a	n/a	n/a	n/a	n/a	....	....A..T...	....	....	...K..........S...............................	....
		32-E07	<0.01	100	>25	20	>25	33	.....................................R.....................................	.K..A..T...	....	....	...K..........S...............................	....
		32-F04	0.034	99	15.983	53	>25	47	....	.......T...	....	....	....	....
		32-F05	0.040	100	>25	40	>25	30	....	....A..T...	....	....	....	....

Table S7. *Plasma SGS-Derived env Sequences Obtained from HIV-1$_{YU2}$-Infected Humanized Mice and Antibody Sensitivity, Related to Figures 6 and 7 (continued)*

D 1-18 Treatment Group

ID	Day	Virus	Sensitivity 1-18 IC50	1-18 MPI	3BNC117 IC50	3BNC117 MPI	VRC01 IC50	VRC01 MPI	V1 loop / V2 loop	Loop D	V3 loop	CD4 binding loop	V4 loop / β20/β21	β23 / V5/β24
									132–209	274–284	298–331	363–375	385–438	450–471
									TDLRNATNTTSSSWETMEKGEIKNCSFNITTSIRDKVQKEYALFYNLDVVPIDNASYRLISCNTSVITQACPKVS	SENFTNNAKTI	RPNNNTRKSINIGPGRALYTTGEIIGDIRQAHC	PSSGGDPEIVTHS	CNSTQLFTWNDTRKLNNTGRNITLPCRIKQIINMWQEVGKAMYAPP	TGLLLTRDGGKDTNGTEIFRPG
1069	-1	01-62	n/a	n/a	n/a	n/a	n/a	n/a	....	....	....	....	....	....
		01-69	n/a	n/a	n/a	n/a	n/a	n/a	....	....	....	....	....	....
		06-54	n/a	n/a	n/a	n/a	n/a	n/a	....	....	....	....	....	....
		06-56	n/a	n/a	n/a	n/a	n/a	n/a	....	....	....	....	....	....
		06-59	n/a	n/a	n/a	n/a	n/a	n/a	....	....	....	....	.................................I............	....
		06-65	n/a	n/a	n/a	n/a	n/a	n/a	....	....	......E..........................	....	....	....
		06-66	n/a	n/a	n/a	n/a	n/a	n/a	....	....	....	....	....	....
		06-67	n/a	n/a	n/a	n/a	n/a	n/a	....	....	....	....	....	.....................R
		06-70	n/a	n/a	n/a	n/a	n/a	n/a	....	....	....	....	.................................I............	....
	28	03-58	<0.01	96	<0.01	97	0.017	97	....	....	....	....	.................................I............	....
		08-F02	<0.01	100	<0.01	100	0.048	100	....	....	....	....	....	.....................R
		08-F05	n/a	n/a	n/a	n/a	n/a	n/a	....	....	....	....	.................................I............	....
		08-F08	<0.01	96	<0.01	93	0.115	99	....	....	....	....	....	.........D............
		26-A06	n/a	n/a	n/a	n/a	n/a	n/a	....	....	....	....	....	.....................R
		26-A09	n/a	n/a	n/a	n/a	n/a	n/a	....K...................................	....	....	....	....	.....................R
		26-A10	n/a	n/a	n/a	n/a	n/a	n/a	....	....	....	....	....	.....................R
		26-B02	<0.01	91	<0.01	86	0.057	89	....	....	....	....	....	.....................R
		26-B06	n/a	n/a	n/a	n/a	n/a	n/a	....	....	....	....	....	....
		26-B11	n/a	n/a	n/a	n/a	n/a	n/a	....	....	....	....	....	.....................R
1177	-1	34-A05	n/a	n/a	n/a	n/a	n/a	n/a	....	....	....	....	....	.....................R
		34-A12	n/a	n/a	n/a	n/a	n/a	n/a	....	....	....	....	....	.........D............
		34-B01	<0.01	98	<0.01	98	0.017	100	....	....	....	....	....	.....................R
		34-B09	<0.01	99	<0.01	98	0.035	100	....................................I......................................	....	....	....	....	....
		36-A06	n/a	n/a	n/a	n/a	n/a	n/a	....	....	....	....	....	.....................R
1215	-1	34-C01	n/a	n/a	n/a	n/a	n/a	n/a	....	....	....	....	................................K.............	....
		34-C03	n/a	n/a	n/a	n/a	n/a	n/a	....	....	....	....	....	....
		34-D02	n/a	n/a	n/a	n/a	n/a	n/a	....	....	....	....	....	....
		34-D06	n/a	n/a	n/a	n/a	n/a	n/a	....	....	....	....	....	....
		34-D12	n/a	n/a	n/a	n/a	n/a	n/a	....	....	....	....	....	....
	114	21-G08	n/a	n/a	n/a	n/a	n/a	n/a	....	....	....	....	................................K.............	....
		21-G12	n/a	n/a	n/a	n/a	n/a	n/a	....	....	....	....	................................K.............	....
		21-H03	n/a	n/a	n/a	n/a	n/a	n/a	....	....	....	....	................................K.............	....
		29-G08	n/a	n/a	n/a	n/a	n/a	n/a	....K.............................	....	....	....	................................K.............	....
		29-G10	<0.01	100	0.015	99	0.053	100	....	....	....	....	................................K.............	....
		29-G12	<0.01	100	0.012	100	0.049	100	....	....	....	....	................................K.............	....
1239	-1	34-E04	n/a	n/a	n/a	n/a	n/a	n/a	....	....	....	....	....	....
		34-F06	n/a	n/a	n/a	n/a	n/a	n/a	...........R...	....	....	........M....	....	....
		34-F10	n/a	n/a	n/a	n/a	n/a	n/a	....	....	....	....	....	.......E..............
		36-E02	n/a	n/a	n/a	n/a	n/a	n/a	....	....	....	....	....	....
		36-E03	n/a	n/a	n/a	n/a	n/a	n/a	....	....	....	....	....	......K...............
	114	22-A10	<0.01	87	<0.01	88	0.029	87	....	....	....	....	.................................I......T.....	....
		30-A05	n/a	n/a	n/a	n/a	n/a	n/a	....	....	....	....	....	....
		30-B03	n/a	n/a	n/a	n/a	n/a	n/a	....	....	....	....	.................................I............	....

Table S7. *Plasma SGS-Derived env Sequences Obtained from HIV-1$_{YU2}$-Infected Humanized Mice and Antibody Sensitivity, Related to Figures 6 and 7 (continued)*

E Control Group

ID	Day	Virus	Sensitivity 1-18 IC50	1-18 MPI	3BNC117 IC50	3BNC117 MPI	VRC01 IC50	VRC01 MPI	V1 loop / V2 loop	Loop D	V3 loop	CD4 binding loop	V4 loop / β20/β21	β23 / V5/β24
									TDLRNATNTTSSSWETMEKGEIKNCSFNITTSIRDKVQKEYALFYNLDVVPIDNASYRLISCNTSVITQACPKVS	SENFTNNAKTI	RPNNNTRKSINIGPGRALYTTGEIIGDIRQAHC	PSSGGDPEIVTHS	CNSTQLFTWNDTRKLNNTGRNITLPCRIKQIINMWQEVGKAMYAPP	TGLLLTRDGGKDTNGTEIFRPG
1045	-1	12-E03	n/a	n/a	n/a	n/a	n/a	n/a	....	....	....	....	....	.........S............
1045	-1	12-F01	n/a	n/a	n/a	n/a	n/a	n/a	....	....	....	....	....	....
1045	-1	12-F05	n/a	n/a	n/a	n/a	n/a	n/a	....	....	....	....	....	......K...............
1045	-1	17-E11	n/a	n/a	n/a	n/a	n/a	n/a	....	....	....	....	....	.........D............
1045	-1	17-F02	n/a	n/a	n/a	n/a	n/a	n/a	..................E..	....	....	....	....	......K...............
1045	-1	17-F09	n/a	n/a	n/a	n/a	n/a	n/a	....	....	....	....	....	......K...............
1045	28	15-A11	n/a	n/a	n/a	n/a	n/a	n/a	....	....	....	....	................................K.............	....
1045	28	15-B11	n/a	n/a	n/a	n/a	n/a	n/a	....	....	....	....	................................K.............	....
1045	28	20-A02	<0.01	100	<0.01	100	<0.01	100	...............K.............................K.............................	....	....	....	................................K.............	....
1045	28	20-A04	n/a	n/a	n/a	n/a	n/a	n/a	....	....	....	....	.............E..................K.............	....
1045	28	20-A08	<0.01	100	<0.01	100	0.019	100	....	....	....	....	................................K.............	....
1045	28	20-B03	n/a	n/a	n/a	n/a	n/a	n/a	....	....	....	........M....	....	....
1045	28	20-B07	n/a	n/a	n/a	n/a	n/a	n/a	....	....	....	....	....	....
1045	28	20-B09	<0.01	100	<0.01	100	0.020	100	....I..........................	....	....	....	................................K.............	....
1045	114	25-E03	<0.01	100	<0.01	99	0.024	100	....	....	....	....	...K.............................I......T.....	....
1045	114	33-E06	n/a	n/a	n/a	n/a	n/a	n/a	....	....	....	....	.................................I............	.................N....
1045	114	33-F01	<0.01	100	<0.01	100	<0.01	100	..................................G......................................A.	....	....	....	.................................I............	....
1045	114	33-F09	<0.01	99	<0.01	99	0.052	98	....	....	....	....	....	....
1125	-1	13-C03	n/a	n/a	n/a	n/a	n/a	n/a	....	....	....	....	....	....
1125	-1	13-D11	n/a	n/a	n/a	n/a	n/a	n/a	....	....	....	....	....	....
1125	-1	18-C05	n/a	n/a	n/a	n/a	n/a	n/a	....	....	....	....	....	....
1125	-1	18-C11	n/a	n/a	n/a	n/a	n/a	n/a	....	....	....	........T....	....	....
1125	-1	18-D02	n/a	n/a	n/a	n/a	n/a	n/a	....	....	....	....	....	....
1125	-1	18-D03	n/a	n/a	n/a	n/a	n/a	n/a	....	....	----.............................	....	....	....
1125	-1	18-D07	n/a	n/a	n/a	n/a	n/a	n/a	....G...................	....	....	....	....	....
1125	-1	18-D08	n/a	n/a	n/a	n/a	n/a	n/a	....	....	....	....	....	....
1125	-1	18-D09	n/a	n/a	n/a	n/a	n/a	n/a	....	....	....	....	....	....
1125	28	15-H11	n/a	n/a	n/a	n/a	n/a	n/a	.............................N...	....	....	....	....	...........--.........
1125	28	20-G07	<0.01	100	<0.01	100	0.048	96	.............................N...	....	....	....	....	...........--.........
1125	28	20-H04	n/a	n/a	n/a	n/a	n/a	n/a	....	....	....	....	....	...........--.........
1125	28	20-H07	n/a	n/a	n/a	n/a	n/a	n/a	.............................N.........K...................................	....	....	....	....	....
1125	28	28-C06	n/a	n/a	n/a	n/a	n/a	n/a	.............................P...	....	....	....	....	....
1125	28	28-D06	n/a	n/a	n/a	n/a	n/a	n/a	....	....	....	....	....	...........--.........
1125	28	28-D07	n/a	n/a	n/a	n/a	n/a	n/a	....T....................	....	....	....	....	....
1125	28	28-D10	n/a	n/a	n/a	n/a	n/a	n/a	..........--...	....	....	....	....	...........--.........
1125	114	25-G03	<0.01	100	<0.01	100	0.051	100	.............................N...	....	....	....	....	....
1125	114	25-H01	n/a	n/a	n/a	n/a	n/a	n/a	N......................................K........L..........................	..D........	..........H......................	....	...A..	...........--.........
1125	114	25-H08	n/a	n/a	n/a	n/a	n/a	n/a	N......................................K........L..........................	....	....	....	...................K..........................	........S.............
1125	114	33-G01	<0.01	100	<0.01	100	0.036	100	....K........L..........................	....	....	....	...A...............K..........................	....
1125	114	33-G04	n/a	n/a	n/a	n/a	n/a	n/a	N......................................K........L..........................	....	....	....	...A..............E...........................	....
1125	114	33-G12	n/a	n/a	n/a	n/a	n/a	n/a	....	....	....	....	...................K..........................	........S.............
1125	114	33-H06	<0.01	98	0.011	100	0.031	100	.............................N...	....	....	....	....	.........-............
1195	-1	12-H06	n/a	n/a	n/a	n/a	n/a	n/a	....	....	....	....	....	.........D............
1195	-1	12-H08	n/a	n/a	n/a	n/a	n/a	n/a	....	....	....	....	....	....
1195	-1	17-G03	<0.01	100	<0.01	100	<0.01	100	....	....	....	....	....	.....................R
1195	-1	17-G12	n/a	n/a	n/a	n/a	n/a	n/a	....	....	....	....	....	....
1195	-1	17-H02	n/a	n/a	n/a	n/a	n/a	n/a	....	....	....	....	....	.....................R
1195	28	20-C10	n/a	n/a	n/a	n/a	n/a	n/a	....	....	....	....	...................................K..........	....
1195	28	20-D10	<0.01	100	<0.01	100	0.022	100	....	....	....	....	...................................K..........	....
1291	-1	13-A09	n/a	n/a	n/a	n/a	n/a	n/a	....................G..	....	....	....	....	....
1291	-1	18-A02	n/a	n/a	n/a	n/a	n/a	n/a	....	....	....	....	....	...I..................
1291	-1	18-A08	n/a	n/a	n/a	n/a	n/a	n/a	....	....	....	.P...........	....	....
1291	-1	18-A12	n/a	n/a	n/a	n/a	n/a	n/a	....	....	....	....	....	....
1291	-1	18-B02	n/a	n/a	n/a	n/a	n/a	n/a	....T....................	....	....	....	....	....
1291	-1	18-B07	n/a	n/a	n/a	n/a	n/a	n/a	....	....	....	....	....	....
1291	-1	18-B08	n/a	n/a	n/a	n/a	n/a	n/a	....	....	....	....	....	....
1291	-1	18-B12	n/a	n/a	n/a	n/a	n/a	n/a	....	....	....	....	....	....
1291	28	15-E01	<0.01	100	<0.01	100	<0.01	100	....	....	....	....	....	....
1291	28	15-F09	<0.01	100	<0.01	100	0.030	100	....	....	....	.P...........	....	....
1291	28	15-F12	n/a	n/a	n/a	n/a	n/a	n/a	....	....	....	....	................................K.............	....
1291	28	20-E10	n/a	n/a	n/a	n/a	n/a	n/a	....	....	....	.P...........	.................A............................	....
1291	28	20-F09	<0.01	100	<0.01	100	0.012	100	....	....	....	.P...........	....	....
